# Selective *O*‐Acylation of Enol Silyl Ethers with Acyl Fluorides Catalyzed by Fluoride Ions Derived from Potassium Fluoride and 18‐Crown‐6

**DOI:** 10.1002/open.202300300

**Published:** 2024-01-29

**Authors:** Norio Sakai, Kota Watanabe, Haruka Mori, Yuki Maegawa, Ryuki Takeuchi, Yohei Ogiwara, Kento Ishida

**Affiliations:** ^1^ Department of Pure and Applied Chemistry Faculty of Science and Technology Tokyo University of Science (RIKADAI) Noda, Chiba 278-8510 Japan

**Keywords:** Enol silyl ether, Acyl fluoride, *O*-Acylation, Vinyl ester, Fluoride ion

## Abstract

The fluoride ion‐catalyzed selective *O*‐acylation of enol silyl ethers with acyl fluorides using KF and 18‐Crown‐6 is described herein. This catalytic system facilitated the practical and facile reaction of a variety of enol silyl ethers derived from aromatic/aliphatic ketones and aldehydes with acyl fluorides to afford useful and valuable enol esters.

## Graphical abstract

Fluoride ions derived from potassium fluoride and 18‐crown‐6 efficiently catalyzed the selective *O*‐acylation of a variety of enol silyl ethers with aromatic and aliphatic acyl fluorides to produce unique enol ester derivatives.

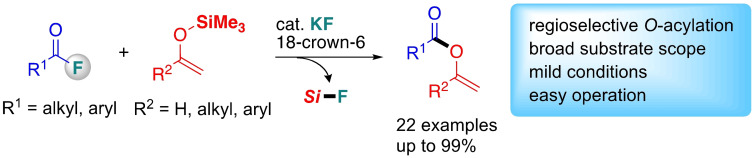

## Introduction

The utility of enol silyl ethers in organic chemistry is primarily associated with the judicious application of reaction intermediates that undergo nucleophilic reactions with a variety of carbon electrophiles. Although there are many studies on acylation using carbonyl electrophiles such as acyl halides and acid anhydrides, most of the studies have focused on *C*‐acylated products because of the formation of the stable 1,3‐diketone structure.[^1^, ^2^] In contrast, the selective and straightforward formation of *O*‐acylated products (enol esters) of enol silyl ethers has not been studied extensively,^[3]^ although these vinyl ester derivatives are a valuable building block for polymerizations^[4]^ and cycloadditions.^[5]^ Moreover, vinyl esters have been recently used as a starting material in an oxidative Heck coupling reaction^[6]^ and as a vinyl source for the C−H vinylation of arenes.^[7]^

Noyori et al. have demonstrated that the treatment of an enol silyl ether with a stoichiometric amount of tris(dimethylamino)‐sulfonium (TAS) difluorotrimethylsiliconate gave a TAS‐enolate.^[8]^ The reaction of this nucleophilic species with acetic anhydride selectively yielded the *O*‐acylated product. Schlosser et al. subsequently reported that the catalytic *O*‐acylation of enol silyl ethers could be realized by treating a mixture of enol silyl ethers derived from aliphatic aldehydes with an acyl fluoride in the presence of tetrabutylammonium fluoride (TBAF).^[9]^ In this context, Vogel and co‐workers examined the TBAF‐catalyzed *O*‐acylation of Danishefsky‐type dienes with acyl fluorides. Haswell et al. reported only one example, wherein the *O*‐acylation of an acetophenone‐derived enol silyl ether with benzoyl fluoride was realized in the presence of a catalytic amount of TBAF.^[10]^ Levacher and co‐workers reported that an organic base, 4‐dimethylaminopyridine (DMAP), catalyzed the *O*‐acylation of enol silyl ethers with benzoyl fluoride.^[11]^ In recent work, Tobisu et al. found that the phosphine‐catalyzed three‐component coupling reaction of acyl fluorides, enol silyl ethers, and alkynes, in which *O*‐acylation between acyl fluorides and enol silyl ethers effectively proceeded.^[12]^ Additionally, Olofson et al. also demonstrated the preparation of vinyl *S*‐phenyl thiocarbonates from enol silyl ethers and phenyl thiofluoroformate in the presence of potassium fluoride (KF) and 18‐crown‐6.^[13]^ In other studies, the Cu(I)‐catalyzed selective *O*‐acylation of enol silyl ethers with acyl chlorides and a related another work with an enol silyl ether, potassium ethoxide, and acetyl chloride were investigated.^[14]^ In particular, Liska et al. reported the synthesis of vinyl esters through the reaction of acyl chlorides and a simple enol silyl ether, trimethyl(vinyloxy)silane, in the presence of KF and 18‐crown‐6.^[15]^ In this case, however, a sort of acyl chlorides employed was limited to a few cases and excess amounts of KF were used.

Considering the high silicon‐fluorine bond energy and the aspect of reusing a liberated fluoride ion, it is reasonable to assume that an acyl fluoride, which has been recently recognized as a remarkably useful synthon of an acyl‐group source,^[16]^ can be employed for developing catalytic and selective *O*‐acylations of enol silyl ethers. However, the fluoride ion‐catalyzed reactions of enol silyl ethers with acyl fluorides are limited to the aforementioned examples, with most cases involving only aldehyde‐derived enol silyl ethers. In particular, the *O*‐acylation of ketone‐derived enol silyl ethers with aliphatic acyl fluorides has not been studied extensively. Therefore, considering the synthetic utility of vinyl ester derivatives, we attempted to re‐examine the fluoride ion‐catalyzed *O*‐acylation of various types of enol silyl ethers with aliphatic or aromatic acyl fluorides. The results, scope, and limitations are reported herein.

## Results and Discussion

Because an enol silyl ether derived from a ketone does not react with an aliphatic acyl fluoride, a model reaction involving 3‐phenylpropionyl fluoride (**1 a**) and 1‐(trimethylsiloxy)styrene (**2 a**), which was derived from acetophenone and trimethylchlorosilane, was chosen initially, and the reaction conditions were optimized (Table 1). In accordance with the conventional study by Schlosser et al.,^[9]^ when the model reaction was conducted with 10 mol% TBAF in a THF solution at 35 °C, the expected *O*‐acylated product **3** was selectively obtained, although in a low yield of 32 % (entry 1). When cesium fluoride (10 mol%) or potassium fluoride (5 mol%) was used, instead of TBAF, the desired esterification proceeded in the presence of the former to give the product in moderate yield (entry 2). In contrast, potassium fluoride was ineffective in this esterification (entry 3). To increase the solubility of KF in THF, 18‐crown‐6, was added as an additive to the reaction mixture. Consequently, the yield improved remarkably to 92 %, and the *O*‐acylation reached completion within 15 min (entries 4 and 5). This reaction proceeded to a considerable extent even when the amount of fluoride was decreased to 2.5 mol% (entry 6). The desired *O*‐acylation did not proceed in the absence of a fluoride source (entry 7).

**Table 1:** Screening of reaction conditions.

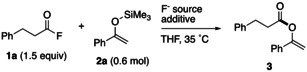				
Entry	F^−^ Source	Additive	Time	Yield^[a]^
	[mol%]	[mol%]	[h]	[%]
1	TBAF (10)^[b]^	–	4	32
2	CsF (10)	–	4	55
3	KF (5)	–	4	NR
4	KF (5)	18‐crown‐6 (5)	4	92 (74)
5	KF (5)	18‐crown‐6 (5)	0.25	99
6	KF (2.5)	18‐crown‐6 (2.5)	4	87
7	–	18‐crown‐6 (5)	2	NR

[a] NMR (Isolated) yield. [b] 1 M THF solution.

With the optimal conditions in hand, the scope of acyl fluorides was examined (Table 2). When 3‐(4‐(trifluoromethyl)phenyl)‐propionyl fluoride was reacted with enol silyl ether **2 a** in the presence of a catalytic amount of KF and 18‐crown‐6, the corresponding vinyl ester **4** was obtained in high yield. In contrast, when employing an aliphatic acyl fluoride bearing a methoxy group on a terminal benzene ring, the desired vinyl ester **5** was not obtained, although most of the starting acyl fluoride was consumed. The reason for this observation is elusive. Probably, the methoxy group, which can strongly coordinate to an electrophilic moiety such as a potassium cation and the silicon atom of **2 a**, prevents the coupling on the O−Si bond of **2 a**.^[17]^ Regardless of the length of the carbon chain, aliphatic acyl fluorides generally gave the corresponding *O*‐acylated products **6** and **7** in good yields. The use of phenylacetyl fluoride, however, led to a slight decrease in the yield of **8**. On the other hand, when aliphatic acyl fluorides bearing a cyclohexyl and an adamantyl group, which are generally considered a bulky substituent, were reacted, the expected *O*‐acylated esters **9** and **10** were obtained in practical yields.

**Table 2:** Substrate scope of acyl fluorides.^[a]^

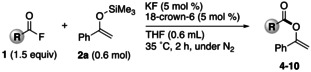		
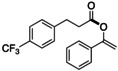		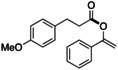
**4**: 73 % (84 %)^[b]^		**5**: trace (conv. 90 %)^[c]^
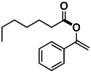	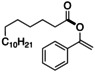	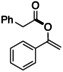
**6**: 50 % (52 %)	**7**: 69 % (77 %)	**8**: 36 % (41 %)^[b]^
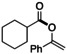		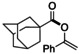
**9**: 58 % (65 %)		**10**: 46 % (53 %)^[c]^

[a] Isolated (NMR) yield. [b] 50 °C, 4 h. [c] KF (10 mol%), 18‐crown‐6 (10 mol%), 50 °C, 4 h.

**Scheme 1:**
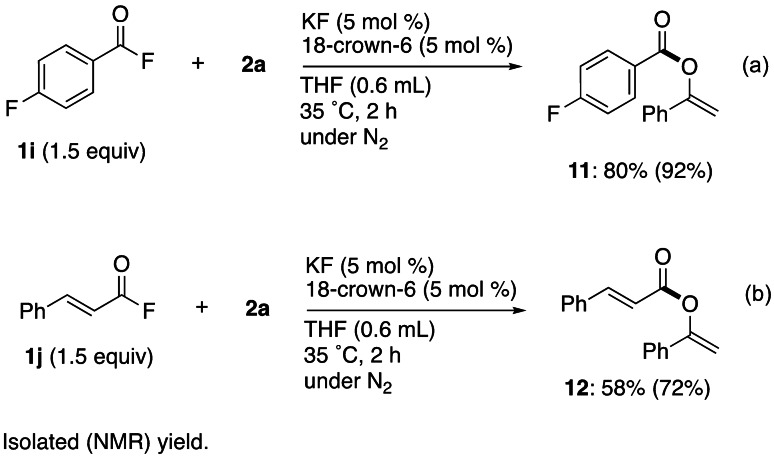
Application to aromatic and vinyl‐type acyl fluorides.

*O*‐Benzoylation between 4‐fluorine‐substituted aromatic acyl fluoride **1 i** and enol silyl ether **2 a** proceeded smoothly under the optimal conditions to afford the desired vinyl benzoate derivative **11** in good yield (Scheme 1a). When cinnamoyl fluoride (**1 j**) was reacted with **2 a** under the same conditions, the corresponding vinyl cinnamate derivative **12** was obtained in 58 % yield (Scheme 1b).

Then, the substrate scope of enol silyl ethers in the reaction with aliphatic acyl fluoride **1 a** was examined under the optimal conditions; the results are shown in Table 3. For enol silyl ethers derived from acetophenone, regardless of the electronic property of the substituents on the benzene ring, the desired *O*‐acylation proceeded selectively to give the corresponding enol esters **13**–**15** in good yields. Unlike that in the attempted synthesis of **5** (Table 2), the methoxy group in the enol silyl ether did not have any influence on the desired *O*‐acylation, and **14** was obtained in high yield. This is probably due to the fact that an extended conjugation between the methoxy group and the benzene ring would decrease the coordination ability. When using aliphatic ketone‐derived enol silyl ethers or an enol silyl ether bearing a cyclohexenyl moiety, the expected enol esters **16**, **17**, and **18** were obtained in 78 %, 82 %, and 78 % yields, respectively. Moreover, the expected *O*‐acylation proceeded to give **19** in a practical yield when an enol silyl ether bearing a branched carbon chain was used. Interestingly, when the reaction was carried out under our conditions with an enol silyl ether bearing two silyl ether moieties, the starting material of which was obtained as a side‐product during the synthesis of an enol silyl ether derived from acetone, the desired *O*‐acylation occurred selectively at the vinyl side to afford the corresponding enol ester **20** in 78 % yield.

**Table 3:** Substrate scope of enol silyl ethers derived from ketones.^[a]^

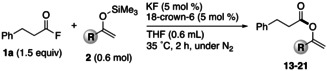		
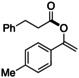	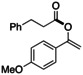	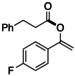
**13**: 70 % (92 %)	**14**: 60 % (86 %)	**15**: 62 % (99 %)
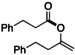	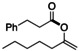	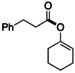
**16**: 78 % (94 %)	**17**: 82 % (83 %)	**18**: 78 % (89 %)
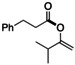	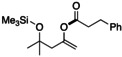	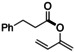
**19**: 56 % (64 %)^b^	**20**: 58 % (78 %)	**21**: 93 % (97 %)

[a] Isolated (NMR) yield. [b] Acyl fluoride **1 a** (1.2 equiv).

Moreover, this catalytic system was effective for enol silyl ethers bearing a diene structure, furnishing the expected enol ester **21** in high yield. This result is in agreement with that reported by Vogel et al. on the TBAF‐catalyzed *O*‐acylation of Danishefsky's diene with acyl fluorides.^[10a]^

The further utility of this catalytic system for the *O*‐acylation between several acyl fluorides and the simplest enol silyl ether **2 k** derived from acetaldehyde was examined (Table 4). In the reaction of aliphatic acyl fluoride **1 a** with **2 k** in the presence of the KF/18‐crown‐6 catalytic system, the desired vinyl ester **22** was obtained in 80 % isolated yield. When using a branched aliphatic acyl fluoride bearing a cyclopropyl ring, the selective *O*‐acylation of **2 k** proceeded quantitatively, affording the corresponding vinyl ester **23** in good yield; the cyclopropyl ring was retained after purification. When cinnamoyl fluoride was also used, the formation of the corresponding vinyl ester **24** was observed in nearly quantitative yield. However, the isolated yield decreased because the separation of **24** from the starting acyl fluoride during the common purification was difficult.

**Table 4:** Application to the synthesis of vinyl ester derivatives.^[a]^

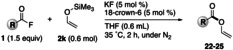		
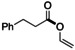	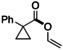	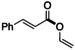
**22**: 80 % (84 %)	**23**: 74 % (98 %)	**24**: 32 % (99 %)

[a] Isolated (NMR) yield.

**Scheme 2:**
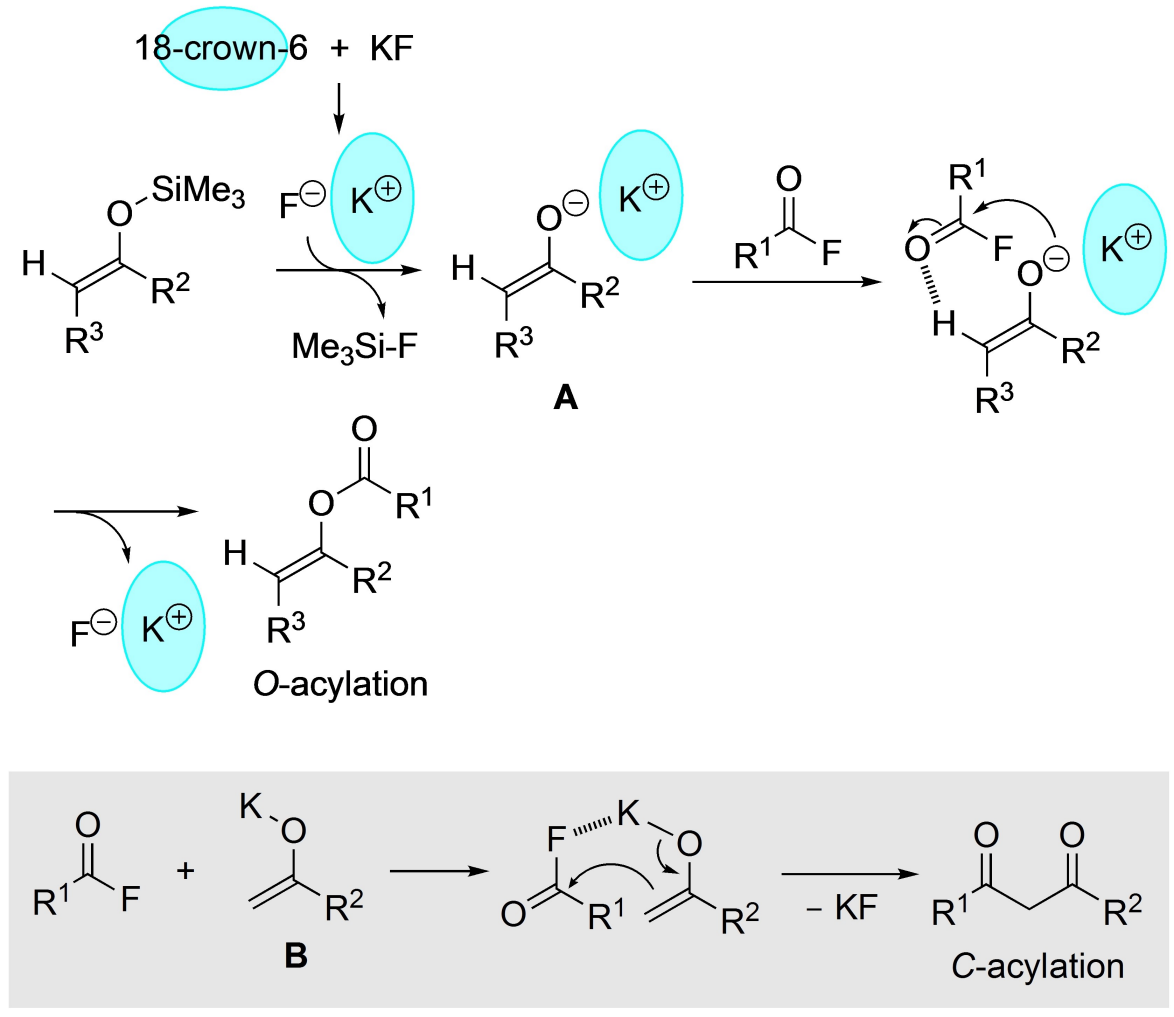
Plausible reaction mechanism of *O*‐acylation.

We finally anticipated the plausible mechanism of the reaction. Initially, a 1 : 1 complex was formed between the potassium cation and 18‐crown‐6, followed by the reaction of the liberated fluoride ion with an enol silyl ether to generate the corresponding potassium enolate complex along with Me_3_SiF. In fact, the formation of Me_3_SiF was observed through the ^19^F‐NMR (THF‐*d*_8_, δ=−157.6 ppm) and ^29^Si‐NMR (THF‐*d*_8_, δ=34.5 ppm) measurements.^[18]^ As shown in Scheme 2, because 18‐crown‐6 generally forms a stable and bulky K^+^ complex,^[19]^ it is assumed that the in‐situ generated potassium enolate complex **A** would tend to adopt a solvent‐separated ion pair form rather than usual enolate **B** having either a covalent potassium‐oxygen bond or a contact ion pair, the characteristic structure of which would lead to predominant kinetically controlled *O*‐acylation, and not *C*‐acylation (gray box in Scheme 2) with a variety of acyl fluorides.^[20]^

## Conclusions

We have demonstrated the fluoride‐catalyzed selective *O*‐acylation of enol silyl ethers with various acyl fluorides using the KF/18‐crown‐6 catalytic system. Unlike the conventional methods using TBAF, this catalytic system could be used for a variety of enol silyl ethers derived from aromatic/aliphatic ketones and formaldehyde. This study presents a practical and facile method to synthesize valuable enol esters and vinyl esters that have potential applications in material chemistry.

## Experimental Section

**A typical experimental procedure for the fluoride‐catalyzed** ***O*** **‐acylation of enol silyl ethers with acyl fluorides**: To a screw‐capped test tube, spray‐dried KF (0.030 mmol, 1.7 mg), 18‐crown‐6 (0.030 mmol, 7.9 mg), THF (0.60 mL), acyl fluoride **1** (0.90 mmol, 1.5 equiv) and enol silyl ether **2** (0.6 mmol) were successively added inside a glove‐box. The mixture was stirred at 35 °C in a water bath for 2 h. After the reaction, the mixture was directly poured into a separatory funnel containing H_2_O (5 mL). The aqueous layer was extracted with CHCl_3_ (5 mL×3). The combined organic layer was dried over anhydrous Na_2_SO_4_. The mixture was filtered and concentrated. The crude product was purified by silica gel column chromatography (eluent: EtOAc/hexane=1/19 or EtOAc/CHCl_3_/hexane=1/1/9) to afford the corresponding vinyl ester derivative.

## Conflict of interests

The authors declare no conflict of interest.

1

## Supporting information

As a service to our authors and readers, this journal provides supporting information supplied by the authors. Such materials are peer reviewed and may be re‐organized for online delivery, but are not copy‐edited or typeset. Technical support issues arising from supporting information (other than missing files) should be addressed to the authors.

Supporting Information

## Data Availability

The data that support the findings of this study are available in the supplementary material of this article.
