# Supplementary material for: Selective O‐Acylation of Enol Silyl Ethers with Acyl Fluorides Catalyzed by Fluoride Ions Derived from Potassium Fluoride and 18‐Crown‐6
Source: ChemistryOpen. 2024 Jan 29;13(7):e202300300. doi: 10.1002/open.202300300 (PMC11230931; doi:10.1002/open.202300300)

ChemistryOpen

Supporting Information

Selective *O*-Acylation of Enol Silyl Ethers with Acyl Fluorides Catalyzed by Fluoride Ions Derived from Potassium Fluoride and 18-Crown-6

Norio Sakai,* Kota Watanabe, Haruka Mori, Yuki Maegawa, Ryuki Takeuchi, Yohei Ogiwara, and Kento Ishida

Supporting Information

Selective *O*-Acylation of Enol Silyl Ethers with Acyl Fluorides Catalyzed by Fluoride Ions Derived from Potassium Fluoride and 18-Crown-6

Norio Sakai,* Kota Watanabe, Haruka Mori, Yuki Maegawa, Ryuki Takeuchi, Yohei Ogiwara, and Kento Ishida

Department of Pure and Applied Chemistry, Faculty of Science and Technology,

Tokyo University of Science (RIKADAI), Noda, Chiba 278-8510, Japan

sakachem@rs.tus.ac.jp

Table of Contents

**S2-S2**	General methods
**S2-S5**	Preparation of acyl fluorides and its spectral data
**S5-S5**	Preparation of enol silyl ether **2i**
**S6-S12**	Spectral data for the prepared vinyl ester derivatives by the present method
**S13**	References
**S14-S62**	Copies of ^{1}H NMR and ^{13}C NMR spectra

General methods. All reactions were carried out under N_2 atmosphere unless otherwise noted. Tetrahydrofuran (THF) was purified over Na/benzophenone by distillation. Trimethylchlorosilane was distilled over K_2CO_3 prior to use. Each reaction was monitored by TLC analysis of reaction aliquots. Thin layer chromatography (TLC) was effected on silica gel F_{254}, and components were located by observation UV light. Column chromatography was performed using silica gel 60. ^1H NMR spectra were measured at 500/400 MHz using tetramethylsilane as an internal standard (0.00 ppm). ^{13}C NMR spectra were measured at 125/100 MHz using the center peak of chloroform (77.0 ppm). ^{19}F NMR spectra were measured at 376 MHz using the peak of the external reference, α,α,α-trifluorotoluene (-62.6 ppm). Acyl chlorides, spray-dried potassium fluoride (KF), and potassium bifluoride (KHF_2) were commercially available and were used after common distillation and drying under heating prior to use. Acyl fluorides **1** and enol silyl ethers **2** were prepared by the modified method in the literature and spectroscopic data of these were in agreement with previously reported data. Enol silyl ether **2k** was purchased from common commercial suppliers and used as received.

1. Preparation of acyl fluorides

Method A: Reaction of an acyl chloride with KF as a fluorine source [1]

In a two-neck flask (200 mL), spray-dried potassium fluoride (3.49 g, 60.0 mmol) was suspended in anhydrous acetonitrile (30 mL), followed by an acyl chloride (30 mmol). The reaction was stirred at 50˚C for 72 h under a nitrogen atmosphere. After 72 h, the reaction mixture was then filtered, and the filtrate was evaporated under reduced pressure. The crude product was purified by distillation under reduced pressure to afford the corresponding acyl fluoride.

Method B: Reaction of an acyl chloride with KHF_2 as fluorine source (method B) [2]

A 100 mL round-bottomed flask was charged with potassium bifluoride (4.69 g, 60.0 mmol) and water (12 mL), and was stirred at room temperature for 1h. Then, tetrabutylammonium chloride (83.3 mg, 0.300 mmol, 1 mol%), CH_2Cl_2 (10 mL, two-fold volume of an acyl chloride), and acyl chloride (30 mmol) were added, and the mixture was further stirred at room temperature for 1h. Then, the mixture was extracted with CH_2Cl_2 (30 mL x 3). The combined extracts were dried over anhydrous $MgSO_4$. The mixture was then filtered and concentrated under reduced pressure. The crude product was purified by distillation under reduced pressure to afford the corresponding acyl fluoride.

3-Phenylpropanoyl fluoride (1a) [3]

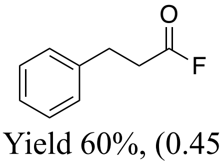

Yield 60%, (0.45 g, 3.0 mmol), method A; a colorless oil; ^1H NMR (500 MHz, $CDCl_3$) δ 7.31 (t, J = 7.5 Hz, 2H, ArH), 7.23 (t, J = 7.5 Hz, 1H, ArH), 7.20 (d, J = 7.5 Hz, 2H, ArH), 2.98 (td, J = 7.5, 1 Hz, 2H, CH_2), 2.81 (td, J = 7.5, 1 Hz, 2H, CH_2); ^{13}C NMR (126 MHz, $CDCl_3$) δ 162.8 (d, J_{C-F} = 361 Hz), 138.9, 128.7, 128.2, 126.8, 33.8 (d, J_{C-F} = 51.7 Hz), 29.9; ^{19}F NMR (471 MHz, $CDCl_3$) δ 45.4; MS (EI), m/z 252 (M^+).

3-(4-Trifluoromethylphenyl)propanoyl fluoride (1b) [4]

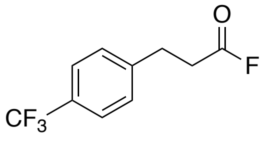

Yield 40%, (0.635 g, 2.88 mmol), method B; a pale red oil; ^{1}H NMR (400 MHz, $CDCl_3$) δ 7.59-7.57 (m, 2H, ArH), 7.35-7.30 (m, 2H, ArH), 3.07-3.00 (m, 2H, CH_2), 2.88-2.83 (m, 1H, CH$\underline{H}$CO), 2.78-2.75 (m, 1H, C$\underline{H}$HCO); ^{13}C NMR (100 MHz, $CDCl_3$) δ 162.4 (d, $J_{C\text{-}F}$ = 358 Hz, COF), 143.1 (d, $J_{C\text{-}F}$ = 69 Hz), 129.3 (q, $J_{C\text{-}F}$ = 32 Hz), 128.7, 125.7 (q, $J_{C\text{-}F}$ = 3 Hz), 124.1 (q, $J_{C\text{-}F}$ = 270 Hz), 33.4 (d, $J_{C\text{-}F}$ = 51 Hz), 29.8 (d, $J_{C\text{-}F}$ = 14 Hz); ^{19}F NMR (376 MHz, $CDCl_3$) δ 45.5 (COF), -62.4 (CF_3); MS (EI), *m/z* 220 (M^+).

3-(4-Methoxyphenyl)propanoyl fluoride (1c) [5]

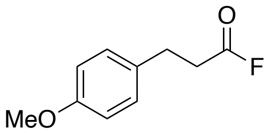

Yield 58%, (1.05 g, 5.81 mmol), method B; a pale yellow oil; ^{1}H NMR (400 MHz, $CDCl_3$) δ 7.14-7.11 (m, 2H, ArH), 6.87-6.83 (m, 2H, ArH), 3.79 (s, 3H, OCH_3), 2.93 (t, *J* = 7.6 Hz, 2H, CH_2), 2.80-2.78 (t, *J* = 7.6 Hz, 2H, CH_2); ^{13}C NMR (100 MHz, $CDCl_3$) δ 162.8 (d, $J_{C\text{-}F}$ = 358 Hz), 158.4, 130.9, 129.2, 114.1, 55.2, 34.2 (d, $J_{C\text{-}F}$ = 49 Hz), 29.1; ^{19}F NMR (376 MHz, $CDCl_3$) δ 45.6; MS (EI), *m/z* 182 (M^+).

Heptanoyl fluoride (1d) [6]

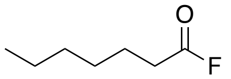

Yield 5%, (0.177 g, 1.33 mmol), method A; A colorless oil; ^{1}H NMR (400 MHz, $CDCl_3$) δ 2.47-2.33 (t, *J* = 6.8 Hz, 2H, CH_2CO), 1.65-1.64 (m, 2H), 1.36-1.30 (m, 6H), 0.89 (t, *J* = 6.8 Hz, 3H, CH_3); ^{13}C NMR (100 MHz, $CDCl_3$) δ 169.6, 35.2, 31.3, 28.5, 24.1, 22.4, 14.0; ^{19}F NMR (376 MHz, $CDCl_3$) δ 45.4; MS (EI), *m/z* 132 (M^+).

Hexadecanoyl fluoride (1e) [6]

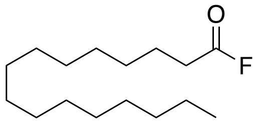

Yield 91%, (7.04 g, 27.3 mmol), method B; a white solid; ^{1}H NMR (400 MHz, $CDCl_3$) δ 2.50 (td, *J* = 7.2, 1.2 Hz, 2H), 1.71-1.63 (m, 2H), 1.38 (brs, 24H), 0.88 (t, *J* = 6.8 Hz, 3H, CH_3); ^{13}C NMR (100 MHz, $CDCl_3$) δ 163.6 (d, $J_{C\text{-}F}$ = 359 Hz), 32.4, 31.92, 31.86, 29.7, 29.6 (overlap x 2), 29.5, 29.4 (overlap x 2), 29.3, 29.1, 28.7, 23.9, 22.7, 14.1; ^{19}F NMR (376 MHz, $CDCl_3$) δ 45.6; MS (EI), *m/z* 258 (M^+).

2-Phenylacetyl fluoride (1f) [2]

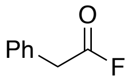

Yield 26%, (0.37 g, 2.7 mmol), method A; A colorless oil; ^{1}H NMR (400 MHz, $CDCl_3$) δ 7.39-7.27 (m, 5H, ArH), 3.80 (brs, 2H, CH_2); ^{13}C NMR (100 MHz, $CDCl_3$) δ 161.4 (d, $J_{C\text{-}F}$ = 361 Hz), 130.7, 129.3, 128.9, 128.0, 38.9 (d, $J_{C\text{-}F}$ = 55 Hz); ^{19}F NMR (376 MHz, $CDCl_3$) δ 45.0; MS (EI), *m/z* 138 (M^+).

Cyclohexanecarbonyl fluoride (1g) [2]

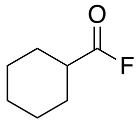

Yield 35%, (1.33 g, 10.2 mmol), method A; a colorless oil; ^{1}H NMR (400 MHz, $CDCl_3$) δ 2.45-2.37 (m, 1H), 1.98-1.94 (m, 2H), 1.81-1.72 (m, 2H), 1.67-1.63 (m, 1H), 1.45-1.44 (m, 2H), 1.35-1.19 (m, 3H); ^{13}C NMR (100 MHz, $CDCl_3$) δ 165.6 (d, $J_{C\text{-}F}$ = 365 Hz), 43.8, 28.3, 25.5, 25.0; ^{19}F NMR (376 MHz, $CDCl_3$) δ 36.8; MS (EI), *m/z* 130 (M^+).

1-Adamantanecarbonyl fluoride (1h) [2]

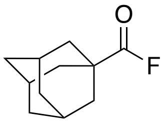

Yield 82%, (3.00 g, 16.4 mmol), method A; a white solid; ^{1}H NMR (500 MHz, $CDCl_3$) δ 2.08-2.01 (brs, 3H), 1.97-1.92 (brs, 6H), 1.75-1.67 (m, 6H); ^{13}C NMR (126 MHz, $CDCl_3$) δ 167.1 (d, $J_{C\text{-}F}$ = 370.4 Hz), 40.5 (d, $J_{C\text{-}F}$ = 50 Hz), 37.8, 36.0, 27.3; ^{19}F NMR (471 MHz, $CDCl_3$) δ 24.0; MS (EI), *m/z* 182 (M^+).

4-Fluorobenzoyl fluoride (1i) [7]

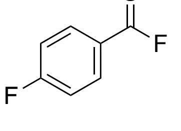

Yield 60%, (0.845 g, 5.95 mmol), method A; a colorless oil; ^{1}H NMR (500 MHz, $CDCl_3$) δ 8.10-8.07 (m, 2H, ArH), 7.23-7.20 (m, 2H, ArH); ^{13}C NMR (126 MHz, $CDCl_3$) δ 167.1 (d, $J_{C\text{-}F}$ = 258.3 Hz), 156.4 (d, $J_{C\text{-}F}$ = 342.7 Hz), 134.2 (dd, $J_{C\text{-}F}$ = 5, 5 Hz), 121.2 (dd, $J_{C\text{–}F}$ = 63, 2.5 Hz), 116.5 (d, $J_{C\text{–}F}$ = 21.4 Hz); ^{19}F NMR (471 MHz, $CDCl_3$) δ 18.1 (COF), -100.5 (FC_6H_5); MS (EI), *m/z* 142 (M^+).

Cinnamoyl fluoride (1j) [2]

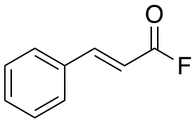

Yield 86%, (0.65 g, 4.3 mmol), method A; a colorless oil; ^{1}H NMR (500 MHz, $CDCl_3$) δ 7.84 (d, *J* = 16 Hz, 1H, -CH=CH-), 7.57-7.56 (m, 2H, ArH), 7.48-7.43 (m, 3H, ArH), 6.37 (dd, *J* = 16, 7.5 Hz, 1H, -CH=CH-); ^{13}C NMR (126 MHz, $CDCl_3$) δ 157.1 (d, $J_{C\text{-}F}$ = 340.2 Hz), 151.4 (d, $J_{C\text{-}F}$ = 6.3 Hz), 133.1, 131.8, 129.1, 128.7, 112.0 (d, $J_{C\text{-}F}$ = 66.8 Hz); ^{19}F NMR (471 MHz, $CDCl_3$) δ 25.7; MS (EI), *m*/*z* 150 (M^+).

1-Phenylcyclopropanecarbonyl fluoride (1k) [8]

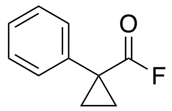

Yield 91%, (1.5 g, 9.1 mmol), method A; a colorless oil; ^{1}H NMR (400 MHz, $CDCl_3$) δ 7.38-7.30 (m, 5H), 1.80 (m, 2H, CH_2), 1.44 (m, 2H, CH_2); ^{13}C NMR (100 MHz, $CDCl_3$) δ 164.9 (d, $J_{C\text{-}F}$ = 349 Hz), 136.6 (d, $J_{C\text{-}F}$ = 1 Hz), 130.2, 128.6, 128.1, 27.1 (d, $J_{C\text{-}F}$ = 62 Hz), 17.7; ^{19}F NMR (376 MHz, $CDCl_3$) δ 24.6; MS (FAB), *m*/*z* 164 (M^+).

2. Preparation of enol silyl ether 2i

Preparation of LDA: A 200 mL four-neck flask was charged with diisopropylamine (3.3 mL, 24 mmol) in 24 mL THF under a nitrogen flow. The solution was then cooled to -78 ˚C, followed by dropwise of *n*-BuLi (15 mL, 24 mmol, 1.6 M in a hexane solution) over a period of 15 min. After the reaction, the bath was removed, and the reaction mixture was stirred for 30 min at 0 ˚C using an ice bath.

Preparation of 2i: Cooling again to -78˚C, to a LDA solution shown above, a THF solution (10 mL) of acetone (20 mmol) was added slowly over a period of 10 min and the reaction mixture was stirred at 0 ˚C for 1 h. Trimethylchlorosilane (4.4 mL, 1.5 equiv) was added to the reaction mixture at 0 ˚C, and the resultant mixture was further stirred for 1 h at room temperature. To quit the reaction, ice water (10 mL) was added to the reaction mixture, and the aqueous layer was extracted with Et_2O (10 mL x 3). The combined organic layer was dried over anhydrous Na_2SO_4 and was evaporated under reduced pressure. The obtained crude oil was purified by distillation under reduced pressure to afford the corresponding enol silyl ether.

4-Methyl-2,4-bis(trimethylsilyloxy)pent-1-ene (2i) [9]

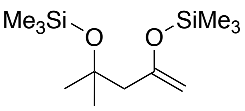

Yield 51% (1.49 g, 5.72 mmol); a colorless oil; ^{1}H NMR (500 MHz, $CDCl_3$) δ 4.08 (s, 1H, C=CH_2), 4.06 (s, 1H, C=CH_2), 2.20 (s, 2H, CH_2), 1.28 (s, 6H, 2Me), 0.21 (s, 9H, $SiMe_3$), 0.11 (s, 9H, $SiMe_3$); ^{13}C NMR (126 MHz, $CDCl_3$) δ 157.0, 92.7, 73.8, 51.8, 29.9, 2.7, 0.04; ^{29}Si NMR (79 MHz, $CDCl_3$) δ 16.7, 7.9; MS (FAB): *m*/*z* 260 (M^+); HRMS (FAB): *m*/*z* calcd for $C_{12}H_{28}O_2Si_2$: 260.1628; found: 260.1629.

3. General Procedure the synthesis of a fluoride-catalyzed *O*-acylation of enol silyl ethers with acyl fluorides

To a screw-capped test tube, spray-dried KF (0.030 mmol, 1.7 mg), 18-crown-6 (0.030 mmol, 7.9 mg), THF (0.6 mL), acyl fluoride **1** (0.90 mmol, 1.5 equiv) and enol silyl ether **2** (0.6 mmol) were successively added in a glovebox. The mixture was stirred at 35 ˚C (a water bath) for 2 h. After the reaction, the mixture was directly poured into a separatory funnel containing H_2O (5 mL). The aqueous layer was extracted with $CHCl_3$ (5 mL x 3). The combined organic layer was dried over anhydrous Na_2SO_4. The mixture was filtered and concentrated. The crude product was purified by silica gel column chromatography (eluent; EtOAc/hexane = 1/19 or EtOAc/$CHCl_3$/hexane = 1/1/9) to afford the corresponding vinyl ester derivative.

1-Phenylethenyl 3-phenylpropanoate (3) [10]

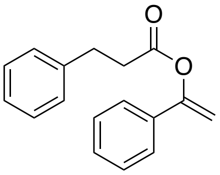

Yield 74% (111 mg) based on the corresponding enol silyl ether (0.59 mmol); a white solid; ^{1}H NMR (400 MHz, $CDCl_3$) δ 7.34-7.25 (m, 10H), 5.45 (d, *J* = 2 Hz, 1H, C=CH_2), 4.96 (d, *J* = 2 Hz, 1H, C=CH_2), 3.06 (t, *J* = 6.4 Hz, 2H, CH_2), 2.88 (t, *J* = 6.4 Hz, 2H, CH_2); ^{13}C NMR (100 MHz, $CDCl_3$) δ 171.0, 152.9, 140.1, 134.1, 128.9, 128.6, 128.5, 128.4, 126.4, 124.8, 102.1, 35.9, 30.9; MS (EI), *m*/*z* 252 (M^+).

1-Phenylethenyl 3-(4-trifluoromethylphenyl)propanoate (4)

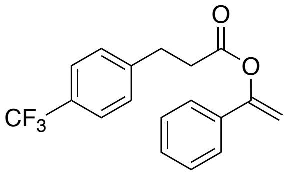

Yield 73% (149 mg) based on the corresponding enol silyl ether (0.64 mmol); a white solid; ^{1}H NMR (500 MHz, $CDCl_3$) δ 7.56 (dd, *J* = 8 H, 2H, $CF_3C_6H_4$), 7.36 (dd, *J* = 8 H, 2H, $CF_3C_6H_4$), 7.32-7.28 (m, 5H, C_6H_5), 5.45 (d, *J* = 2 Hz, 1H, C=CH_2), 4.95 (d, *J* = 2Hz, 1H, C=CH_2), 3.10 (t, *J* = 8 Hz, 2H, CH_2), 2.90 (t, *J* = 8 Hz, 2H, CH_2); ^{13}C NMR (126 MHz, $CDCl_3$) δ 170.6, 152.9, 144.2, 134.0, 129.0, 128.8, 128.7, 127.7, 125.5 (q, J_{C-F} = 3.8 Hz), 124.8, 124.2 (q, J_{C-F} = 272 Hz), 102.2, 35.4, 30.5; ^{19}F NMR (376 MHz, $CDCl_3$) δ -62.2; MS (EI), *m*/*z* 321 (M^+); HRMS (FAB): *m*/*z* calcd for $C_{18}H_{15}F_3O_2$: 321.1107; found: 321.1107.

1-Phenylethenyl heptanoate (6)

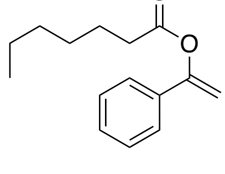

Yield 50% (70 mg); a colorless oil; ^{1}H NMR (400 MHz, $CDCl_3$) δ 7.47-7.45 (m, 2H, ArH), 7.37-7.31 (m, 3H, ArH), 5.47 (d, J = 2 Hz, 1H, C=CH_2), 5.01 (d, J = 2 Hz, 1H, C=CH_2), 2.54 (t, J = 7.6 Hz, 2H, CH_2), 1.77-1.70 (m, 2H), 1.43-1.28 (m, 6H), 0.90 (t, J = 6.4 Hz, 3H, CH_3); ^{13}C NMR (100 MHz, $CDCl_3$) δ 171.9, 152.9, 134.4, 128.8, 128.5, 124.9, 102.0, 34.3, 31.4, 28.8, 24.9, 22.5, 14.0; MS (EI), *m/z* 232 (M^+). HRMS (FAB): *m/z* calcd for $C_{15}H_{20}O_2$: 232.1463; found: 232.1461.

1-Phenylethenyl hexadecanoate (7) [11]

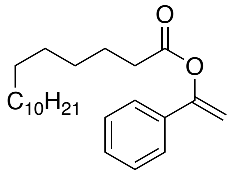

Yield 69% (153 mg) based on the corresponding enol silyl ether (0.62 mmol); a white solid; ^{1}H NMR (400 MHz, $CDCl_3$) δ 7.47-7.45 (m, 2H, ArH), 7.36-7.31 (m, 3H, ArH), 5.46 (d, J = 2.0 Hz, 1H, C=CH_2), 5.01 (d, J = 2.0 Hz, 1H, C=CH_2), 2.53 (t, J = 7.6 Hz, 2H, CH_2), 1.73 (quint, J = 7.6 Hz, 2H), 1.39-1.26 (brs, 24H), 0.88 (t, J = 6.8 Hz, 3H, CH_3); ^{13}C NMR (126 MHz, $CDCl_3$) δ 171.9, 152.9, 134.4, 128.8, 128.5, 124.9, 102.0, 34.3, 31.9, 29.7 (overlap x 2), 29.6 (overlap x 2), 29.4 (overlap x 2), 29.2 (overlap x 2), 29.1 (overlap x 2), 24.9, 22.7, 14.1; MS (FAB), *m/z* 359 (M^+).

1-Phenylethenyl 2-phenylethanoate (8) [12]

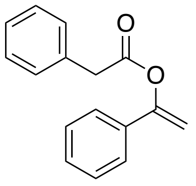

Yield 36% (54 mg) based on the corresponding enol silyl ether (0.62 mmol); a colorless oil; ^{1}H NMR (400 MHz, $CDCl_3$) δ 7.37-7.24 (m, 10H, ArH), 5.45 (d, J = 2 Hz, 1H, C=CH_2), 5.01 (d, J = 2 Hz, 1H, C=CH_2), 3.83 (s, 2H); ^{13}C NMR (126 MHz, $CDCl_3$) δ 169.5, 152.9, 134.1, 133.4, 129.4, 128.9, 128.7, 128.4, 127.4, 124.8, 102.1, 41.5; MS (EI), *m/z* 238 (M^+).

1-Phenylethenyl cyclohexanecarboxylate (9) [11]

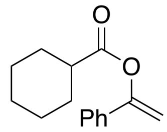

Yield 58% (80 mg) based on the corresponding enol silyl ether (0.56 mmol); a colorless oil; ^{1}H NMR (500 MHz, $CDCl_3$) δ 7.47-7.45 (m, 2H, ArH), 7.36-7.30 (m, 3H, ArH), 5.46 (d, J = 2 Hz, 1H, C=CH_2), 4.99 (d, J = 2 Hz, 1H, C=CH_2), 2.57-2.51 (m, 1H, CH), 2.08-2.05 (m, 2H, CH_2), 1.83-1.81 (m, 2H, CH_2), 1.75-1.65 (m, 1H), 1.61-1.56 (m, 2H), 1.36-1.28 (m, 3H); ^{13}C NMR (126 MHz, $CDCl_3$) δ 174.1, 153.0, 134.5, 128.8, 128.5, 124.9, 101.9, 43.3, 29.0, 25.7, 25.4; MS (EI), *m/z* 230 (M^+).

1-Phenylethenyl 1-adamantanecarboxylate (10)

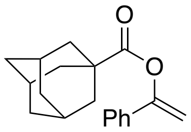

Yield 46% (82 mg) based on the corresponding enol silyl ether (0.63 mmol); a white solid; ^{1}H NMR (500 MHz, $CDCl_3$) δ 7.45 (dd, *J* = 8, 1.5 Hz, 2H, ArH), 7.36-7.31 (m, 3H, ArH), 5.45 (d, *J* = 2 Hz, 1H, C=CH_2), 4.96 (d, *J* = 2 Hz, 1H, C=CH_2), 2.08 (brs, 3H), 2.06-2.05 (brs, 6H), 1.77 (brs, 6H); ^{13}C NMR (126 MHz, $CDCl_3$) δ 175.7, 153.2, 134.6, 128.8, 128.5, 124.8, 101.8, 41.1, 38.8, 36.4, 27.9; MS (FAB), *m*/*z* 282 (M^+); HRMS (FAB): *m*/*z* calcd for $C_{19}H_{22}O_2$: 282.1620; found: 282.1615.

1-Phenylethenyl 4-fluorobenzoate (11) [13]

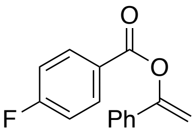

Yield 80% (118 mg) based on the corresponding enol silyl ether (0.58 mmol); a pale pink solid; ^{1}H NMR (500 MHz, $CDCl_3$) δ 8.23-8.20 (m, 2H, FC_6H_4), 7.53-7.51 (m, 2H, C_6H_5), 7.37-7.33 (m, 3H, C_6H_5), 7.20-7.16 (m, 2H, FC_6H_4), 5.59 (d, *J* = 2 Hz, 1H, C=CH_2), 5.16 (d, *J* = 2 Hz, 1H, C=CH_2); ^{13}C NMR (126 MHz, $CDCl_3$) δ 166.1 (d, J_{C-F} = 254.5 Hz), 163.8, 153.1, 134.1, 132.6 (d, J_{C-F} = 10.1 Hz), 129.0, 128.6, 125.6, 124.9, 115.8 (d, J_{C-F} = 21.4 Hz), 102.4; ^{19}F NMR (471 MHz, $CDCl_3$) δ -104.3; MS (EI), *m*/*z* 242 (M^+).

1-Phenylethenyl cinnamate (12) [14]

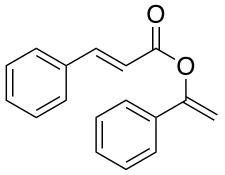

Yield 58% (91 mg) based on the corresponding enol silyl ether (0.63 mmol); a white solid; ^{1}H NMR (500 MHz, $CDCl_3$) δ 7.86 (d, *J* = 16 Hz, 1H, *trans*-PhCH=CH), 7.59-7.55 (m, 2H, ArH), 7.55-7.49 (m, 2H, ArH), 7.45-7.41 (m, 3H, ArH), 7.35-7.31 (m, 3H, ArH), 6.63 (d, *J* = 16 Hz, 1H, *trans*-PhCH=CH), 5.55 (d, *J* = 2 Hz, 1H, C=CH_2), 5.11 (d, *J* = 2 Hz, 1H, C=CH_2); ^{13}C NMR (126 MHz, $CDCl_3$) δ 165.0, 152.9, 146.6, 134.2, 134.1, 130.7, 129.0, 128.9, 128.5, 128.3, 124.9, 117.1, 102.1; MS (FAB): *m*/*z* 250 (M^+).

1-(4-Methylphenyl)ethenyl 3-phenylpropanoate (13)

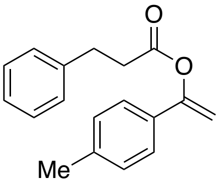

Yield 70% (113 mg) based on the corresponding enol silyl ether (0.61 mmol); a colorless oil; ^{1}H NMR (400 MHz, $CDCl_3$) δ 7.36-7.30 (m, 2H, ArH), 7.26-7.22 (m, 5H, ArH), 7.11-7.09 (m, 2H, ArH), 5.40-5.40 (d, *J* = 1.1 Hz, 1H, C=CH_2), 4.90 (d, *J* = 1.1 Hz, 1H, C=CH_2)), 3.06 (t, *J* = 7.8 Hz, 2H, CH_2), 2.87 (t, *J* = 7.8 Hz, 2H, CH_2), 2.34 (s, 3H); ^{13}C NMR (101 MHz, $CDCl_3$) δ 171.1, 153.0, 140.1, 138.9, 131.3, 129.2, 128.6, 128.4, 126.4, 124.8, 101.2, 35.9, 30.9, 21.2; MS (FAB): *m/z* 266 (M^+); HRMS (FAB): *m/z* calcd for $C_{18}H_{18}O_2$: 266.1307; found, 266.1303.

1-(4-Methoxyphenyl)ethenyl 3-phenylpropanoate (14)

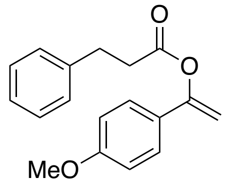

Yield 60% (109 mg) based on the corresponding enol silyl ether (0.65 mmol); a colorless oil; ^{1}H NMR (500 MHz, $CDCl_3$) δ 7.33-7.30 (m, 2H, ArH), 7.27-7.24 (m, 5H, ArH), 6.83-6.80 (m, 2H, $MeOC_6H_4$) 5.32 (d, *J* = 2 Hz, 1H, C=CH_2), 4.84 (d, *J* = 2 Hz, 1H, C=CH_2), 3.80 (s, 3H, MeO), 3.05 (t, *J* = 8 Hz, 2H, CH_2), 2.87 (t, *J* = 8 Hz, 2H, CH_2); ^{13}C NMR (126 MHz, $CDCl_3$) δ 171.1, 160.1, 152.7, 140.1, 128.6, 128.4, 126.7, 126.4, 126.3, 113.8, 100.2, 55.3, 35.9, 30.9; MS (FAB): *m/z* 282 (M^+); HRMS (FAB): *m/z* calcd for $C_{18}H_{18}O_3$: 282.1256; found: 282.1262.

1-(4-Fluorophenyl)ethenyl 3-phenylpropanoate (15)

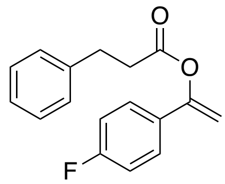

Yield 62% (102 mg) based on the corresponding enol silyl ether (0.61 mmol); a white solid; mp 55.5-56.4˚C; ^{1}H NMR (500 MHz, $CDCl_3$) δ 7.33-7.24 (m, 7H, ArH), 6.98-6.94 (m, 2H, ArH), 5.37 (d, *J* = 2.5 Hz, 1H, C=CH_2), 4.93 (d, *J* = 2.5 Hz, 1H, C=CH_2), 3.05 (t, *J* = 7.5 Hz, 2H, CH_2), 2.87 (t, *J* = 7.5 Hz, 2H, CH_2); ^{13}C NMR (126 MHz, $CDCl_3$) δ 171.0, 163.0 (d, $J_{C\text{-}F}$ = 248.2 Hz), 152.0, 140.0, 130.4 (d, $J_{C\text{-}F}$ = 3.8 Hz), 128.6, 128.4, 126.8 (d, $J_{C\text{-}F}$ = 8.8 Hz), 126.5, 115.5 (d, $J_{C\text{-}F}$ = 22.7 Hz), 101.9, 35.8, 30.8; ^{19}F NMR (471 MHz, $CDCl_3$) δ -112.3; MS (FAB): *m/z* 270 (M^+); HRMS (FAB): *m/z* calcd for $C_{17}H_{15}FO_2$: 270.1056; found: 270.1058.

4-Phenylbut-1-en-2-yl 3-phenylpropanoate (16)

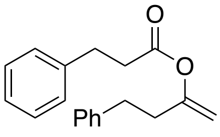

Yield 78% (132 mg) based on the corresponding enol silyl ether (0.61 mmol); a colorless oil; ^{1}H NMR (500 MHz, $CDCl_3$) δ 7.30-7.26 (m, 4H, ArH), 7.23-7.17 (m, 4H, ArH), 7.16-7.14 (m, 2H, ArH),

4.71 (d, J = 1 Hz, 1H, C=CH_2), 4.69 (d, J = 1 Hz, 1H, C=CH_2), 2.97 (t, J = 7 Hz, 2H, CH_2), 2.73-2.67 (m, 4H, CH_2), 2.48 (t, J = 8.5 Hz, 2H, CH_2); ^{13}C NMR (126 MHz, $CDCl_3$) δ 171.0, 155.6, 140.8, 140.1, 128.5, 128.33, 128.31, 128.30, 126.4, 126.0, 101.8, 35.8, 35.0, 32.8, 30.9; MS (FAB): *m/z* 280 (M^+); HRMS (FAB): *m/z* calcd for $C_{19}H_{20}O_2$: 280.1463; found: 280.1461.

Hept-1-en-2-yl 3-phenylpropanoate (17)

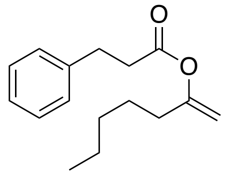

Yield 82% (118 mg) based on the corresponding enol silyl ether (0.58 mmol); a colorless oil; ^{1}H NMR (500 MHz, $CDCl_3$) δ 7.31-7.28 (m, 2H, ArH), 7.25-7.19 (m, 3H, ArH), 4.70 (d, J = 1.5 Hz, 1H, C=CH_2), 4.65 (d, J = 1.5 Hz, 1H, C=CH_2), 2.99 (t, J = 7.5 Hz, 2H, CH_2), 2.73-2.70 (m, 2H, CH_2), 2.14 (t, J = 7.5 Hz, 2H, CH_2), 1.45-1.36 (m, 2H, CH_2), 1.35-1.27 (m, 4H, ArH), 0.88 (t, J = 7.5 Hz, 3H, CH_3); ^{13}C NMR (126 MHz, $CDCl_3$) δ 171.1, 156.6, 140.2, 128.5, 128.3, 126.3, 101.0, 35.9, 33.2, 31.1, 30.9, 26.0, 22.4, 14.0; MS (FAB): *m/z* 246 (M^+); HRMS (FAB): *m/z* calcd for $C_{16}H_{22}O_2$: 246.1620; found: 246.1620.

1-Cyclohexenyl 3-phenylpropanoate (18)

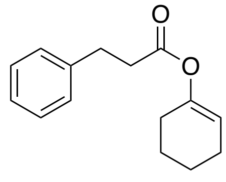

Yield 78% (122 mg) based on the corresponding enol silyl ether (0.68 mmol); a colorless oil; ^{1}H NMR (500 MHz, $CDCl_3$) δ 7.30-7.27 (m, 2H, ArH), 7.22-7.19 (m, 3H, ArH), 5.30 (s, 1H, C=CH-), 2.97 (t, J = 8 Hz, 2H, PhCH_2), 2.69 (t, J = 8 Hz, 2H, CH_2CO), 2.09-2.08 (m, 4H, CH_2), 1.72-1.70 (m, 2H, CH_2), 1.59-1.57 (m, 2H, CH_2); ^{13}C NMR (126 MHz, $CDCl_3$) δ 171.3, 148.3, 140.3, 128.5, 128.3, 126.3, 114.0, 35.9, 30.9, 26.7, 23.6, 22.6, 21.6; MS (FAB): *m/z* 230 (M^+); HRMS (FAB): *m/z* calcd for $C_{15}H_{18}O_2$: 230.1307; found: 230.1310.

1-Isopropylethenyl 3-phenylpropanoate (19)

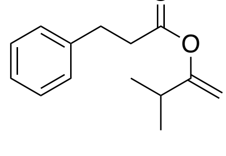

Yield 56% (76 mg) based on the corresponding enol silyl ether (0.62 mmol); a colorless oil; ^{1}H NMR (400 MHz, $CDCl_3$) δ 7.31-7.28 (m, 2H, ArH), 7.24-7.19 (m, 3H, ArH), 4.74 (d, J = 1.6 Hz, 1H C=CH_2), 4.63 (d, J = 1.6 Hz, 1H, C=CH_2), 3.00 (t, J = 7.6 Hz, 2H, PhCH_2), 2.87 (t, J = 7.6 Hz, 2H, CH_2CO), 2.38 (sept, J = 6.8 Hz, 1H, -CH$(CH_3)_2$), 1.02 (dd, J = 6.8, 0.8 Hz, 6H, CH_3); ^{13}C NMR (100 MHz, $CDCl_3$) δ 171.1, 161.3, 140.2, 128.5, 128.3, 126.3, 99.2, 35.9, 32.2, 30.9, 20.1; MS (FAB): *m/z*

219 (M^++H); HRMS (FAB): *m/z* calcd for $C_{14}H_{18}O_2$: 218.1307; found: 218.1309.

4-Methyl-4-((trimethylsilyl)oxy)pent-1-en-2-yl 3'-phenylpropanoate (20)

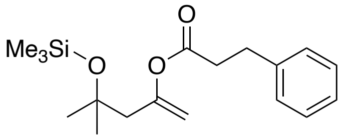

Yield 58% (111 mg); a colorless oil; ^{1}H NMR (500 MHz, $CDCl_3$) δ 7.32-7.29 (m, 2H, ArH), 7.26-7.20 (m, 3H, ArH), 4.78 (m, 2H, C=CH_2), 3.00 (t, *J* = 8 Hz, 2H, PhCH_2), 2.71 (t, *J* = 8 Hz, 2H, CH_2CO), 2.35 (s, 2H, CH_2), 1.25 (s, 6H, CH_3), 0.12 (s, 9H, Si$(CH_3)_3$); ^{13}C NMR (126 MHz, $CDCl_3$) δ 171.0, 153.5, 140.2, 128.5, 128.3, 126.3, 104.8, 73.2, 48.4, 36.1, 30.8, 29.7, 2.5; ^{29}Si NMR (79 MHz, $CDCl_3$) δ 8.7; MS (FAB): *m/z* 321 (M^+); HRMS (FAB): *m/z* calcd for $C_{18}H_{28}O_3Si$: 321.1808; found: 320.1808.

But-1,3-dien-2-yl 3-phenylpropanoate (21)

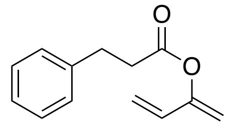

Yield 93% (110 mg) based on the corresponding enol silyl ether (0.58 mmol); a colorless oil; ^{1}H NMR (400 MHz, $CDCl_3$) δ 7.32-7.25 (m, 2H, ArH), 7.25-7.20 (m, 3H, ArH), 6.23 (dd, *J* = 10.8, 6 Hz, 1H, CH_2=CH-), 5.10 (d, *J* = 10.8 Hz, 1H, *trans*-CH_2=CH), 5.08 (d, *J* = 6 Hz 1H, *cis*-CH_2=CH), 5.00 (s, 1H, C=CH_2), 4.87 (s, 1H, C=CH_2), 3.04 (t, *J* = 7.6 Hz, 2H, PhCH_2), 2.82 (t, *J* = 7.6 Hz, 2H, CH_2CO); ^{13}C NMR (100 MHz, $CDCl_3$) δ 170.6, 151.7, 140.0, 130.7, 128.5, 128.3, 126.4, 115.4, 106.1, 35.7, 30.9; MS (FAB): *m/z* 202 (M^+); HRMS (FAB): *m/z* calcd for $C_{13}H_{14}O_2$: 202.0994; found: 202.0997.

Ethenyl 3-phenylpropanoate (22) [15]

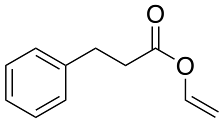

Yield 80% (83 mg) based on the corresponding enol silyl ether (0.59 mmol); a colorless oil; ^{1}H NMR (400 MHz, $CDCl_3$) δ 7.32-7.25 (m, 3H, ArH, CH=CH_2), 7.23-7.20 (m, 3H, ArH), 4.88 (dd, *J* =14.4, 2 Hz, 1H, *trans*-CH_2=CH), 4.57 (dd, *J* = 6.4, 2 Hz, 1H, *cis*-CH_2=CH), 2.99 (t, *J* = 7.6 Hz, 2H, PhCH_2), 2.72 (t, *J* = 7.6 Hz, 2H, CH_2CO); ^{13}C NMR (126 MHz, $CDCl_3$) δ 169.9, 141.1, 140.1, 128.6, 128.3, 126.4, 97.7, 35.5, 30.5; MS (EI), *m/z* 176 (M^+).

Ethenyl 1-phenylcyclopropanecarboxylate (23)

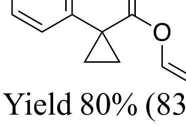

Yield 80% (83 mg); a colorless oil; ^{1}H NMR (400 MHz, $CDCl_3$) δ 7.36-7.19 (m, 6H, ArH, CH=CH_2),

4.71 (dd, J =14, 1.6 Hz, 1H, *trans*-CH_2=CH), 4.47 (dd, J = 6.4, 1.6 Hz, 1H, *cis*-CH_2=CH), 1.70-1.68 (m, 2H, CH_2), 1.28-1.25 (m, 2H, CH_2); ^{13}C NMR (126 MHz, $CDCl_3$) δ 171.7, 141.1, 138.6, 130.1, 128.1, 127.3, 97.5, 28.7, 17.0; MS (EI): *m/z* 188 (M^+); HRMS (FAB): *m/z* calcd for $C_{12}H_{13}O_2$: 189.0916; found: 189.0890 (M^++H).

Vinyl cinnamate (24) [16]

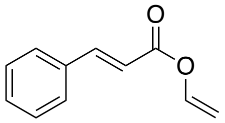

Yield 32% (33 mg); a colorless solid; ^{1}H NMR (400 MHz, $CDCl_3$) δ 7.80 (d, J =16 Hz, 1H, Ph-CH=CH), 7.56-7.53 (m, 2H, ArH), 7.44 (d, J = 6 Hz, 1H, CH=CH_2), 7.42-7.25 (m, 3H, ArH), 6.46 (d, J =16 Hz, 1H, Ph-CH=CH), 4.98 (dd, J =14, 1.6 Hz, 1H, *trans*-CH_2=CH), 4.64 (dd, J = 6, 1.6 Hz, 1H, *cis*-CH_2=CH); ^{13}C NMR (126 MHz, $CDCl_3$) δ 163.9, 146.6, 141.3, 134.1, 130.7, 128.9, 128.3, 116.6, 97.8; MS (EI), *m/z* 174 (M^+).

References

[1] N. Ishikawa, T. Kitazume, T. Yamazaki, Y. Mochida, T. Tatsuno, *Chem. Lett.* **1981**, *10*, 761-764.

[2] M. Tryniszewski, M. Barbasiewicz, *Synthesis* **2022**, *54*, 1446-1460.

[3] F. Beaulieu, L.-P. Beauregard, G. Courchesne, M. Couturier, F. LaFlamme, A. L'Heureux, *Org. Lett.* **2009**, *11*, 5050-5053.

[4] B. Fang, H. Li, M. Peng, L. Ning, X. Chen, J. An, **2021**.

[5] H. Li, M. Peng, Z. Lai, L. Ning, X. Chen, X. Zhang, P. Wang, R. Szostak, M. Szostak, J. An, *Chem. Commun.* **2021**, *57*, 5195-5198.

[6] D. Limat, M. Schlosser, *Tetrahedron* **1995**, *51*, 5799-5806.

[7] Y. Ogiwara, Y. Sakurai, H. Hattori, N. Sakai, *Org. Lett.* **2018**, *20*, 4204-4208.

[8] A. Matsumoto, Z. Wang, K. Maruoka, *J. Org. Chem.* **2021**, *86*, 5401-5411.

[9] a) R. K. Bressin, S. Osman, I. Pohorilets, U. Basu, K. Koide, *J. Org. Chem.* **2020**, *85*, 4637-4647; b) E. V. Boltukhina, A. E. Sheshenev, I. M. Lyapkalo, *Synthesis* **2011**, 3507-3515.

[10] J. Jeschke, C. Gäbler, H. Lang, *J. Org. Chem.* **2016**, *81*, 476-484.

[11] C. S. Yi, R. Gao, *Organometallics* **2009**, *28*, 6585-6592.

[12] B. C. Chary, S. Kim, *J. Org. Chem.* **2010**, *75*, 7928-7931.

[13] J. Tripathy, M. Bhattacharjee, *Tetrahedron Lett.* **2009**, *50*, 4863-4865.

[14] M. Nishiumi, H. Miura, K. Wada, S. Hosokawa, M. Inoue, *ACS Catal.* **2012**, *2*, 1753-1759.

[15] N. Armesto, M. Ferrero, S. Fernández, V. Gotor, *J. Org. Chem.* **2003**, *68*, 5784-5787.

[16] F. Luo, C. Pan, P. Qian, J. Cheng, *Synthesis* **2010**, 2005-2010.

^{1}H NMR of **1a**

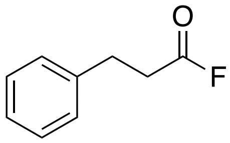

1a

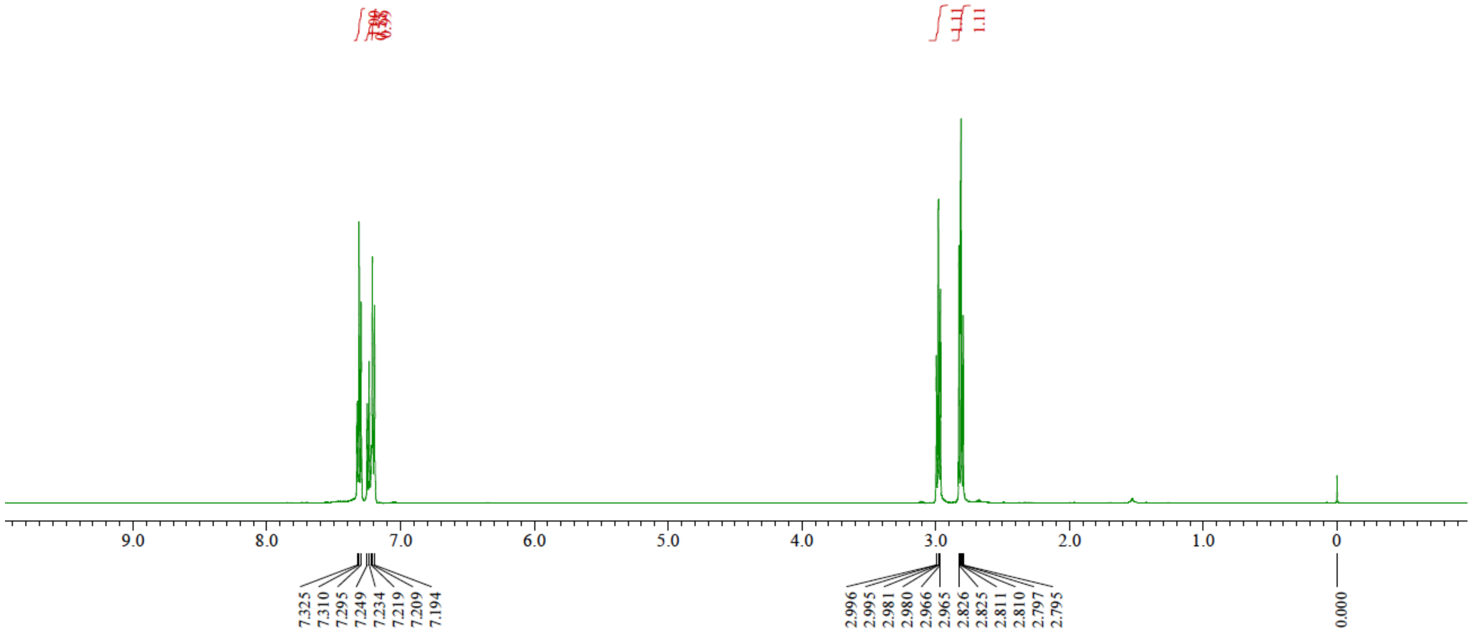

^{13}C NMR of **1a**

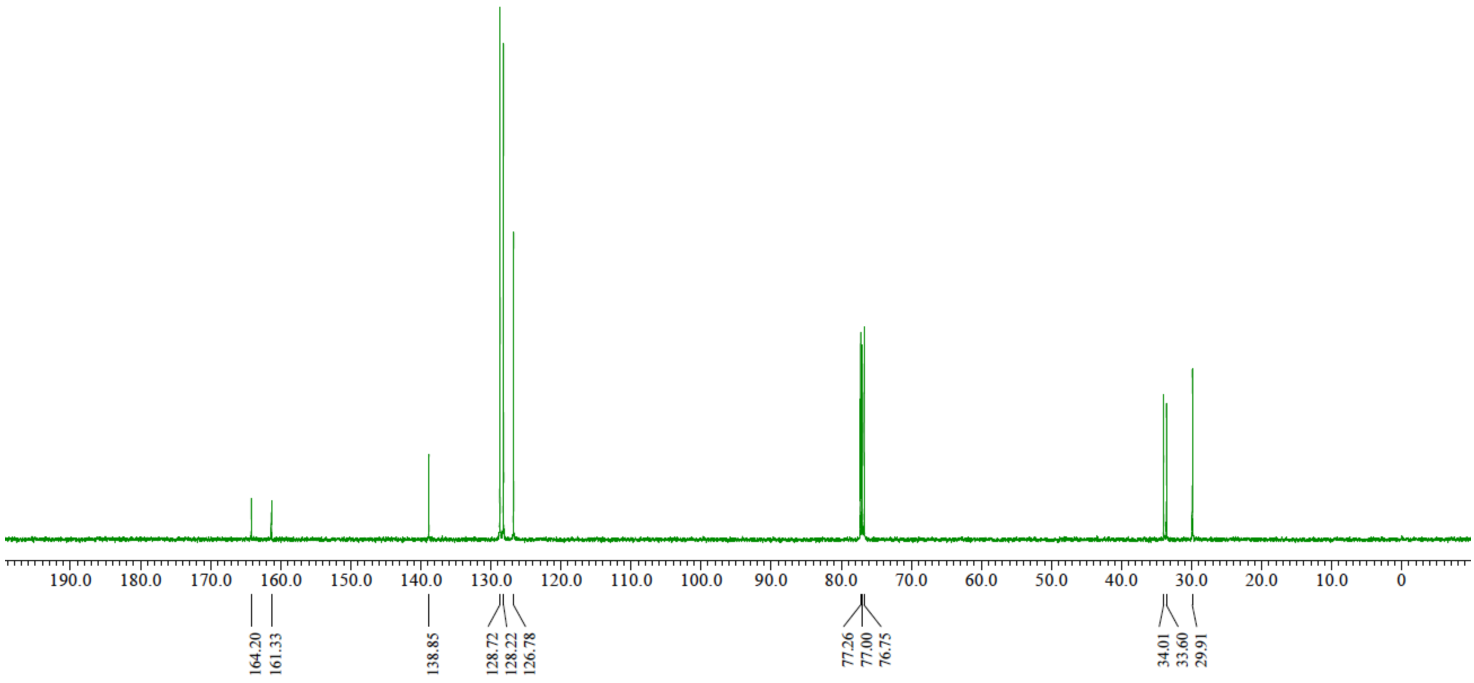

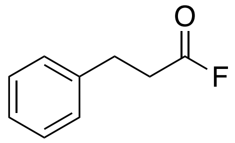

1a

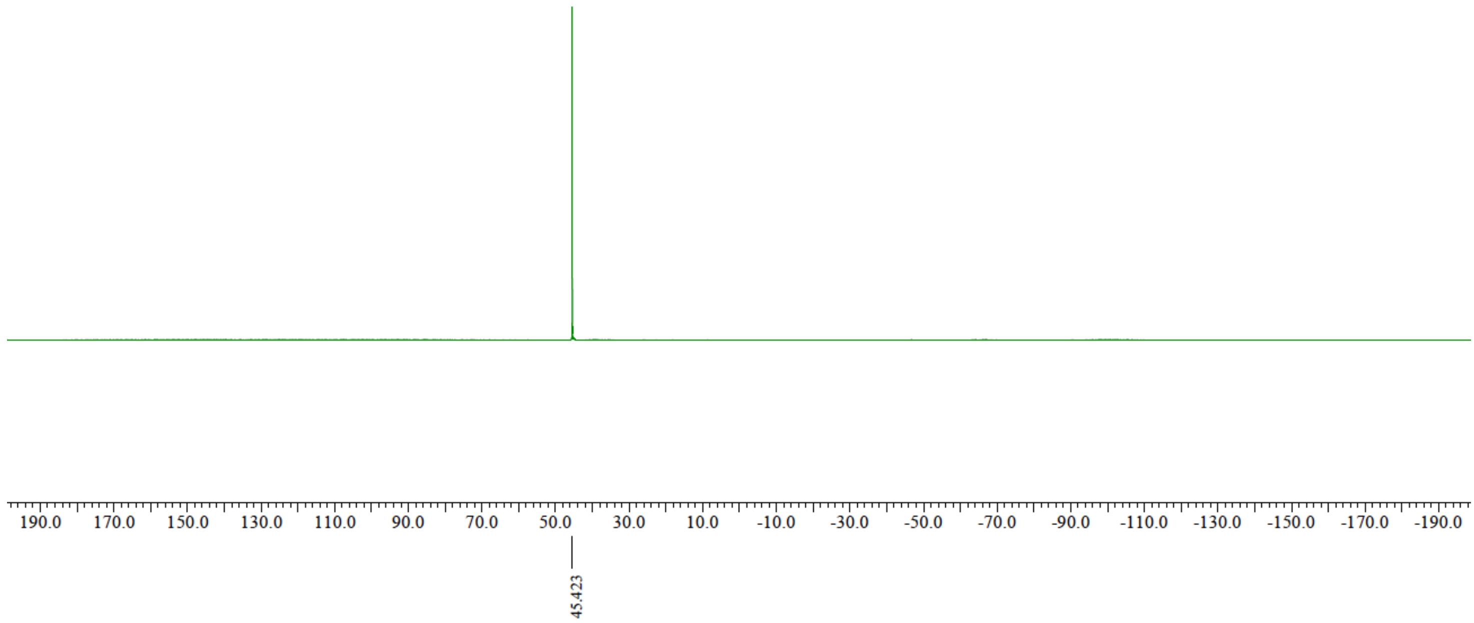

^{19}F NMR of **1a**

^{1}H NMR of **1b**

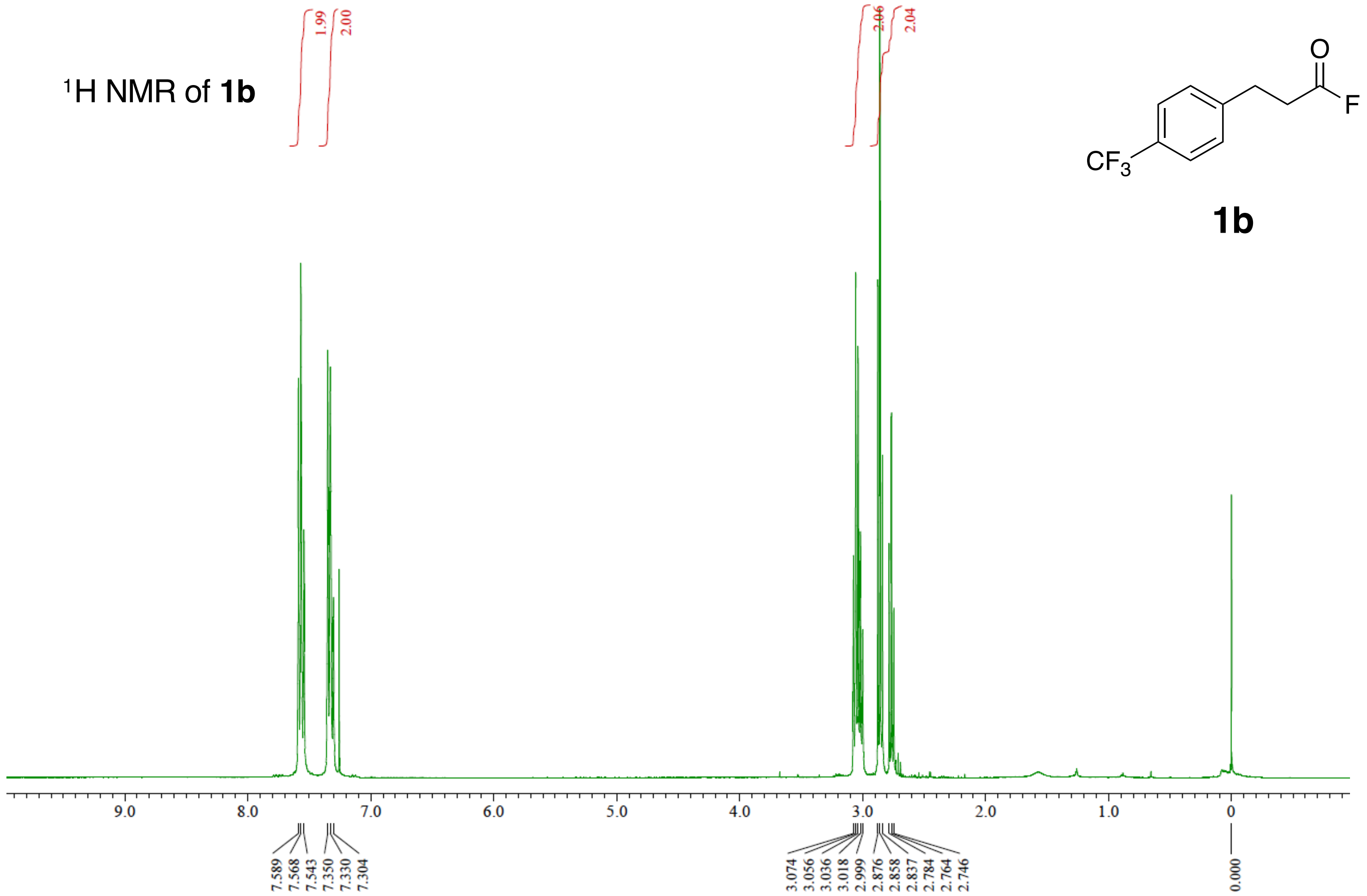

1b

^{13}C NMR of **1b**

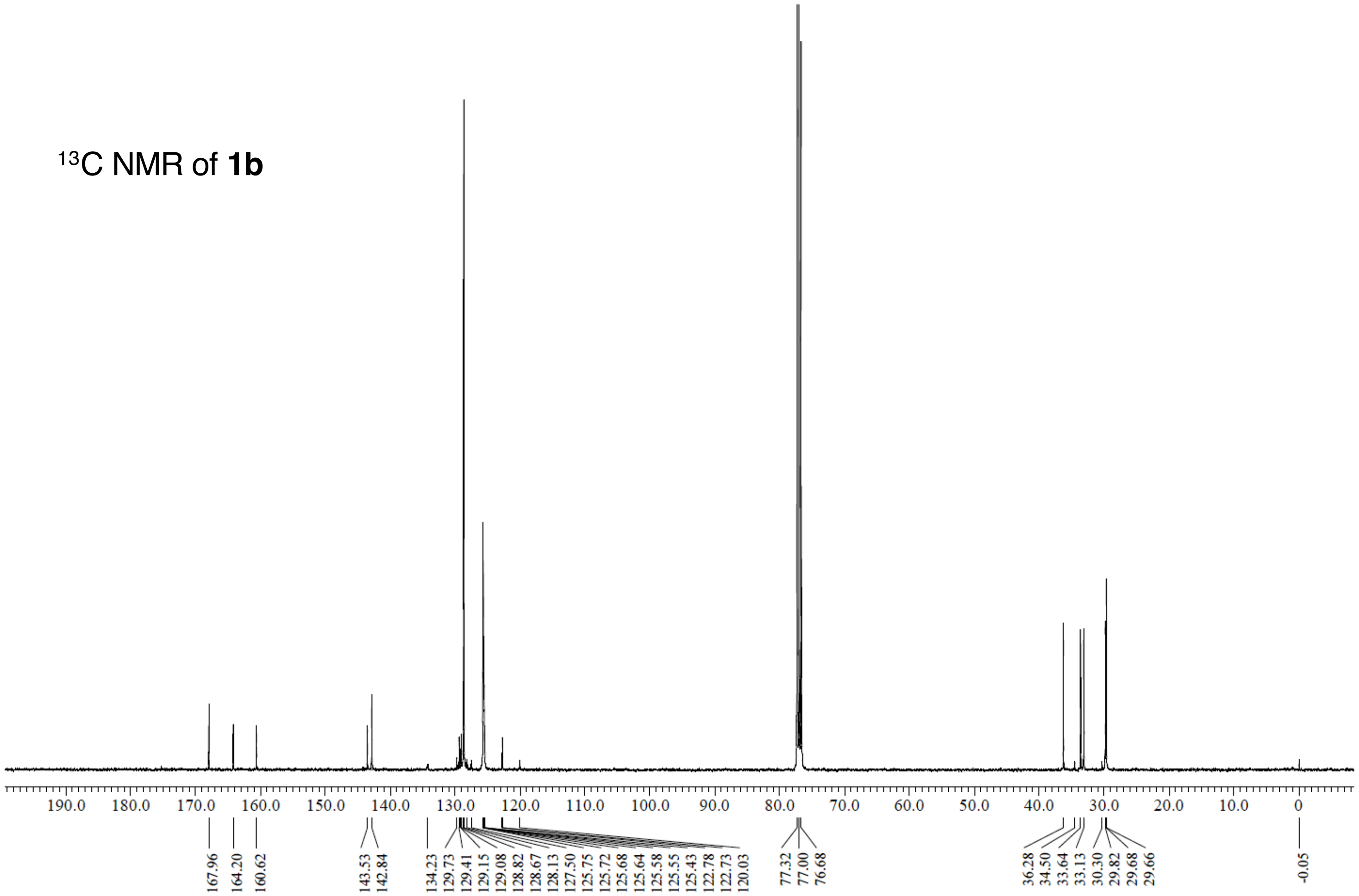

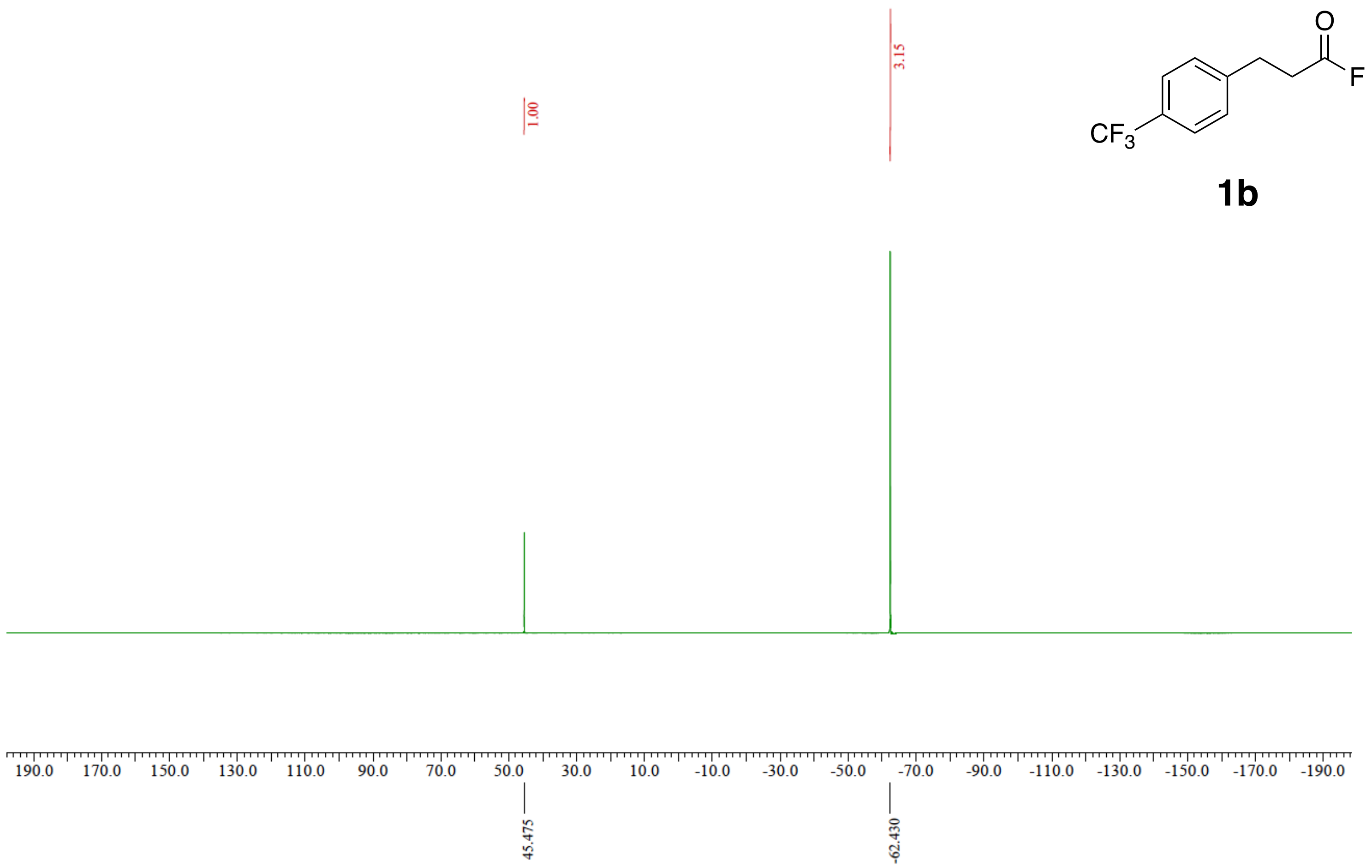

1b

^{19}F NMR of **1b**

^{1}H NMR of **1c**

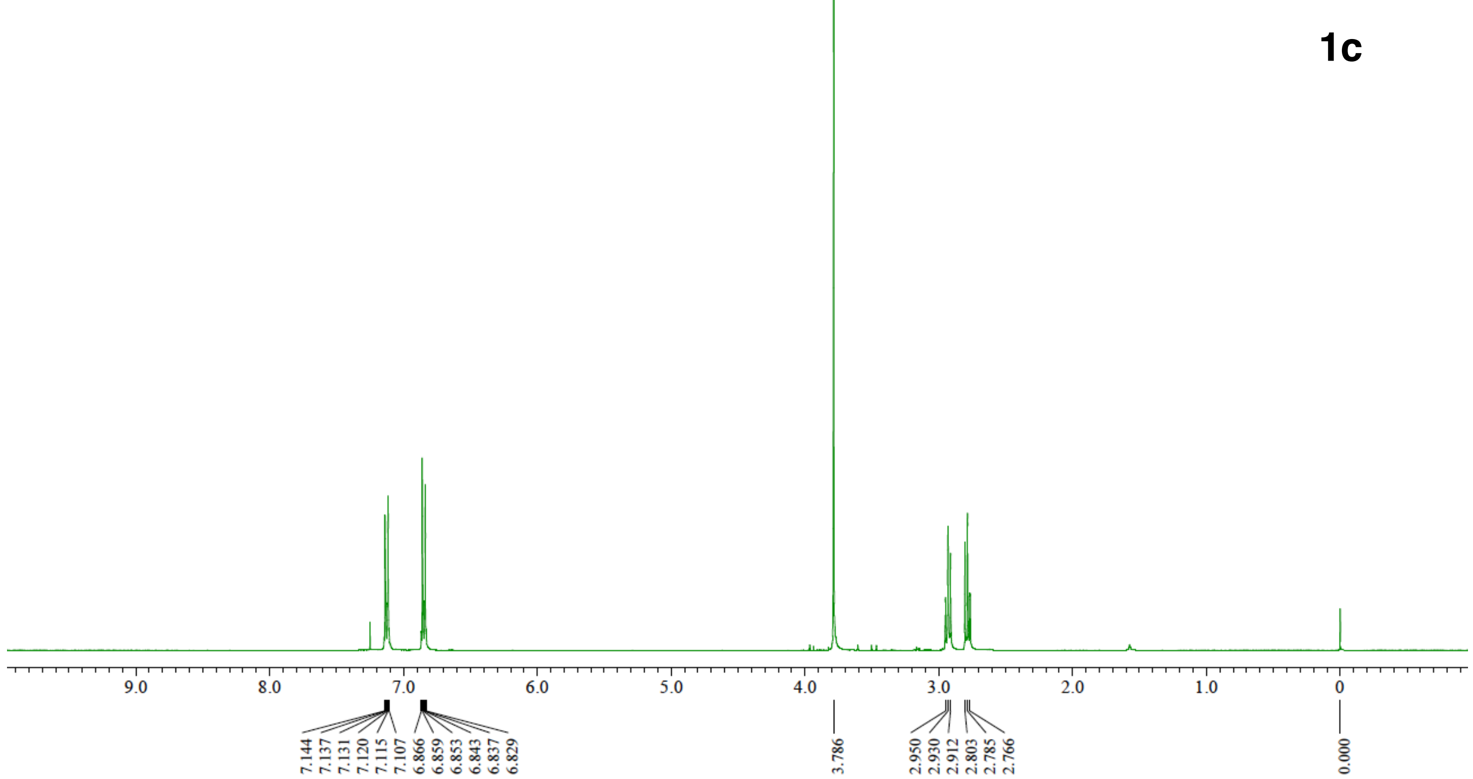

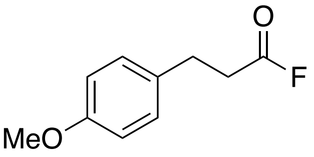

1c

^{13}C NMR of **1c**

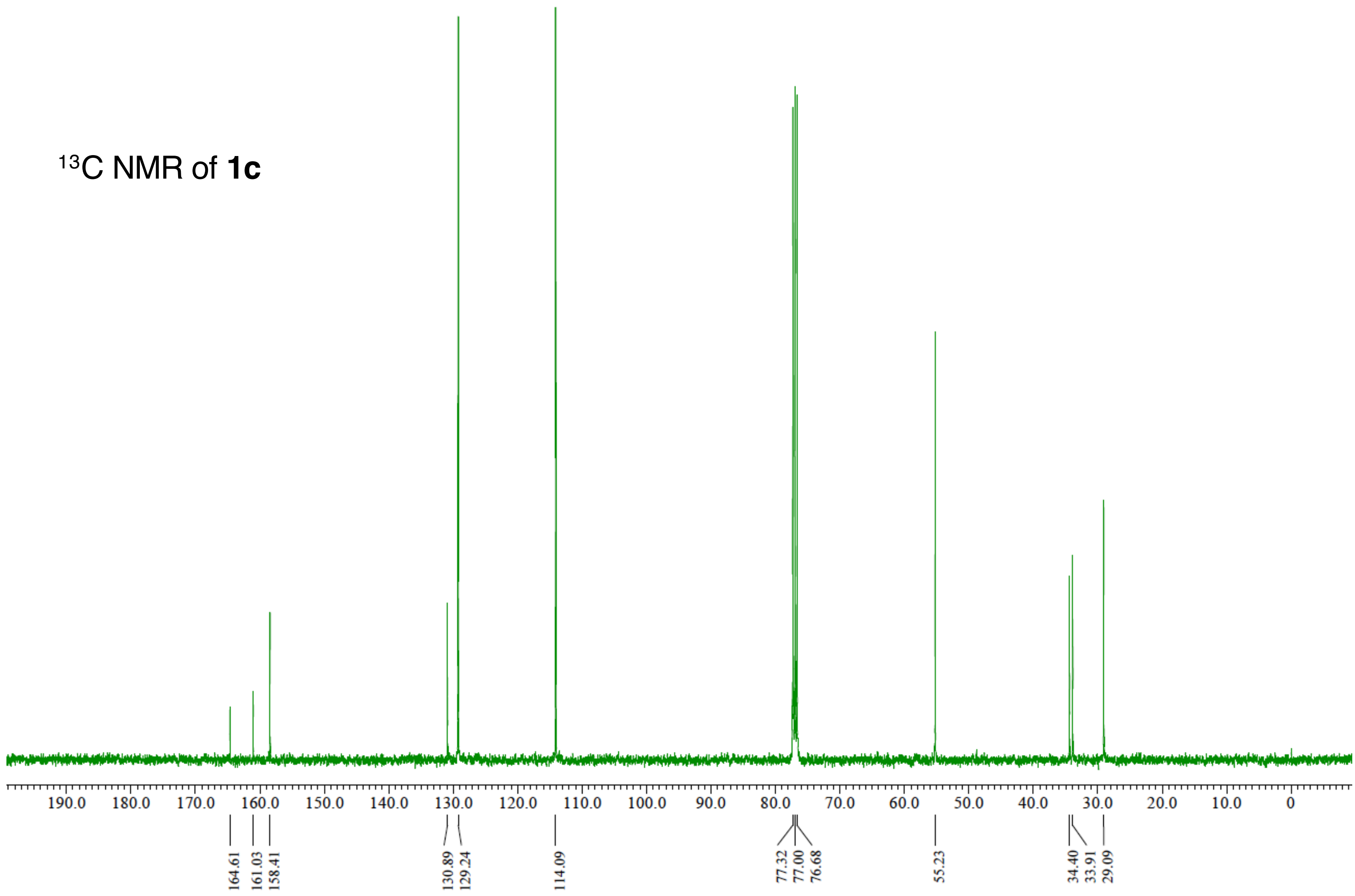

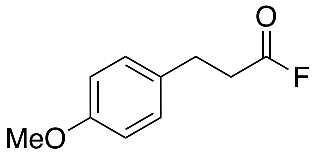

1c

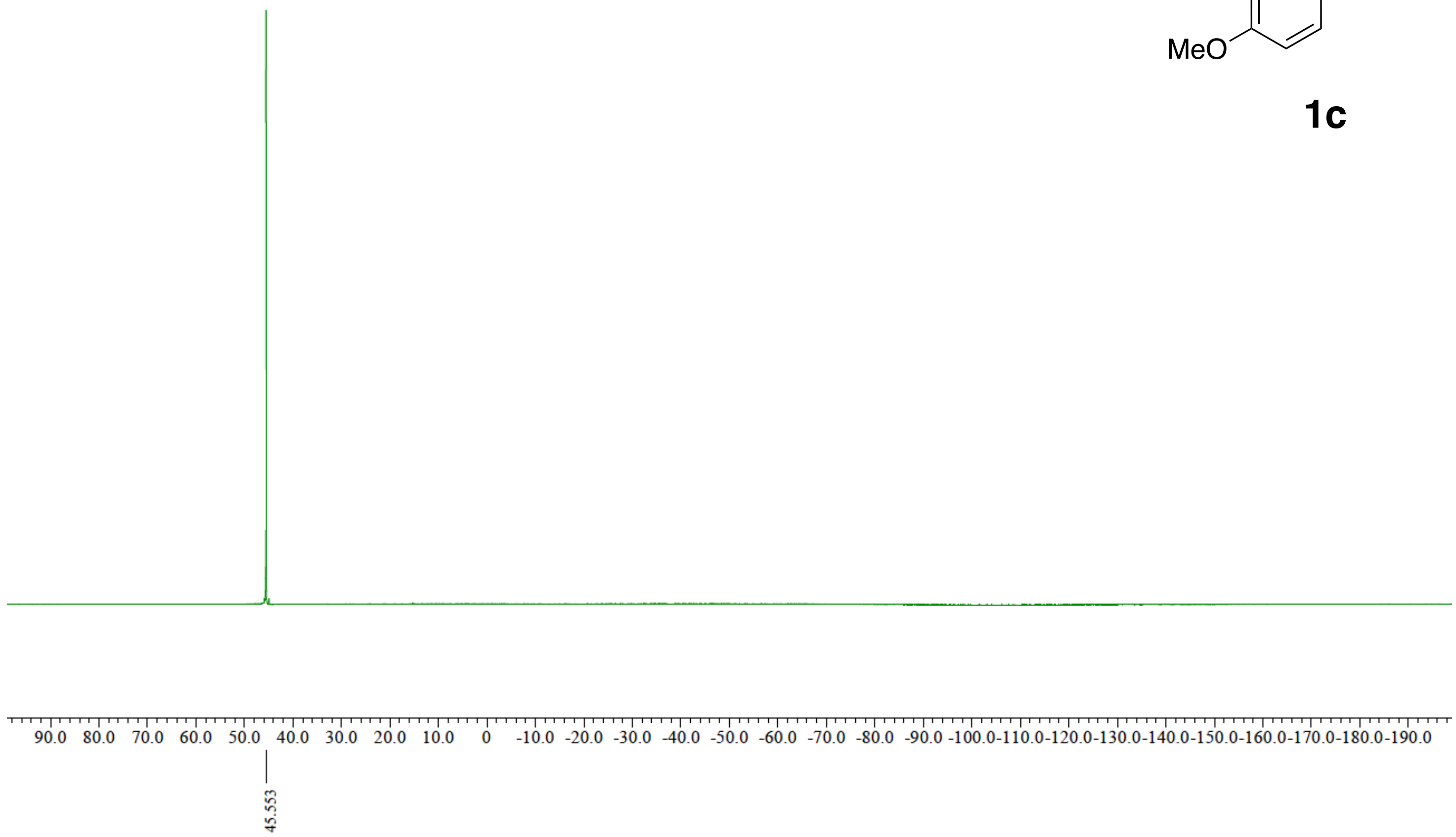

^{19}F NMR of **1c**

^{1}H NMR of **1d**

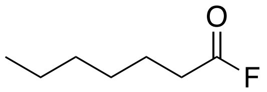

1d

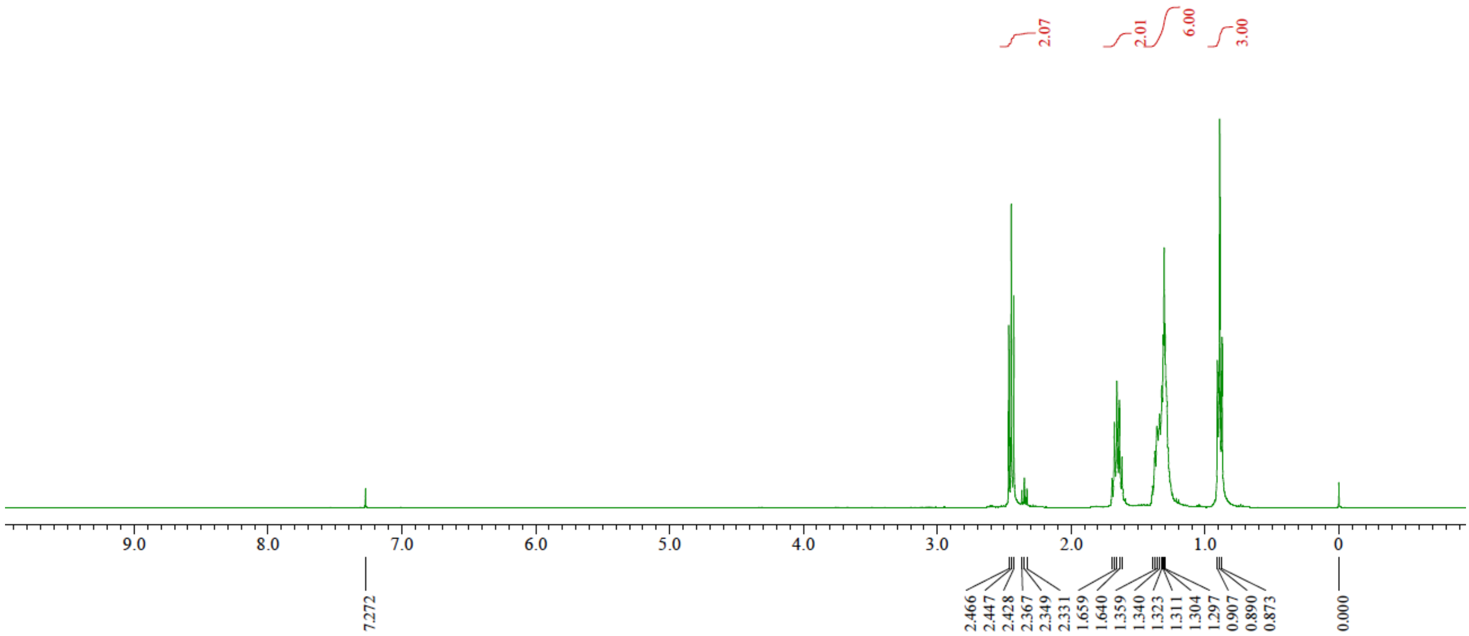

^{13}C NMR of **1d**

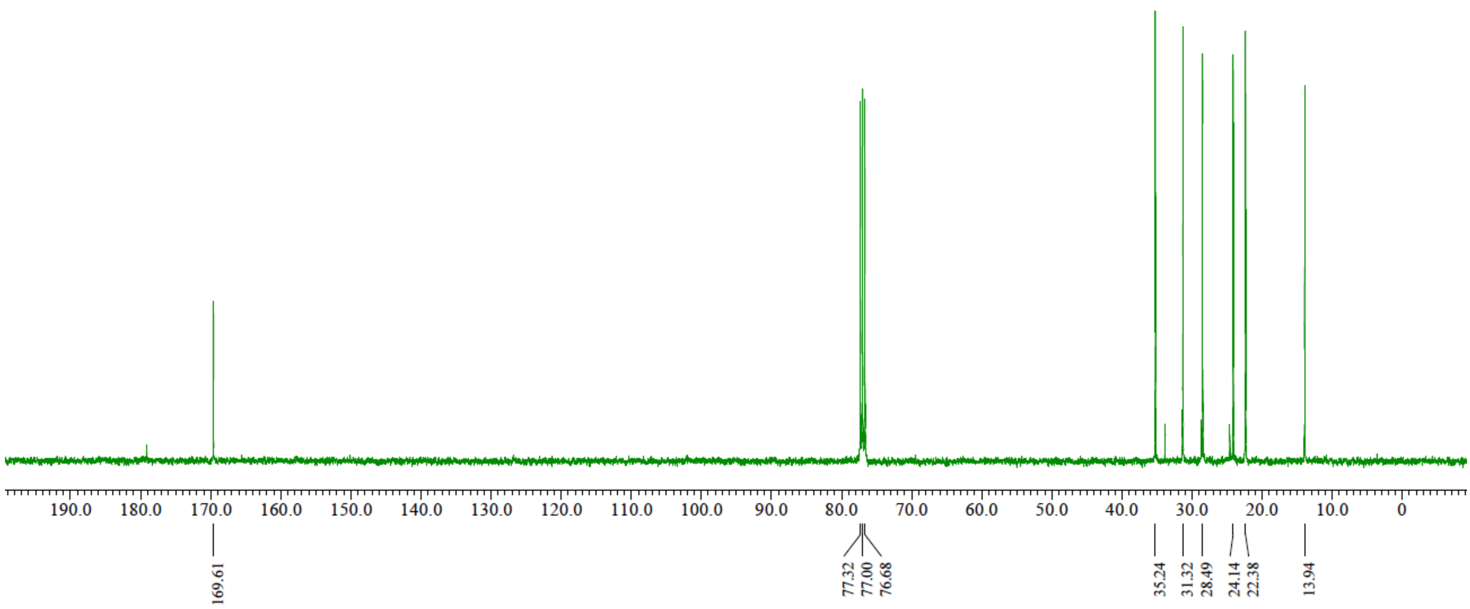

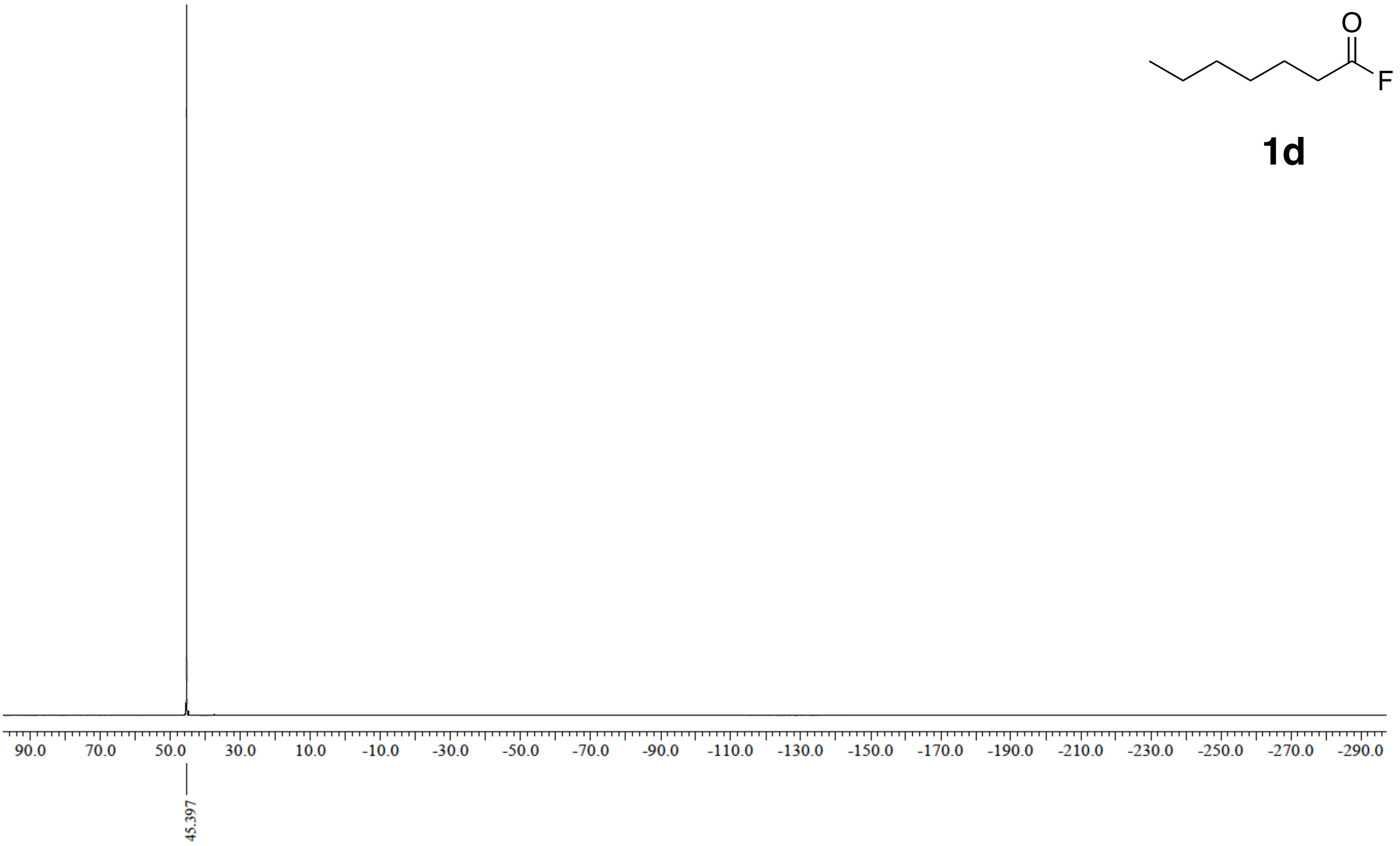
O
F
1d
90.0 70.0 50.0 30.0 10.0 -10.0 -30.0 -50.0 -70.0 -90.0 -110.0 -130.0 -150.0 -170.0 -190.0 -210.0 -230.0 -250.0 -270.0 -290.0
45.397

^{19}F NMR of **1d**

^1H NMR of **1e**

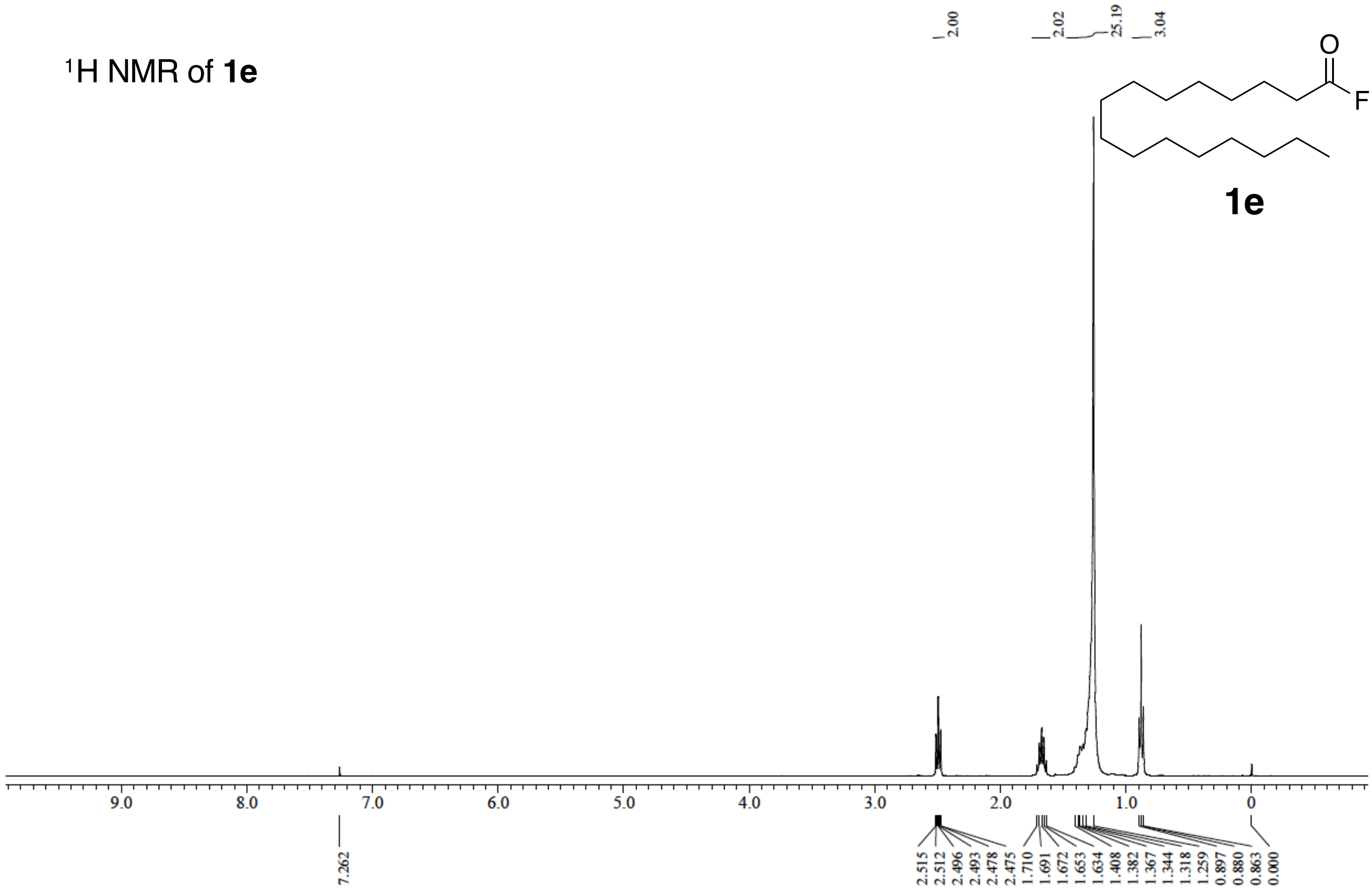

1e

^{13}C NMR of **1e**

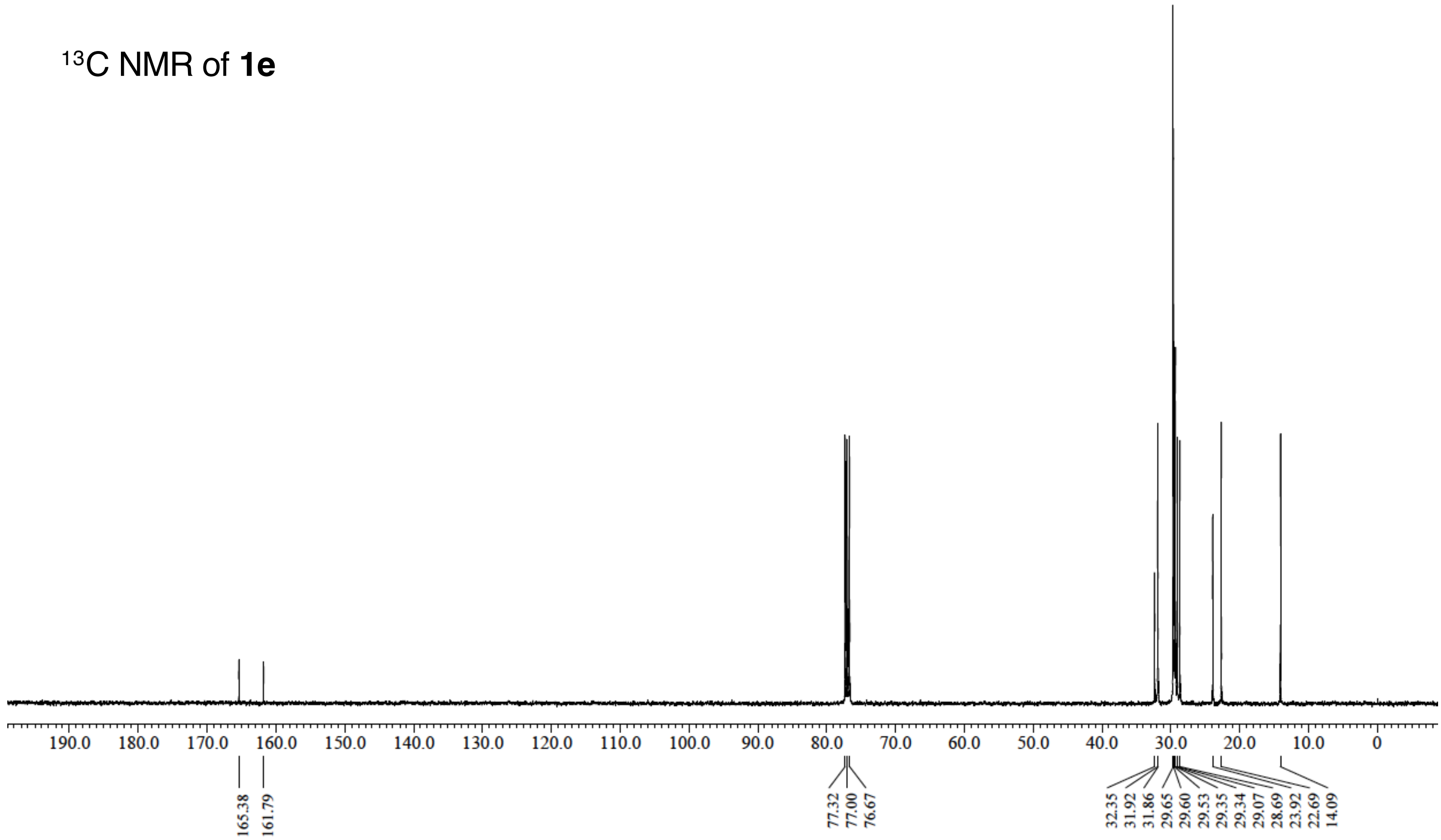

^{19}F NMR of **1e**

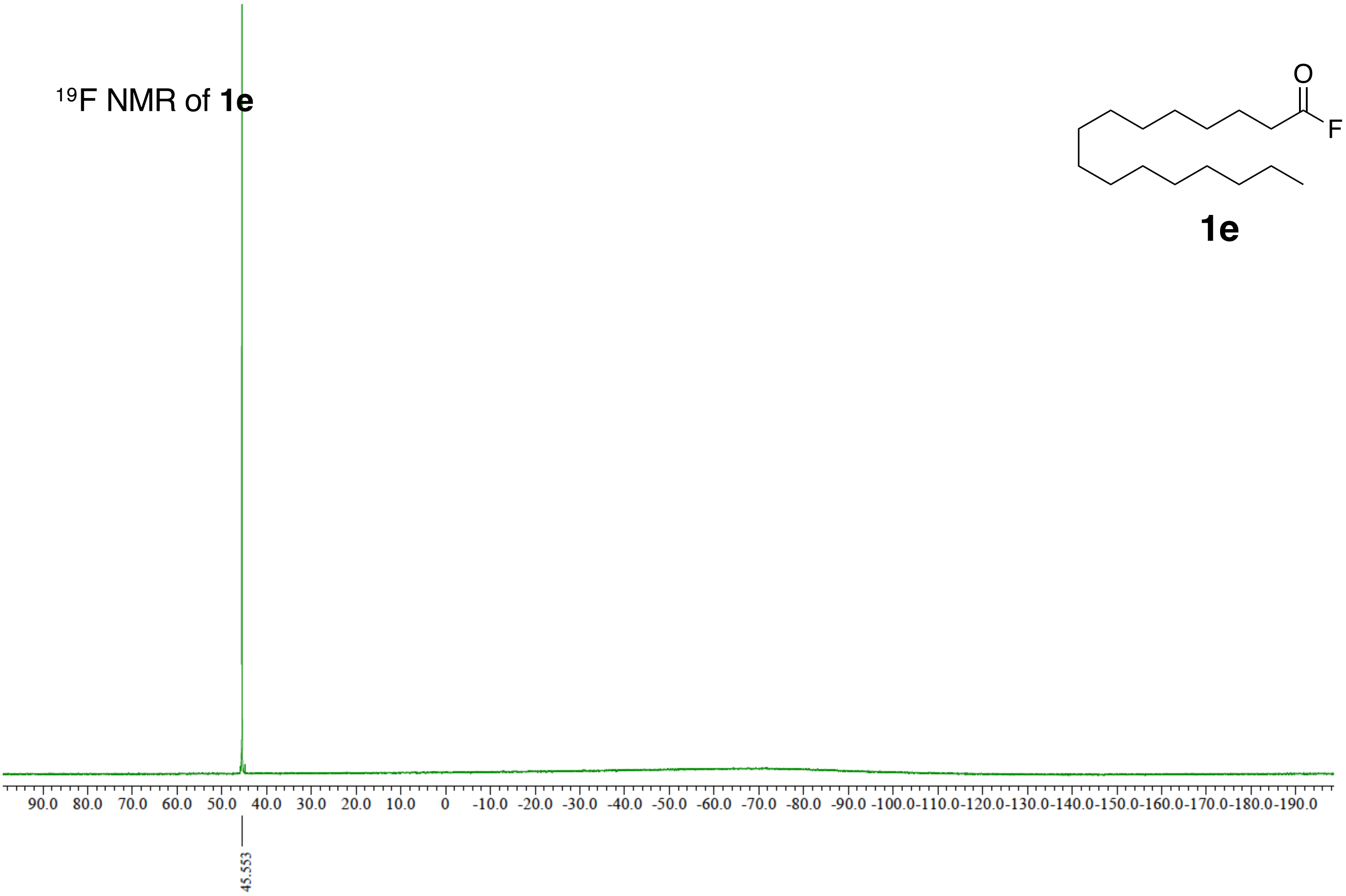

1e

^{19}F NMR of **1e**

^{1}H NMR of **1f**

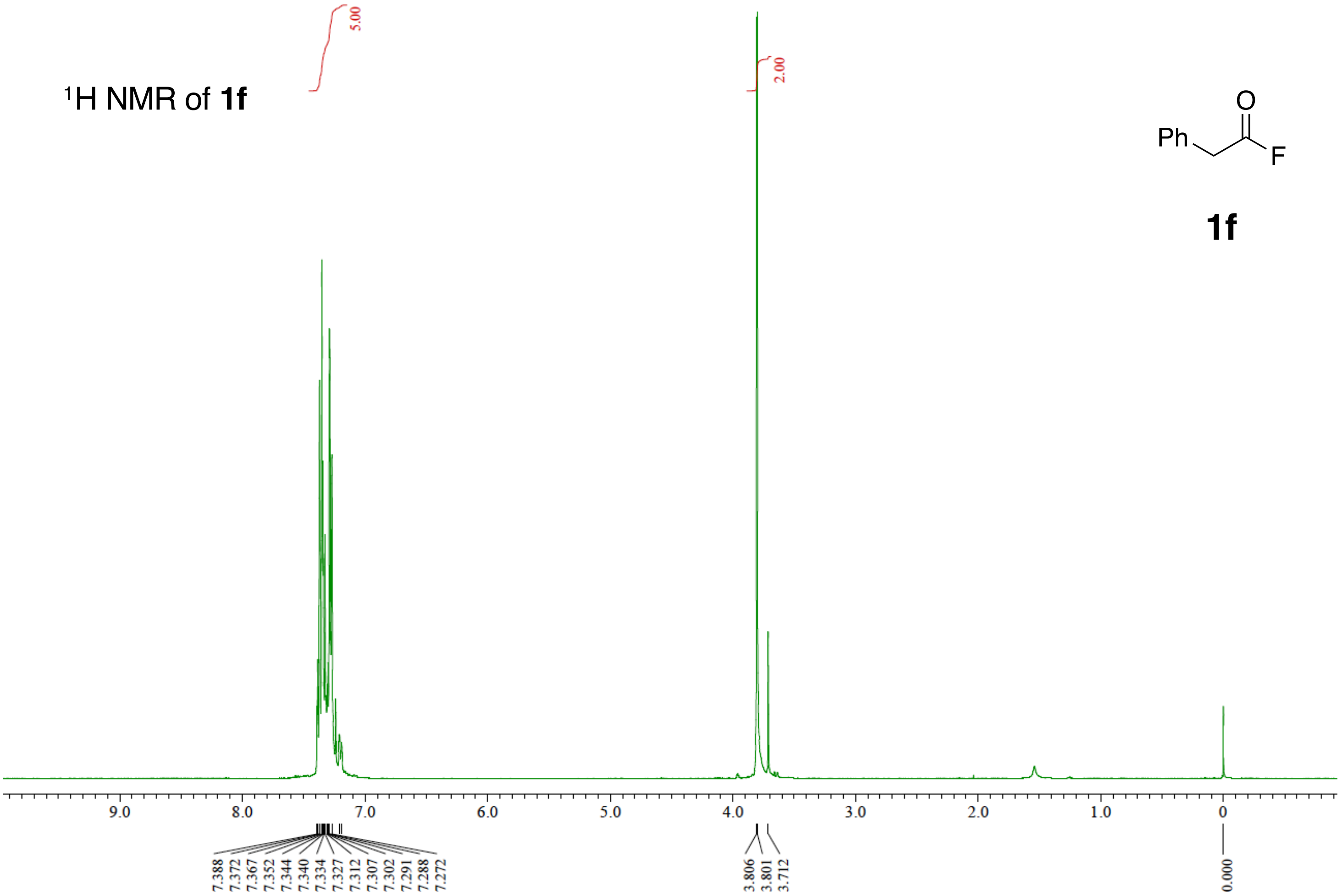

1f

^{13}C NMR of **1f**

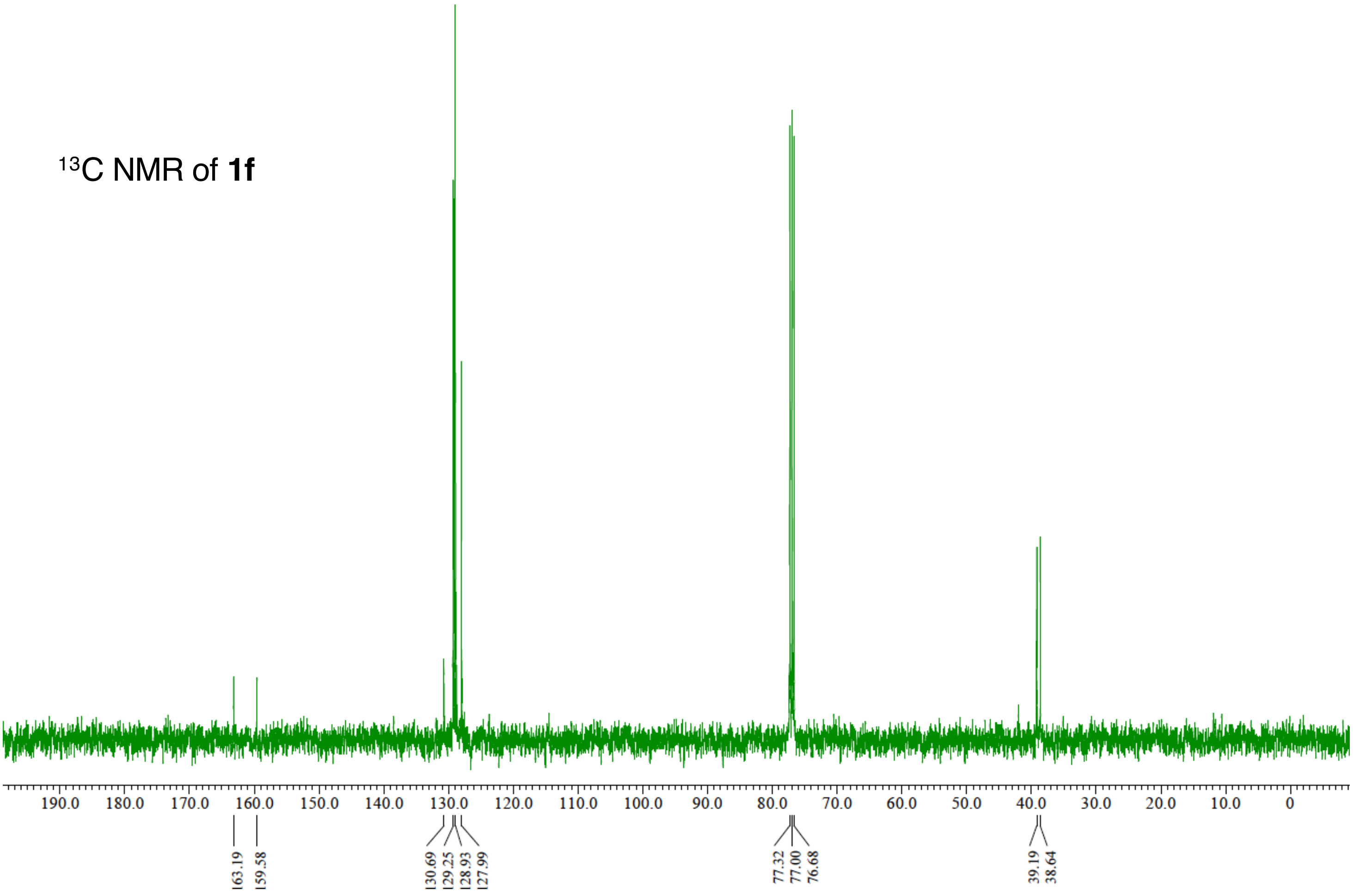

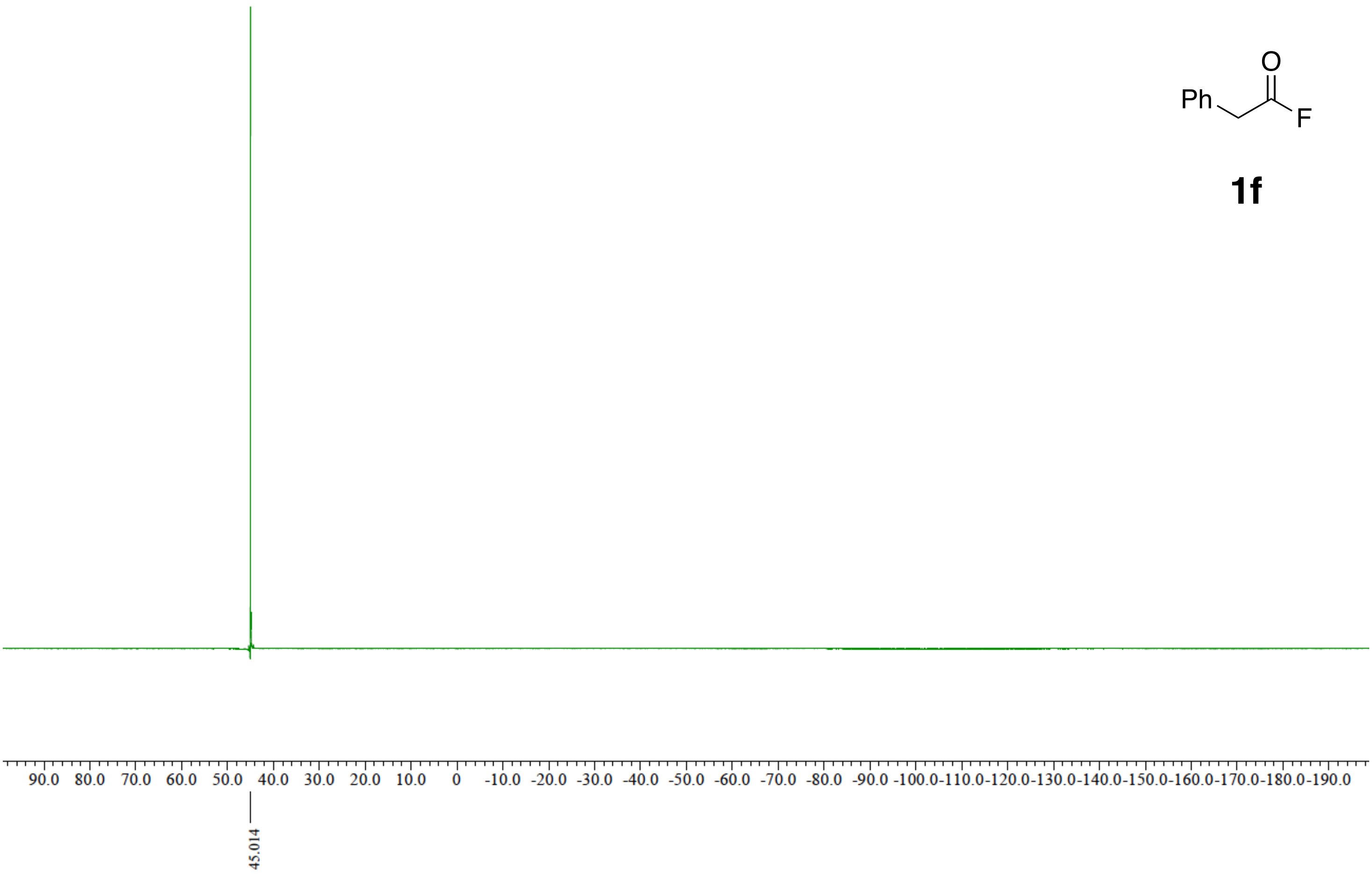

Ph
O
F
1f
90.0 80.0 70.0 60.0 50.0 40.0 30.0 20.0 10.0 0 -10.0 -20.0 -30.0 -40.0 -50.0 -60.0 -70.0 -80.0 -90.0 -100.0 -110.0 -120.0 -130.0 -140.0 -150.0 -160.0 -170.0 -180.0 -190.0
45.014

^{19}F NMR of **1f**

^{1}H NMR of **1g**

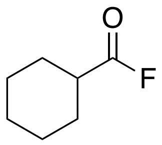

1g

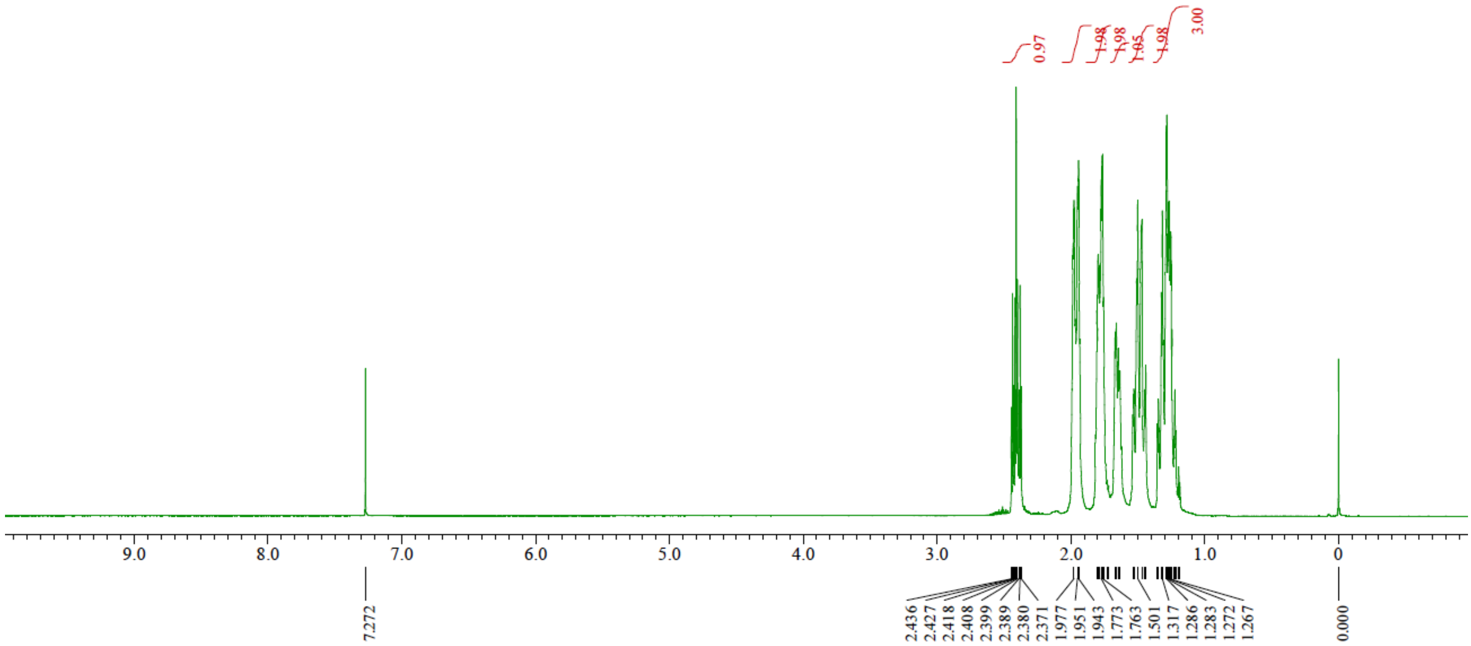

^{13}C NMR of **1g**

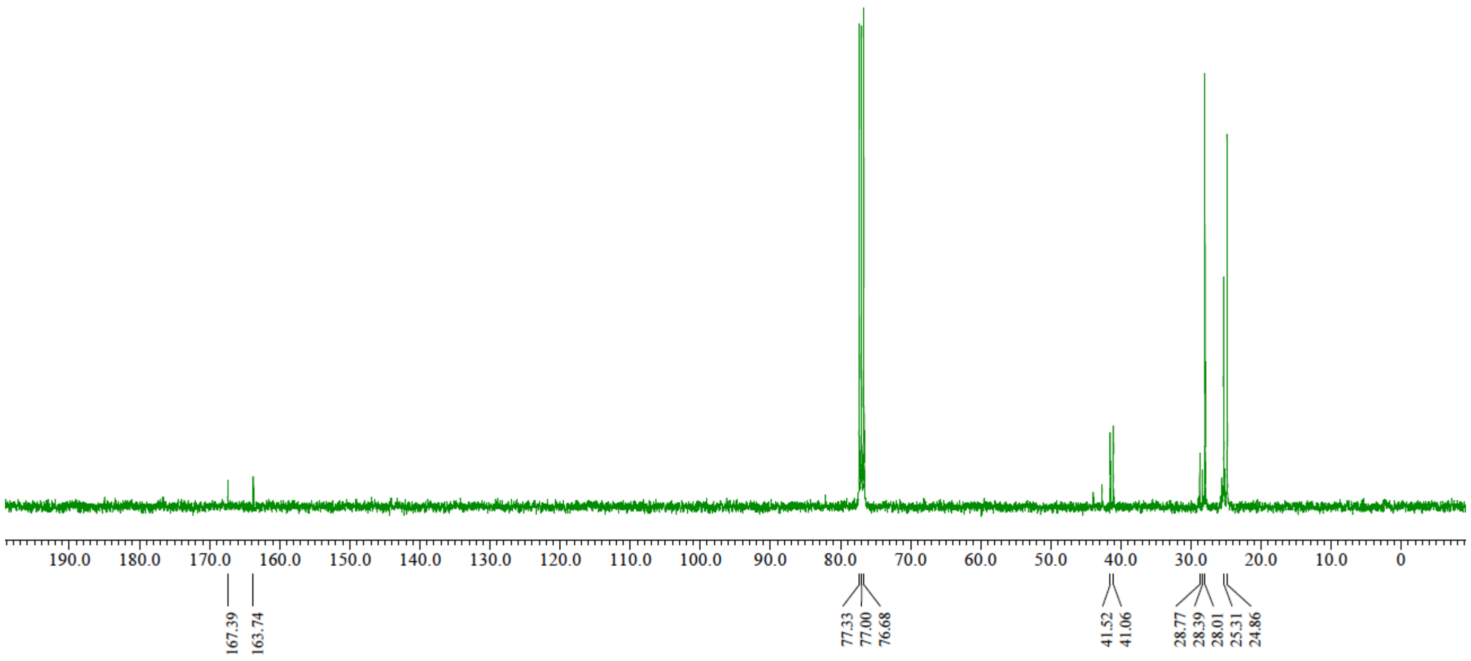

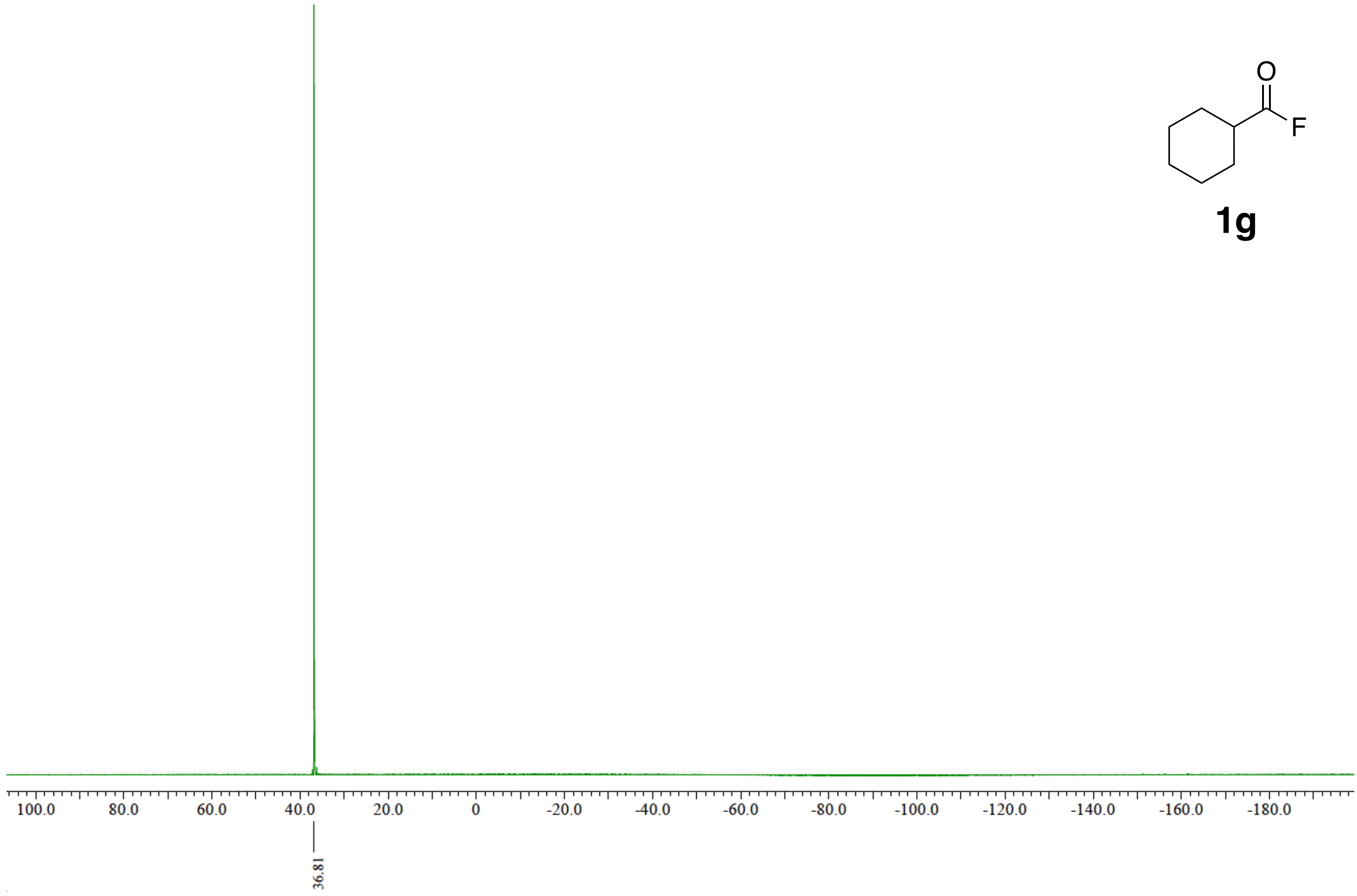

1g

^{19}F NMR of **1g**

^{1}H NMR of **1h**

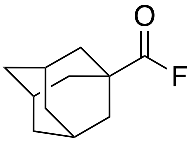

1h

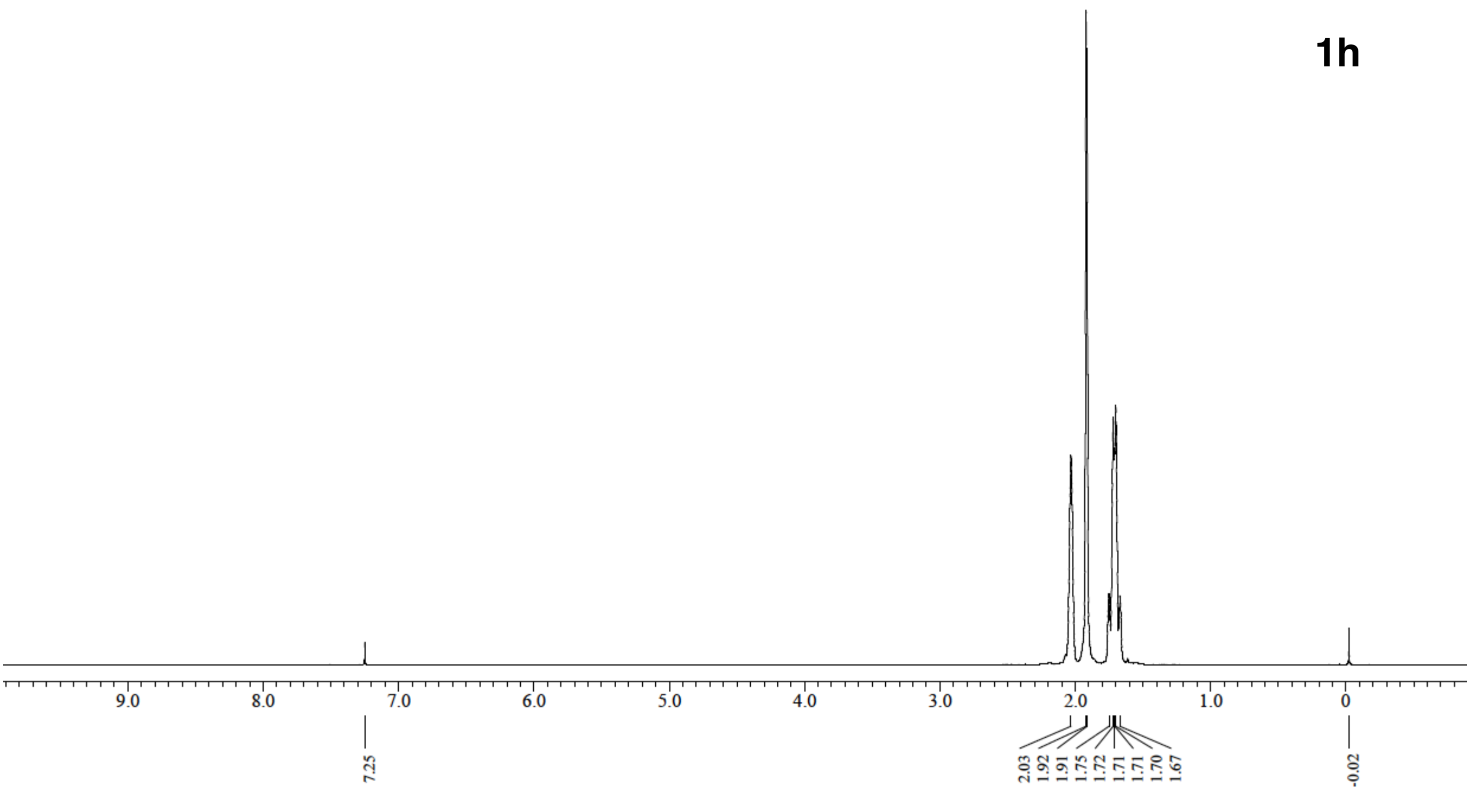

^{13}C NMR of **1h**

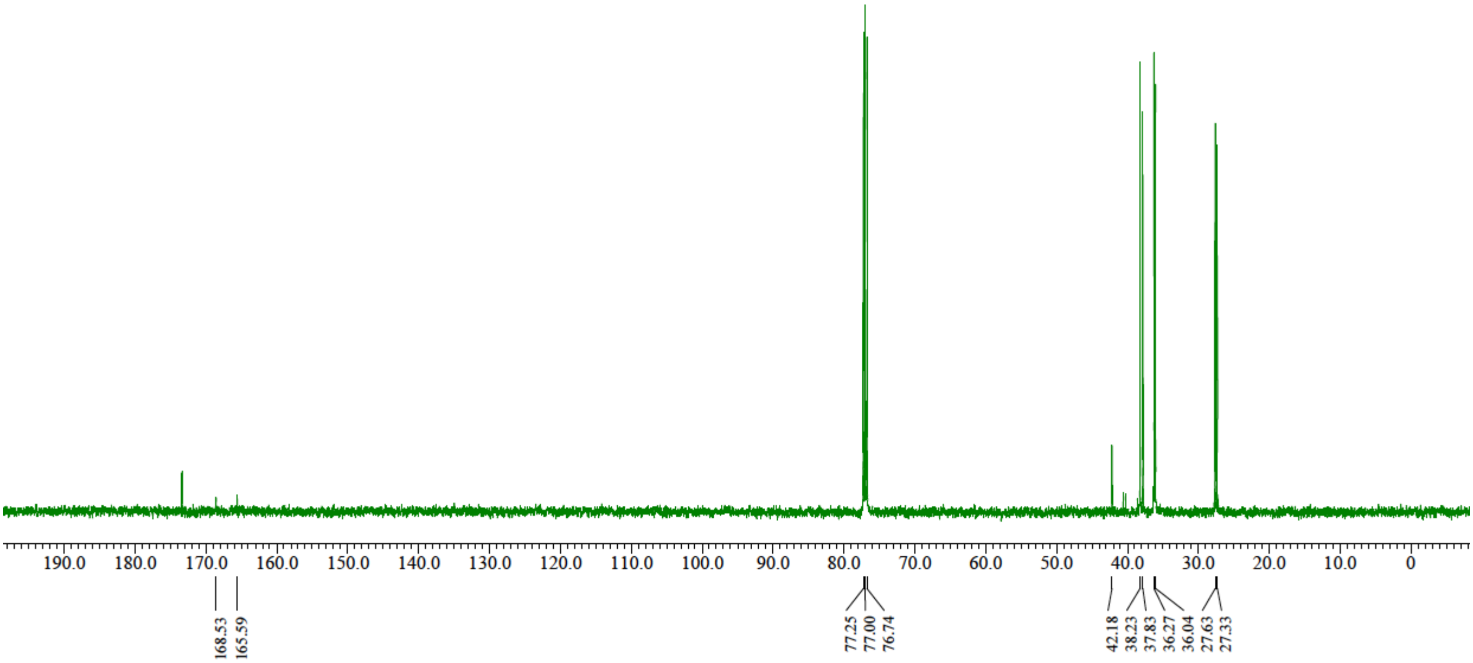

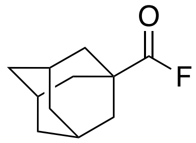

1h

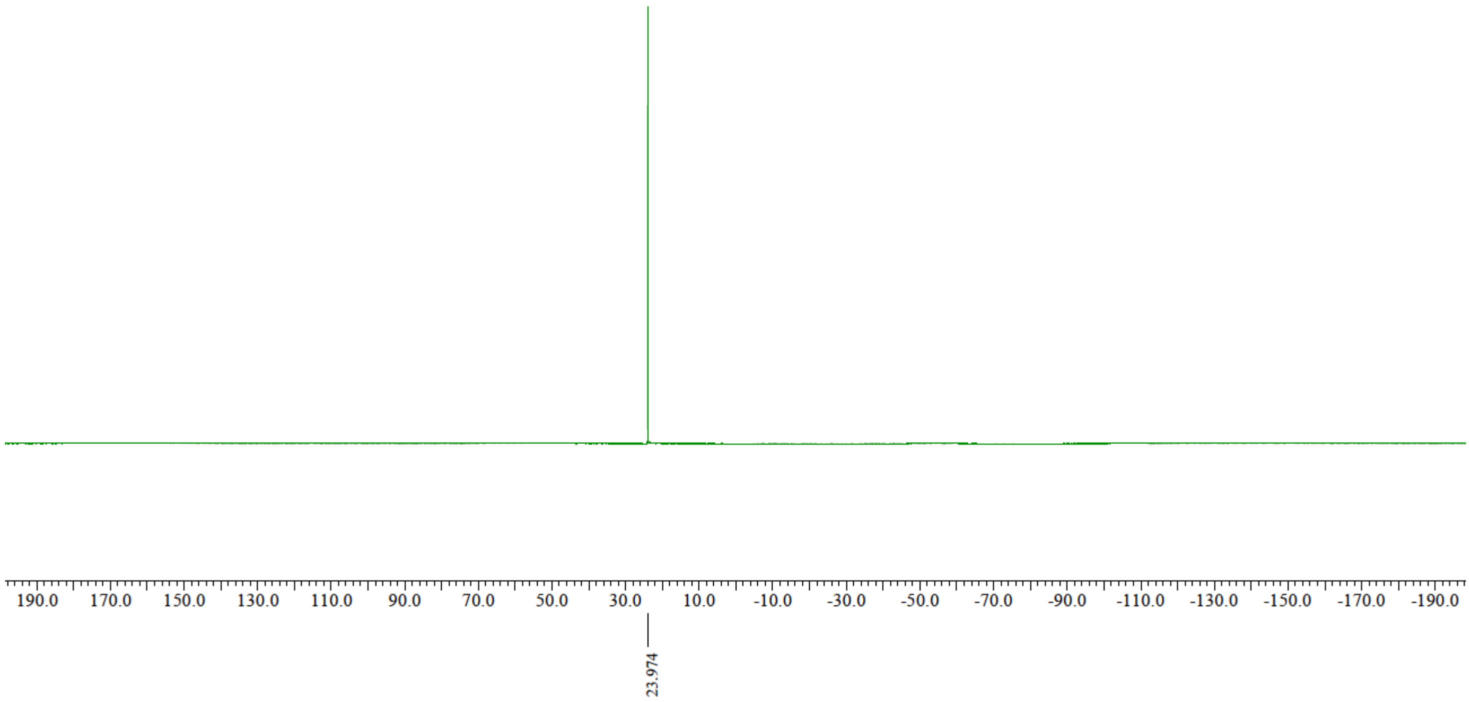

^{19}F NMR of **1h**

^{1}H NMR of **1i**

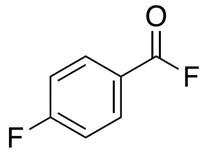

1i

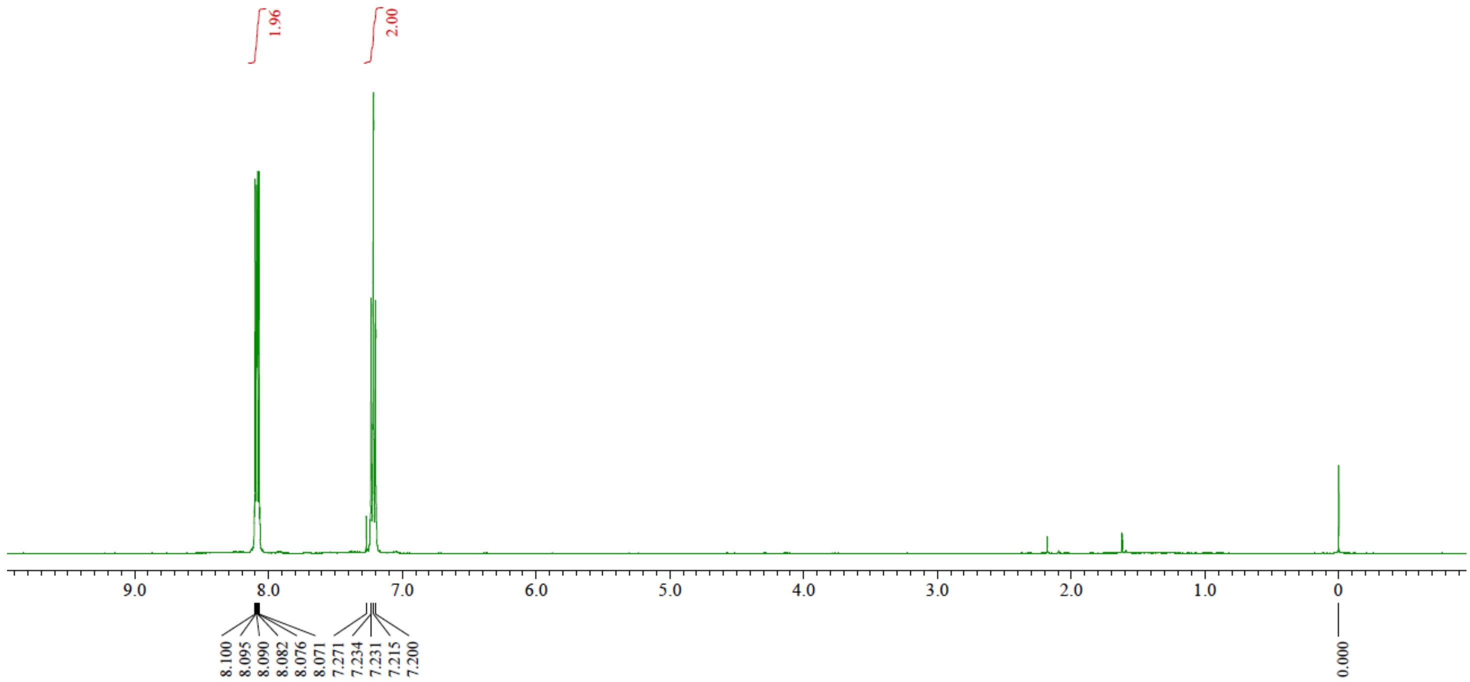

^{13}C NMR of **1i**

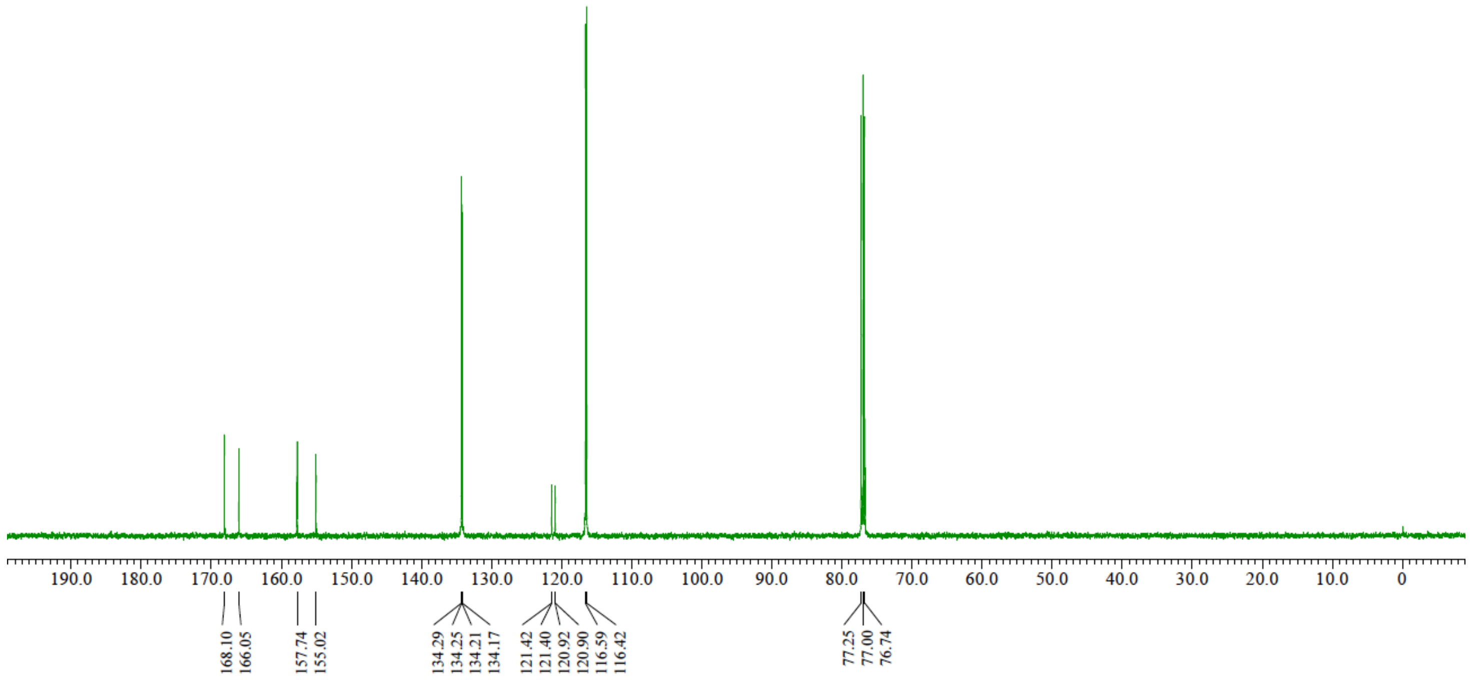

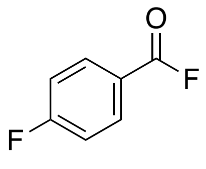

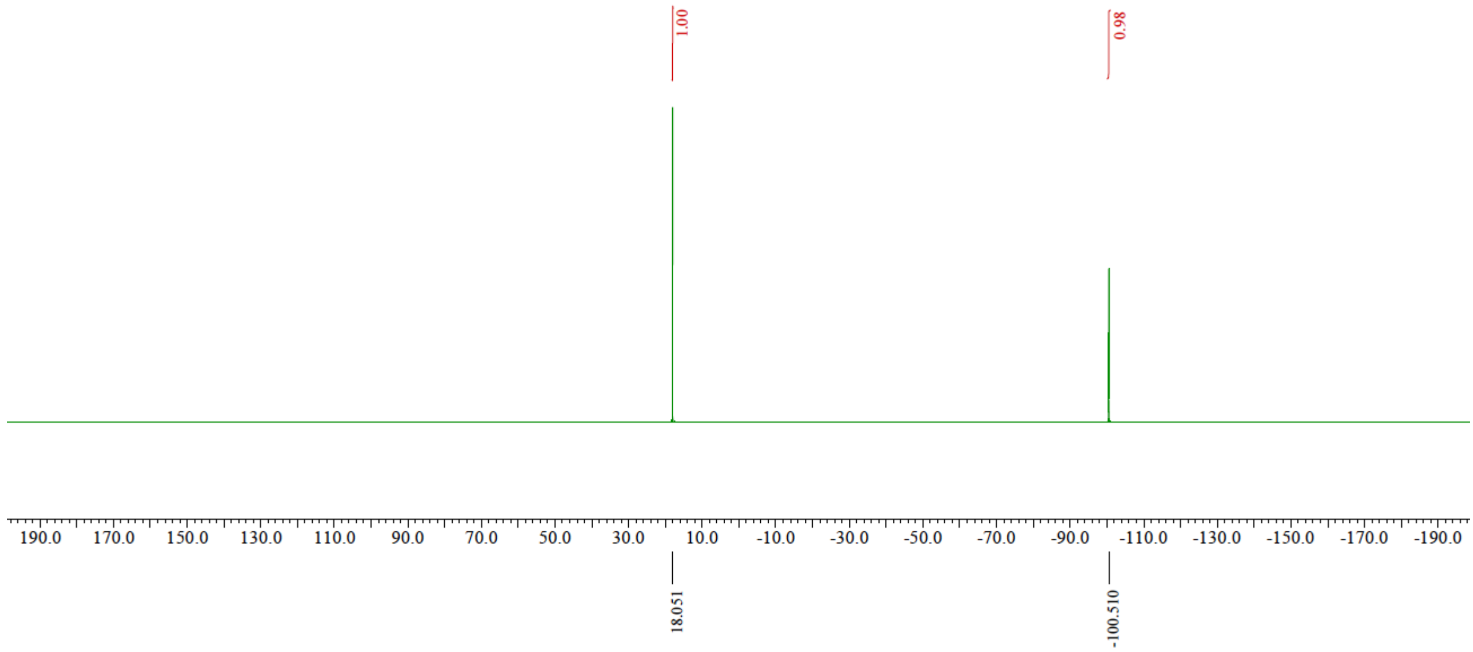

1.00
0.98
190.0
170.0
150.0
130.0
110.0
90.0
70.0
50.0
30.0
10.0
-10.0
-30.0
-50.0
-70.0
-90.0
-110.0
-130.0
-150.0
-170.0
-190.0
18.051
-100.510

^{19}F NMR of **1i**

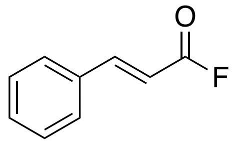

1j

^{1}H NMR of **1j**

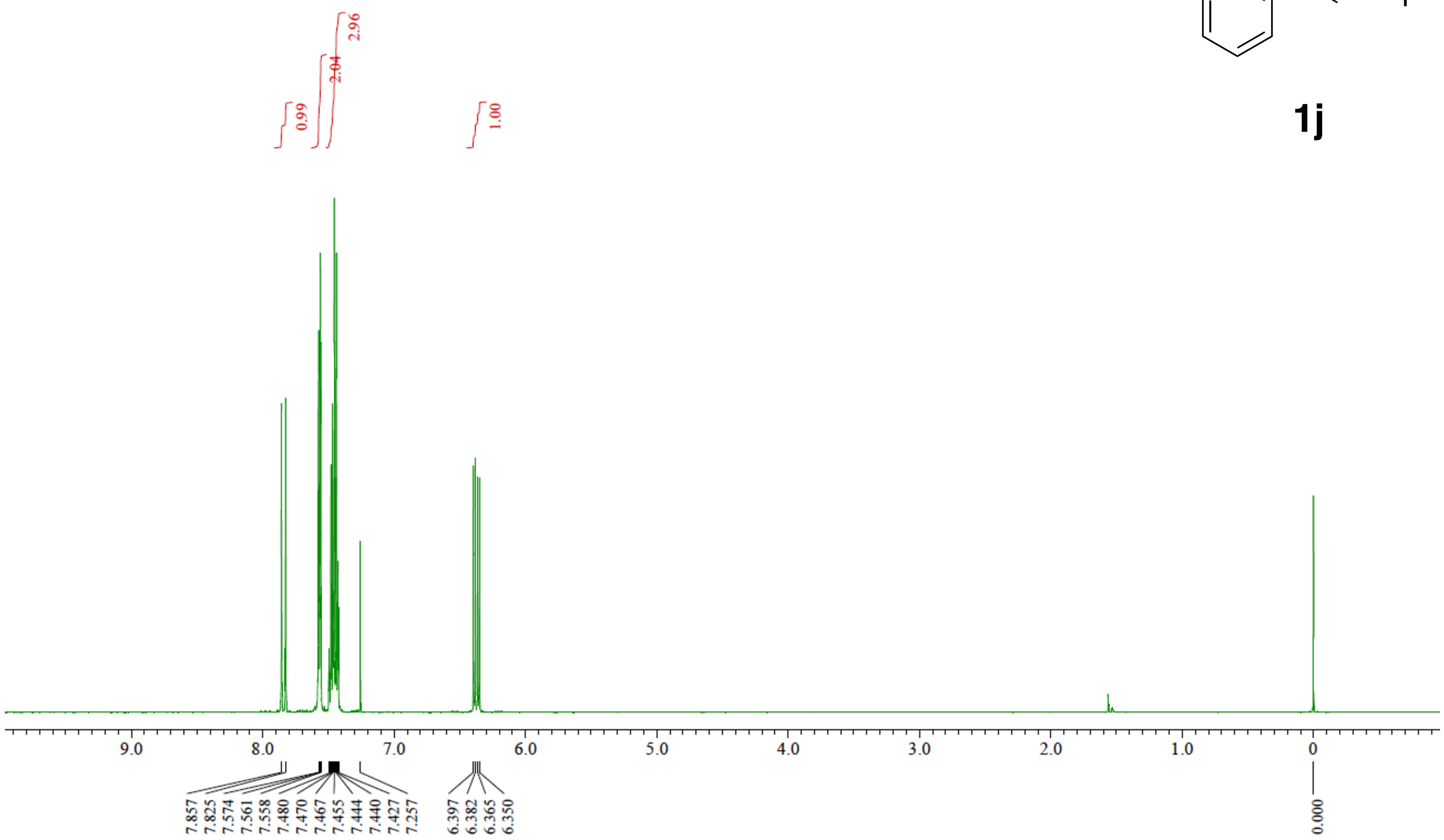

^{13}C NMR of **1j**

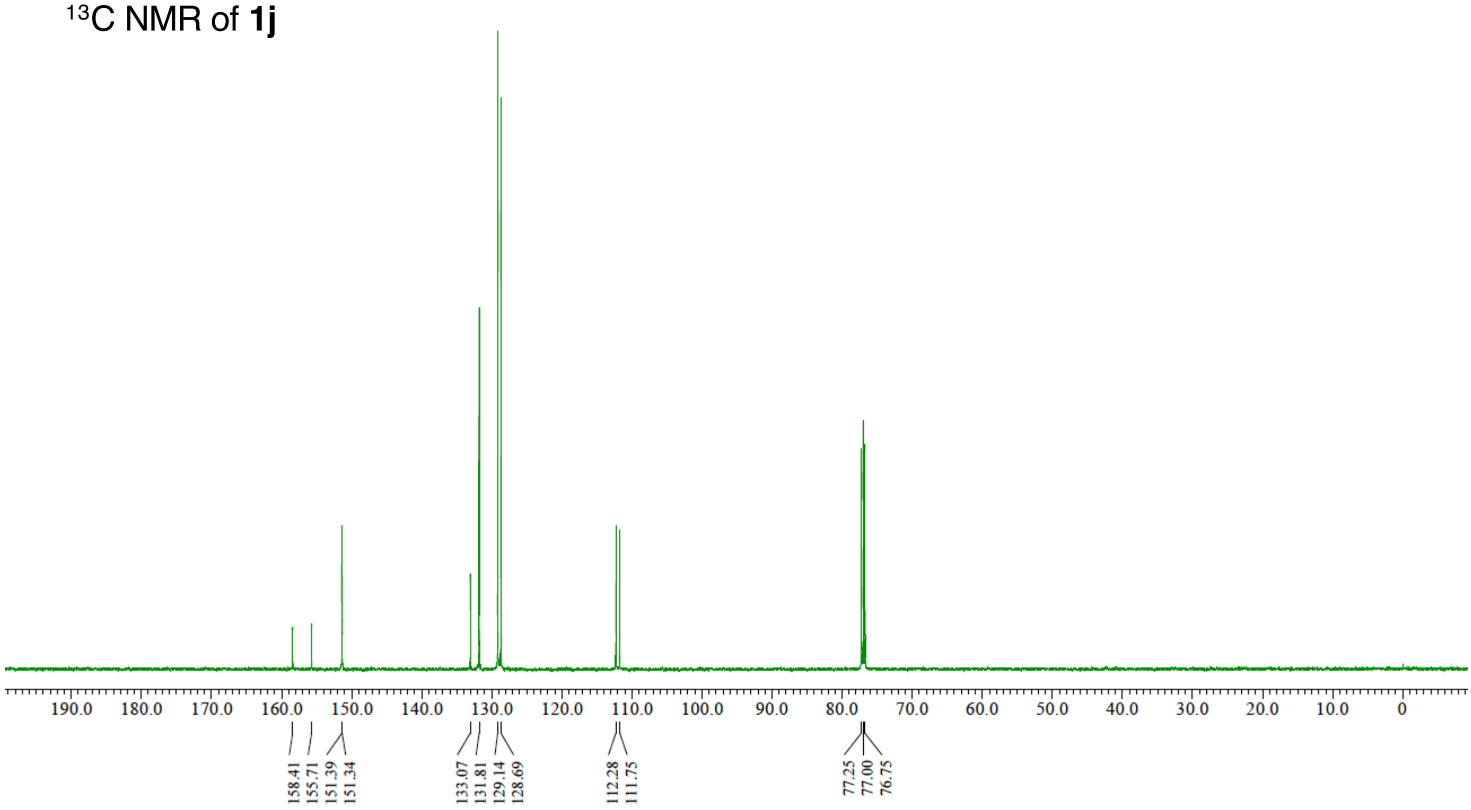

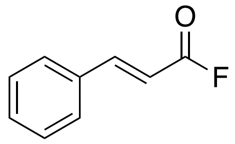

1j

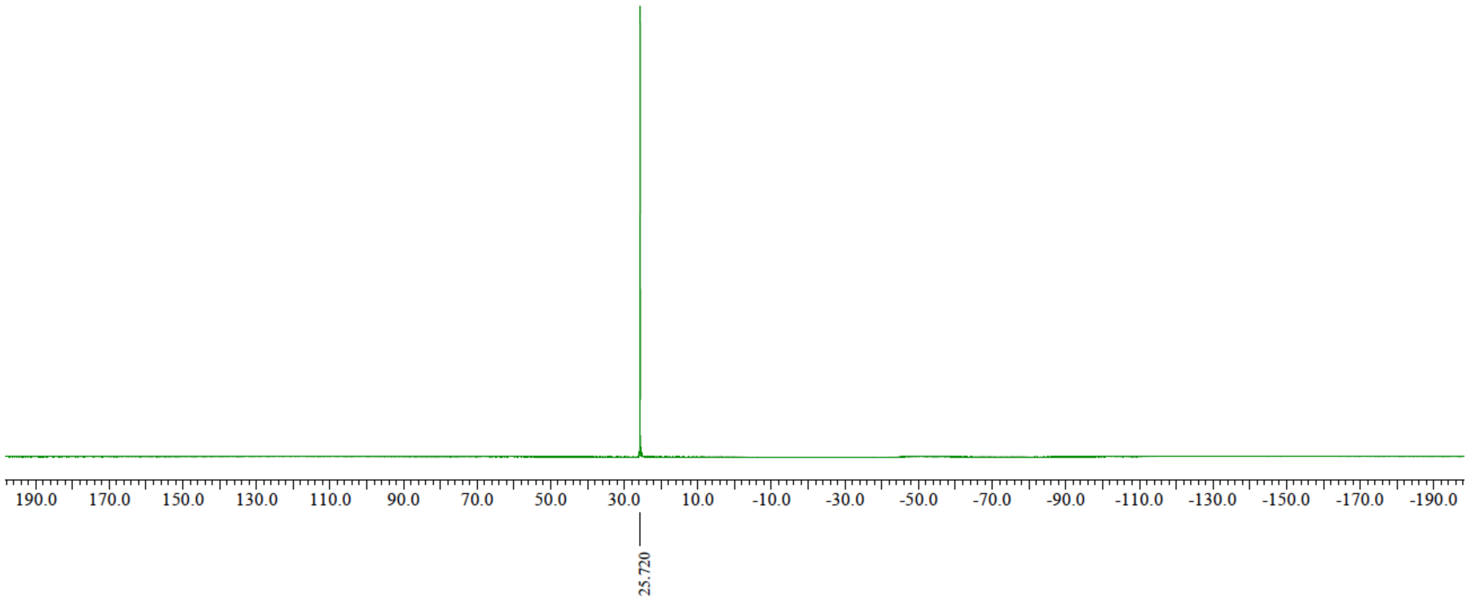

^{19}F NMR of **1j**

^1H NMR of **1k**

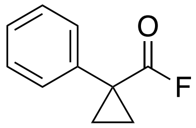

1k

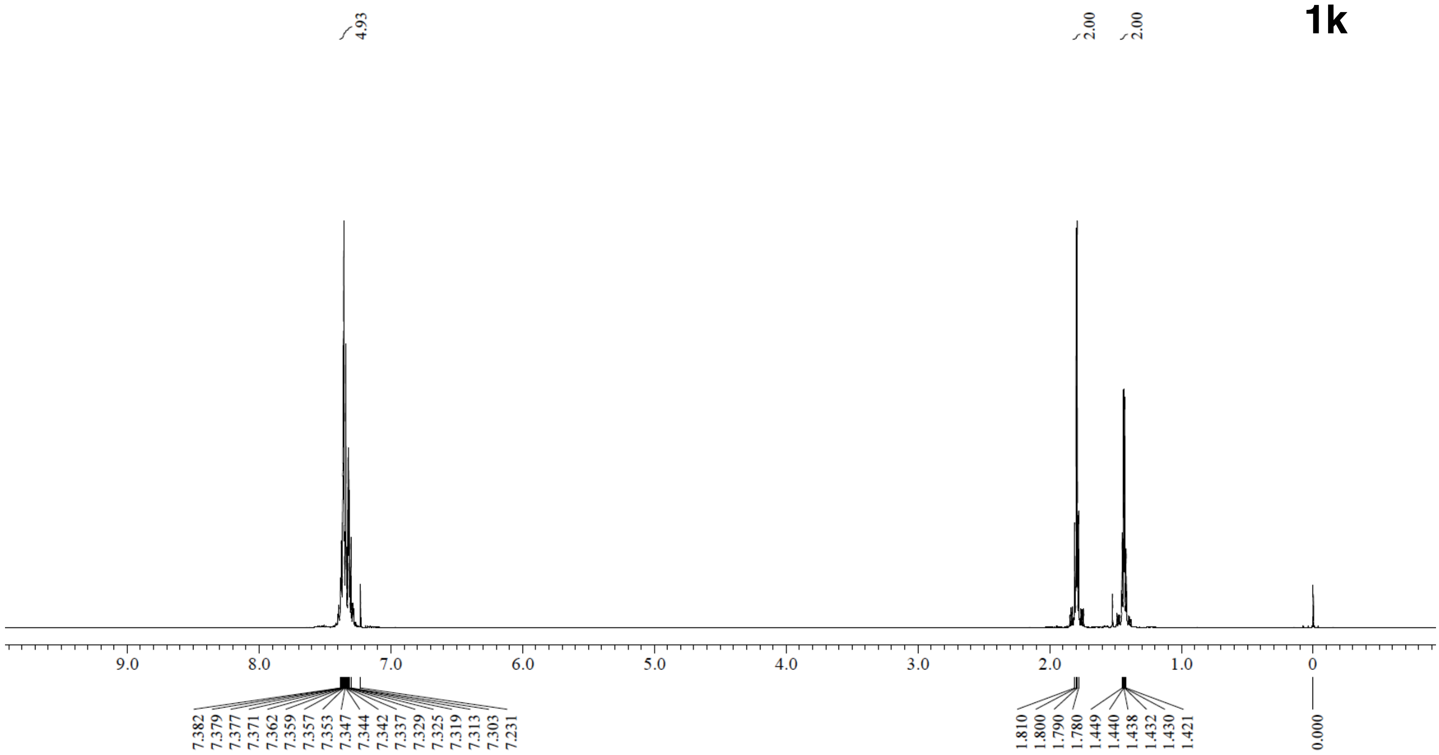

^{13}C NMR of **1k**

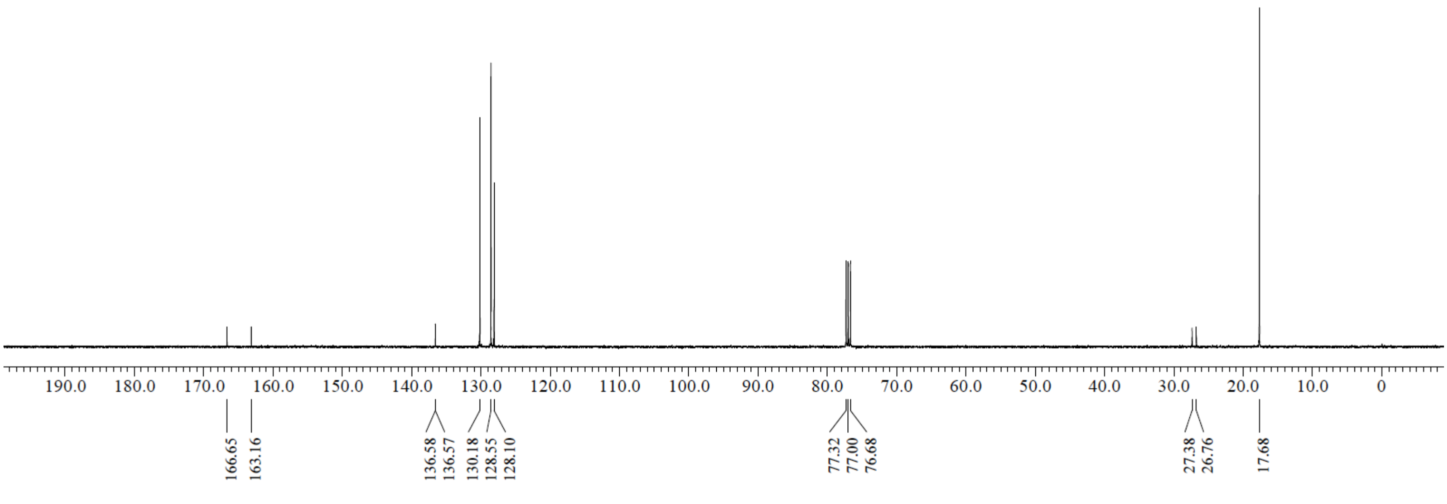

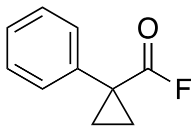

1k

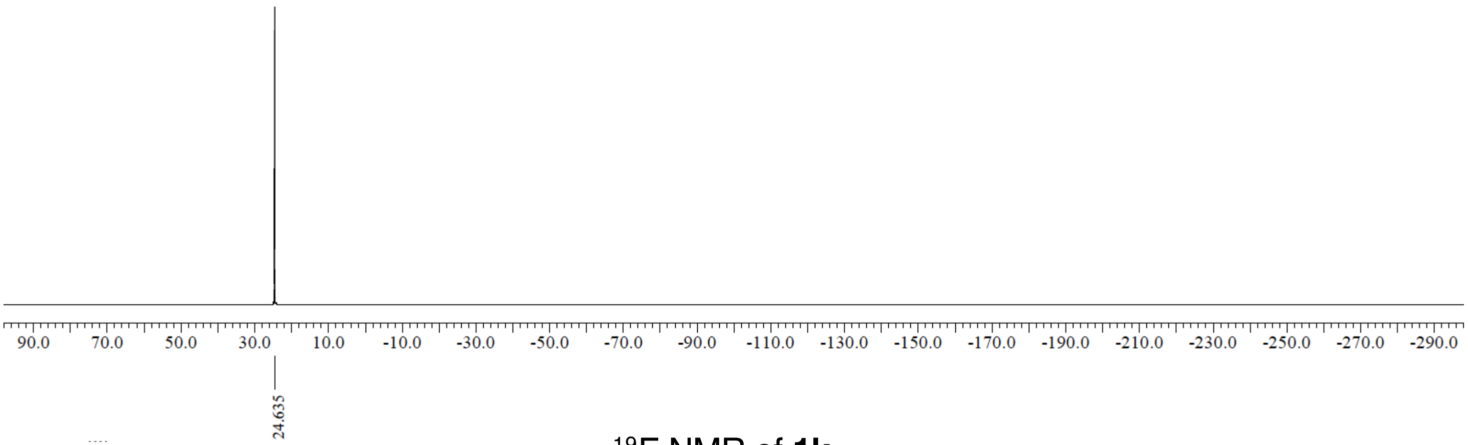

^{19}F NMR of **1k**

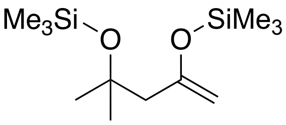

2i

^{1}H NMR of **2i**

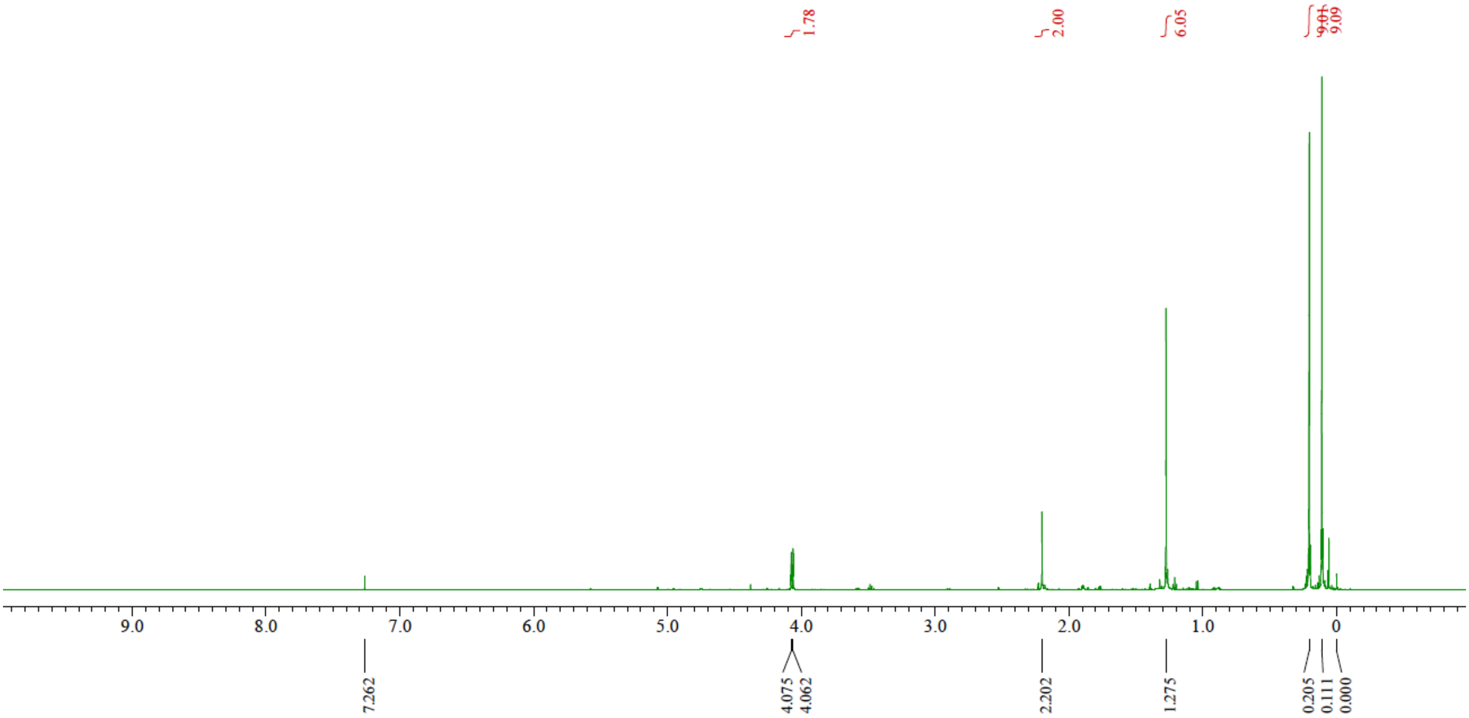

^{13}C NMR of **2i**

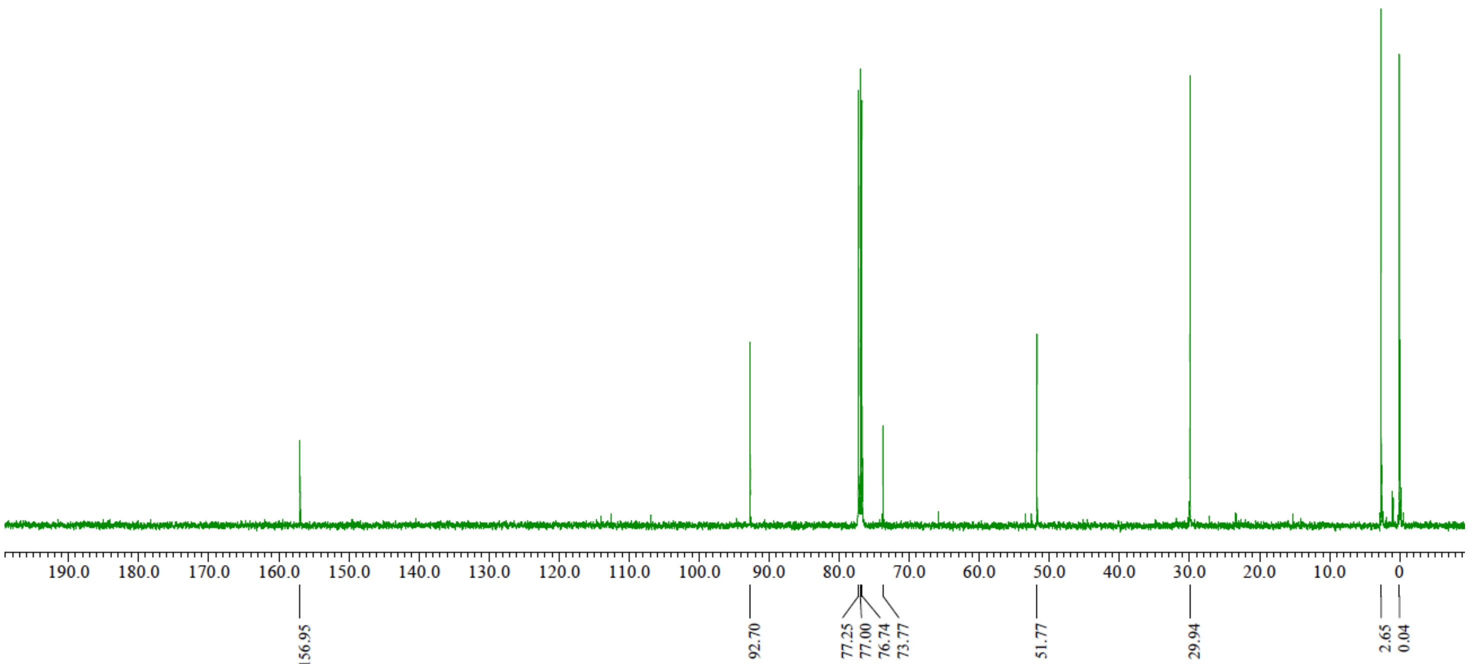

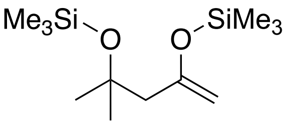

2i

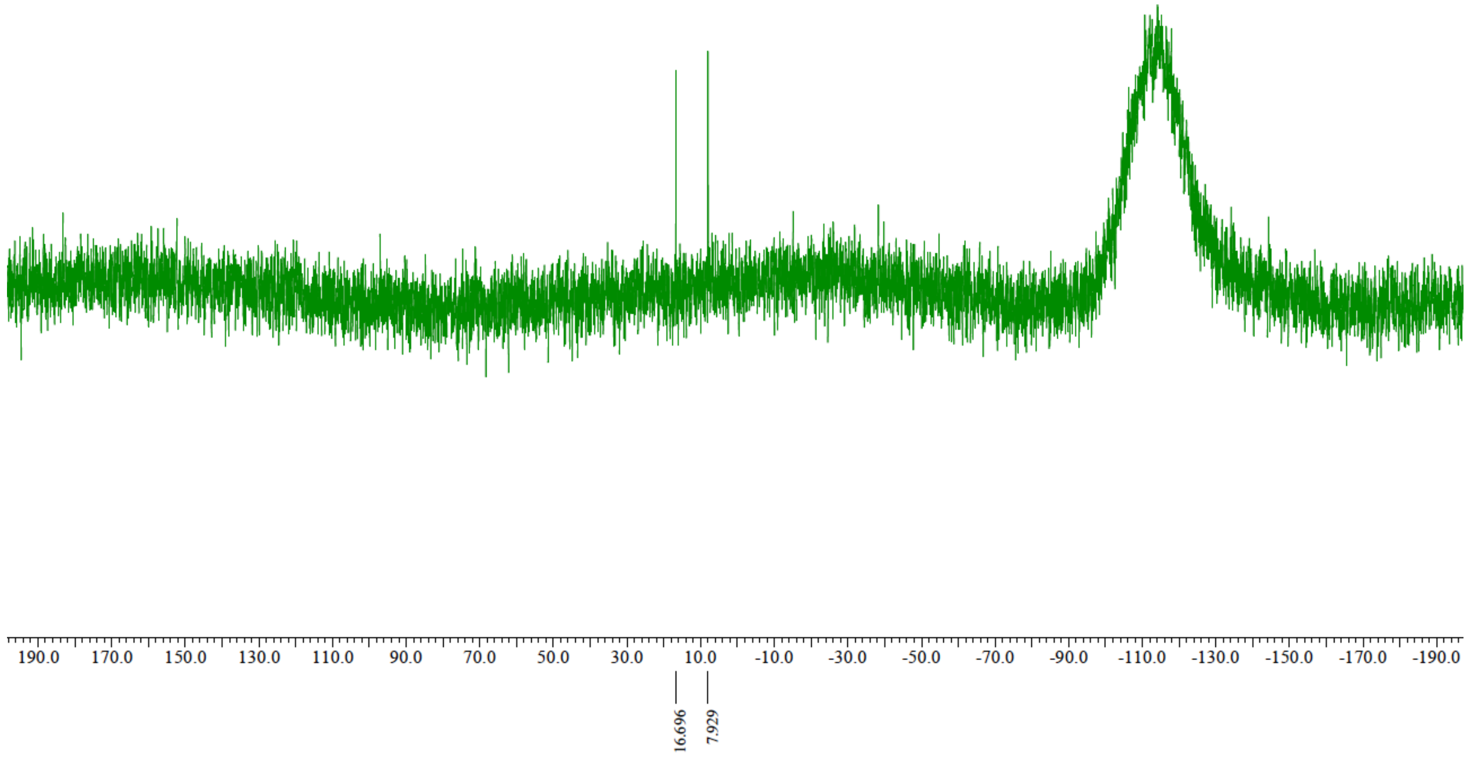

^{29}Si NMR of **2i**

^{1}H NMR of **3**

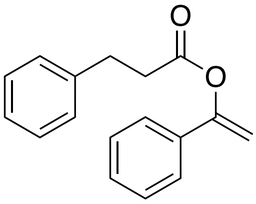

3

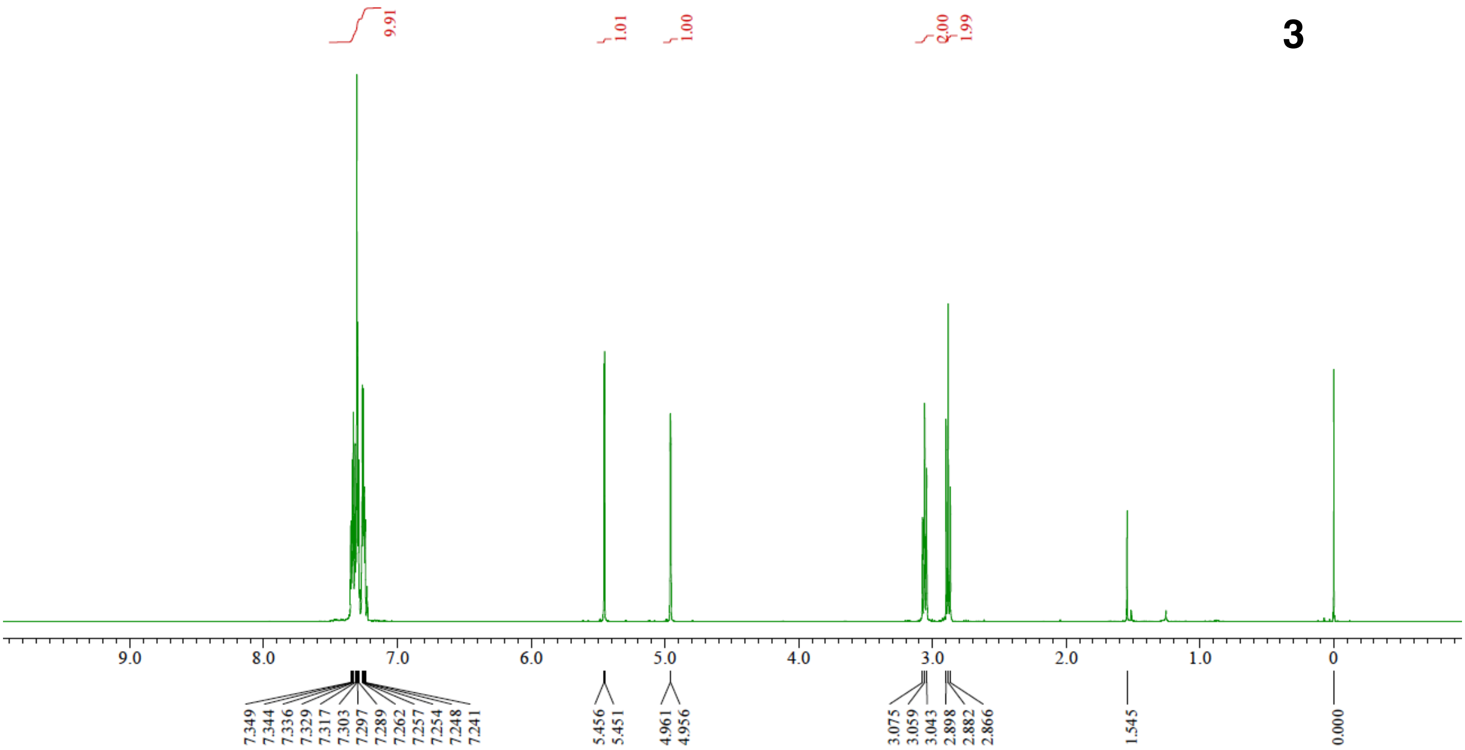

^{13}C NMR of **3**

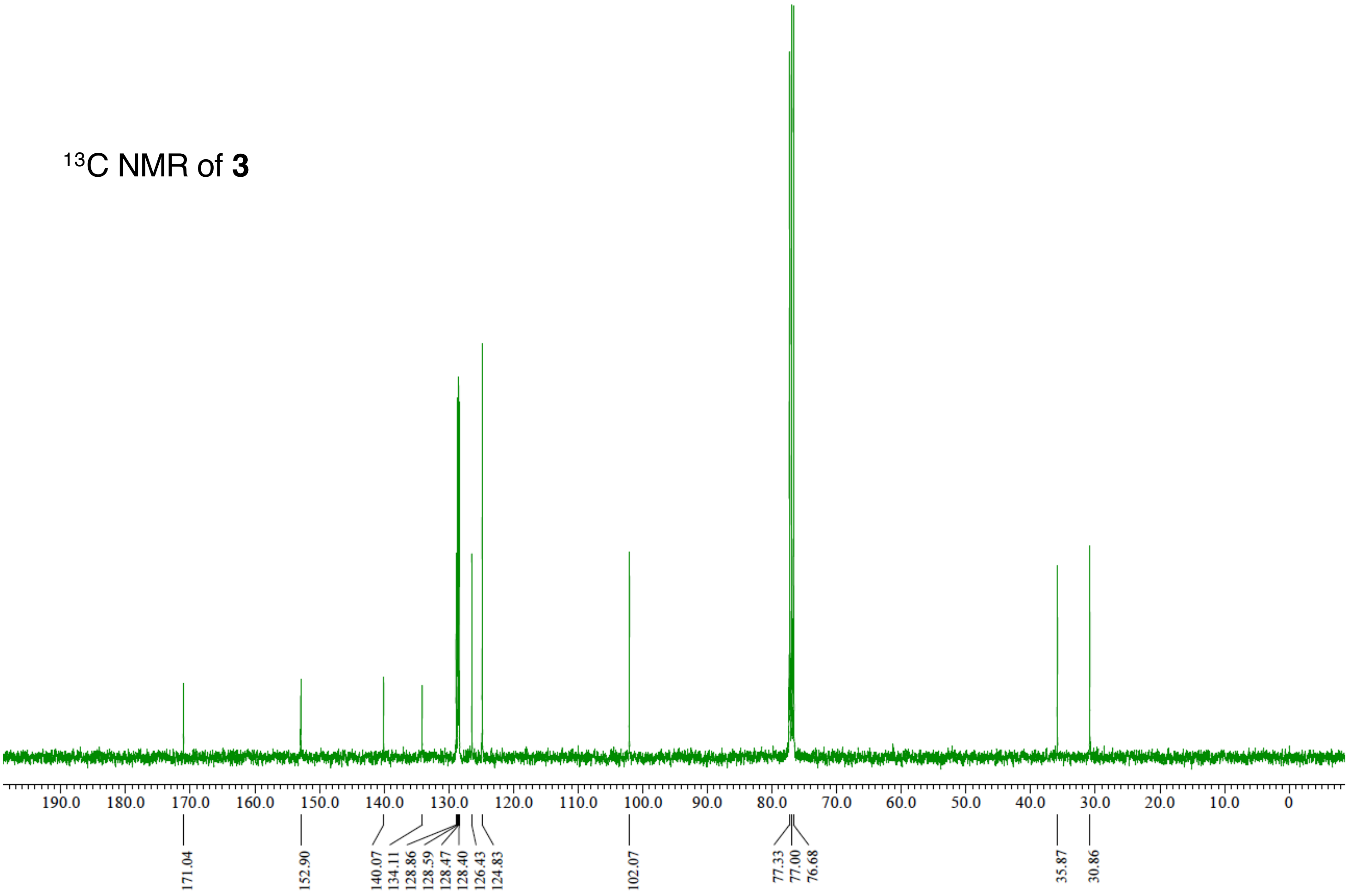

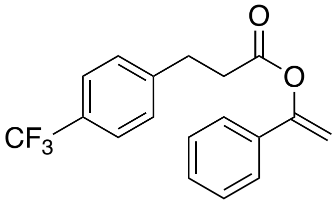

4

^{1}H NMR of **4**

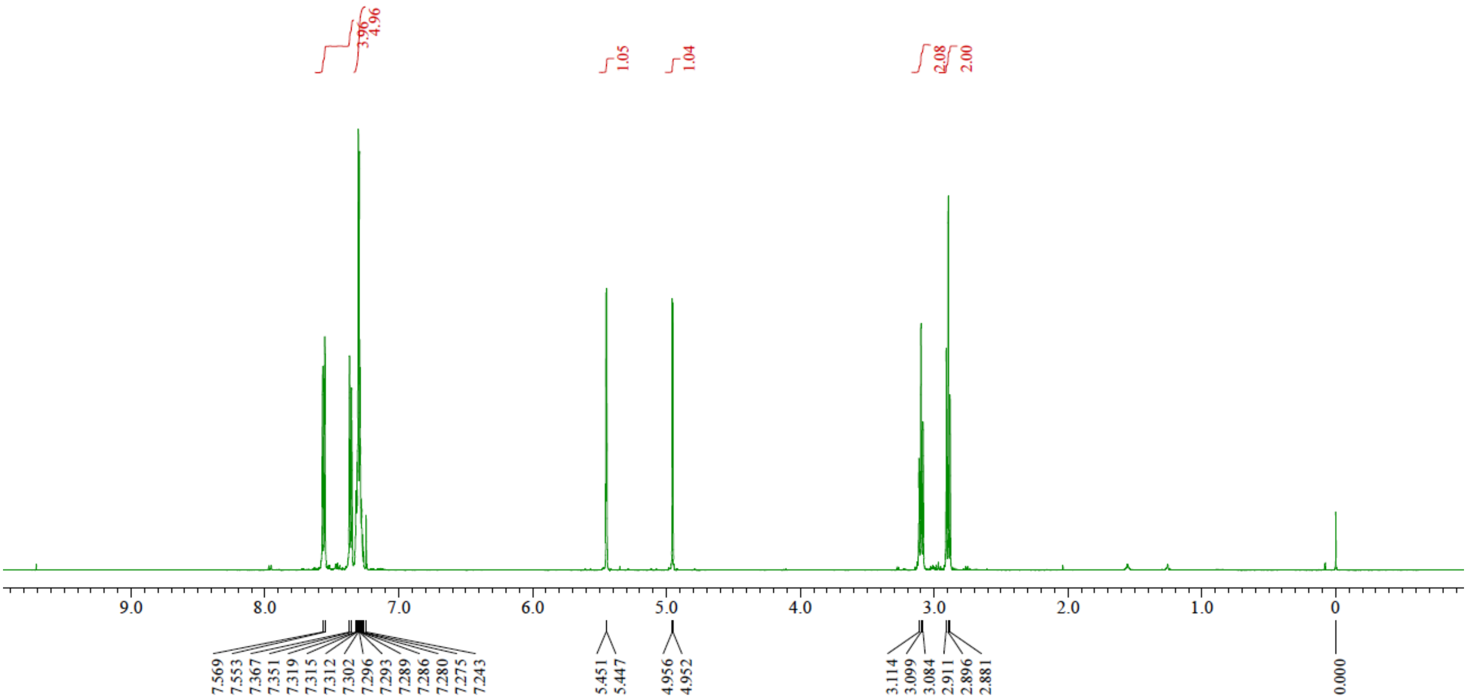

^{13}C NMR of **4**

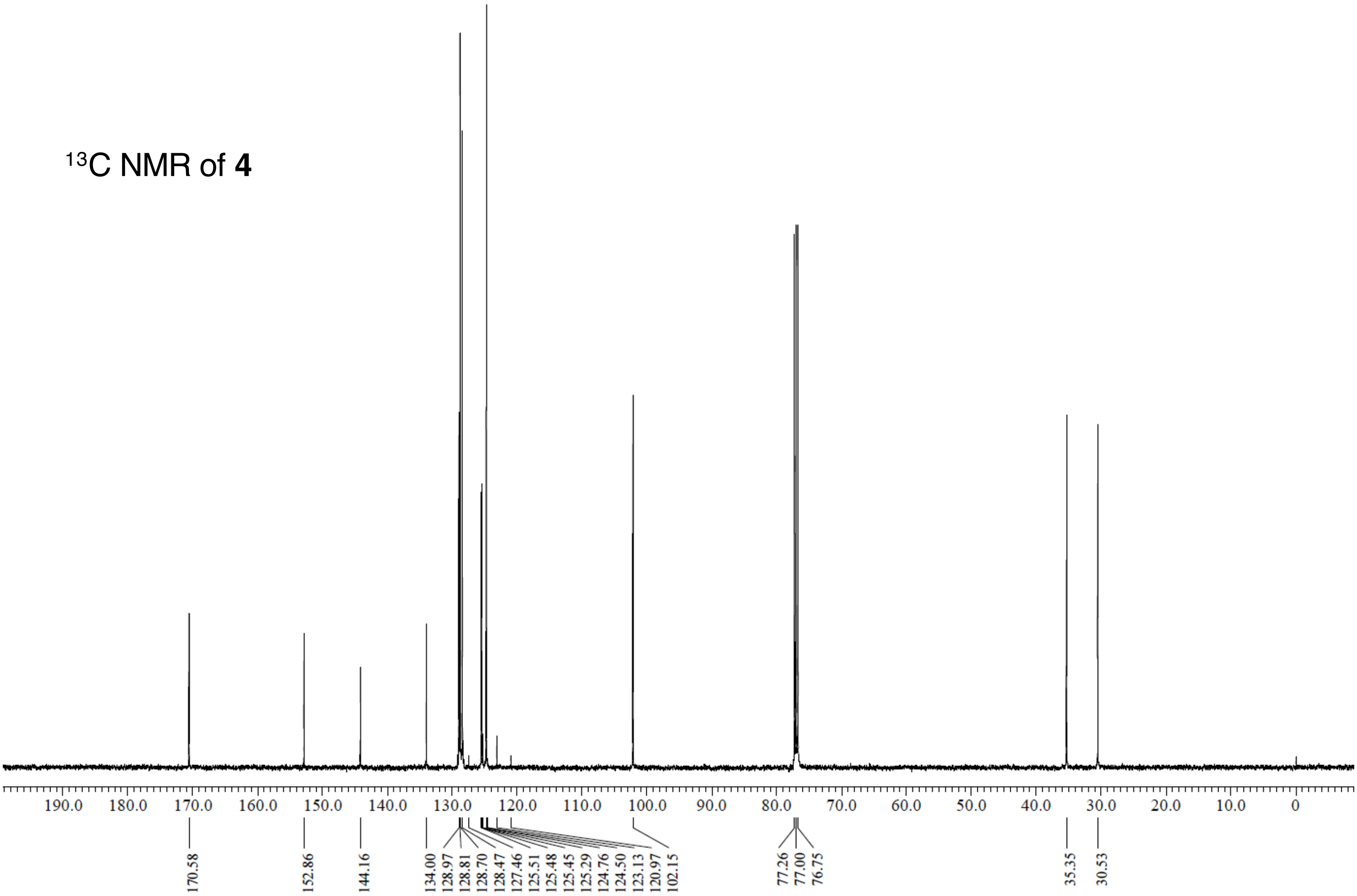

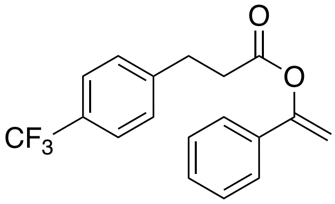
O
O
CF3

4

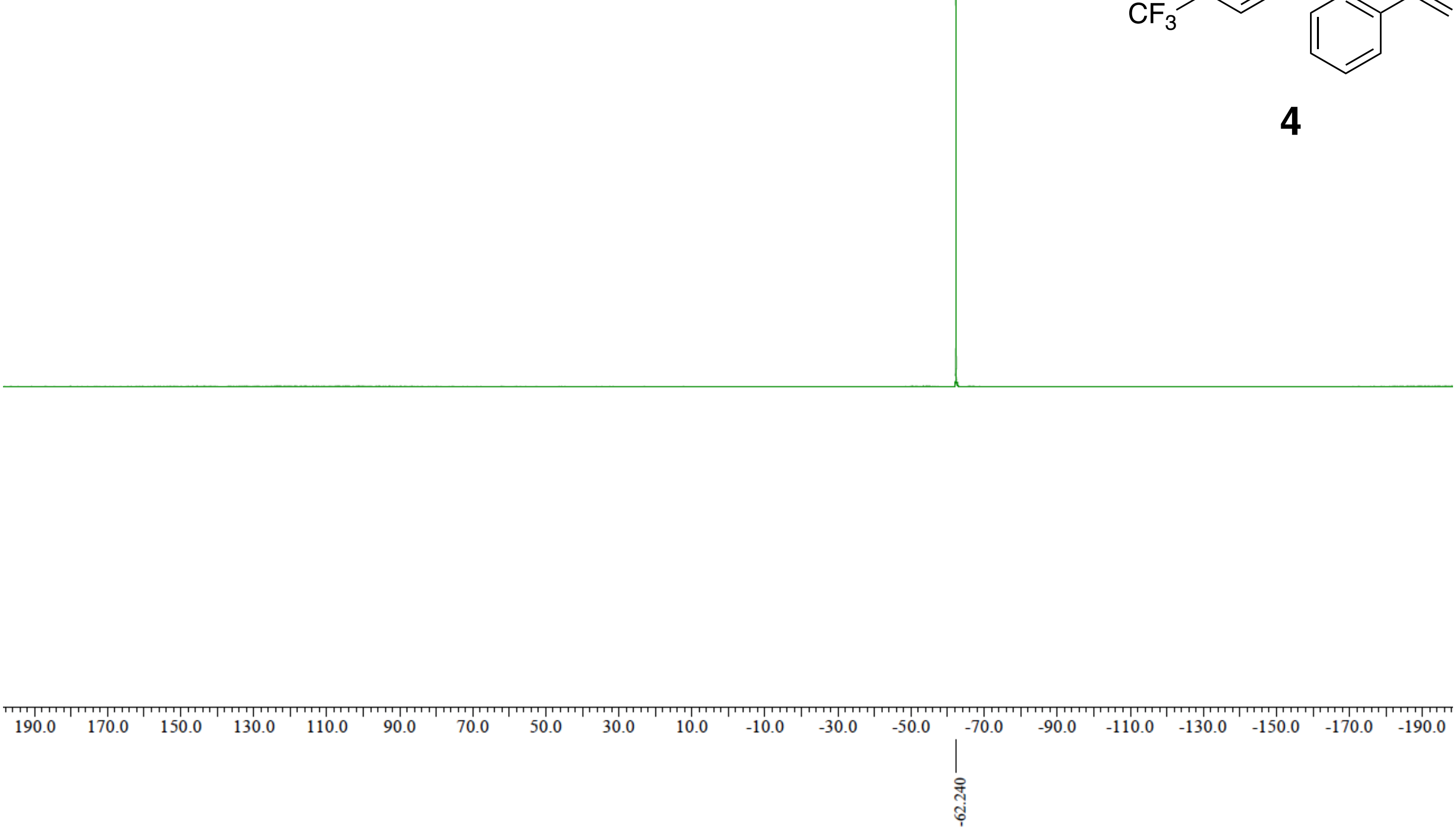
190.0 170.0 150.0 130.0 110.0 90.0 70.0 50.0 30.0 10.0 -10.0 -30.0 -50.0 -70.0 -90.0 -110.0 -130.0 -150.0 -170.0 -190.0
-62.240

^{19}F NMR of **4**

^{1}H NMR of **6**

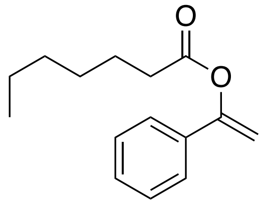

6

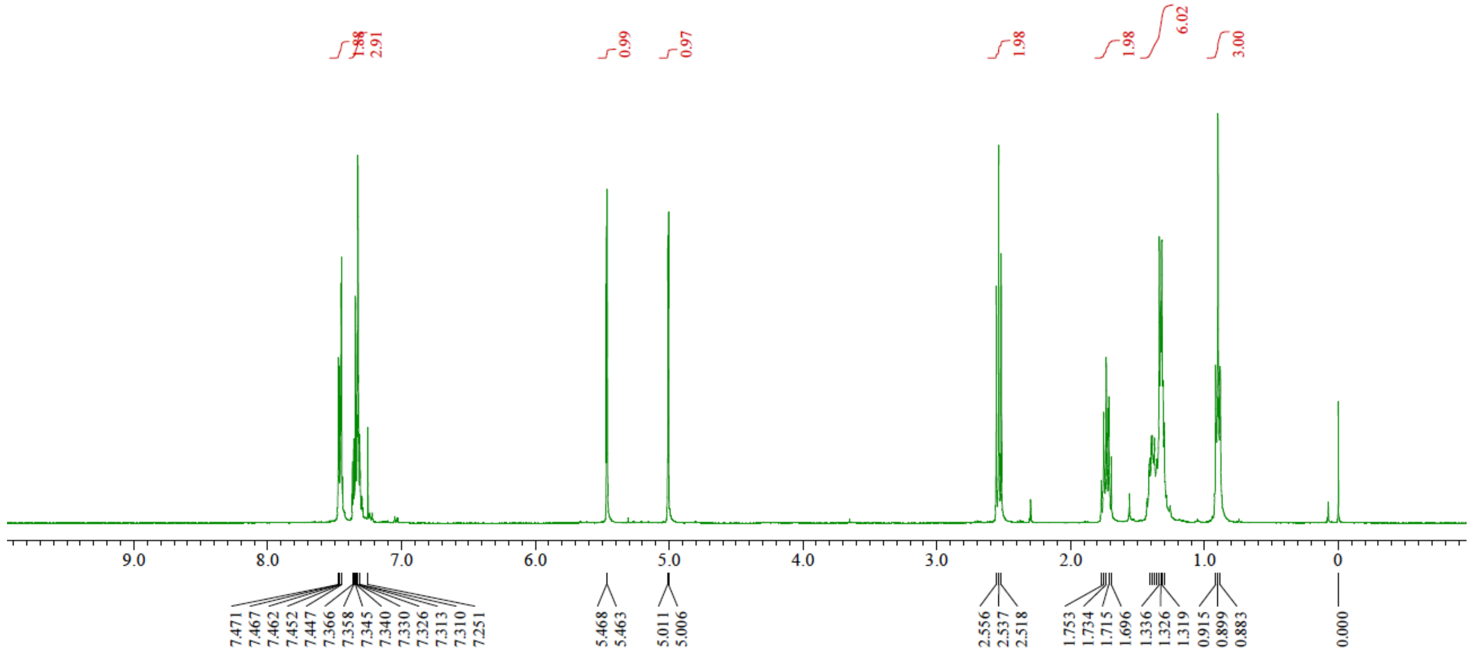

^{13}C NMR of **6**

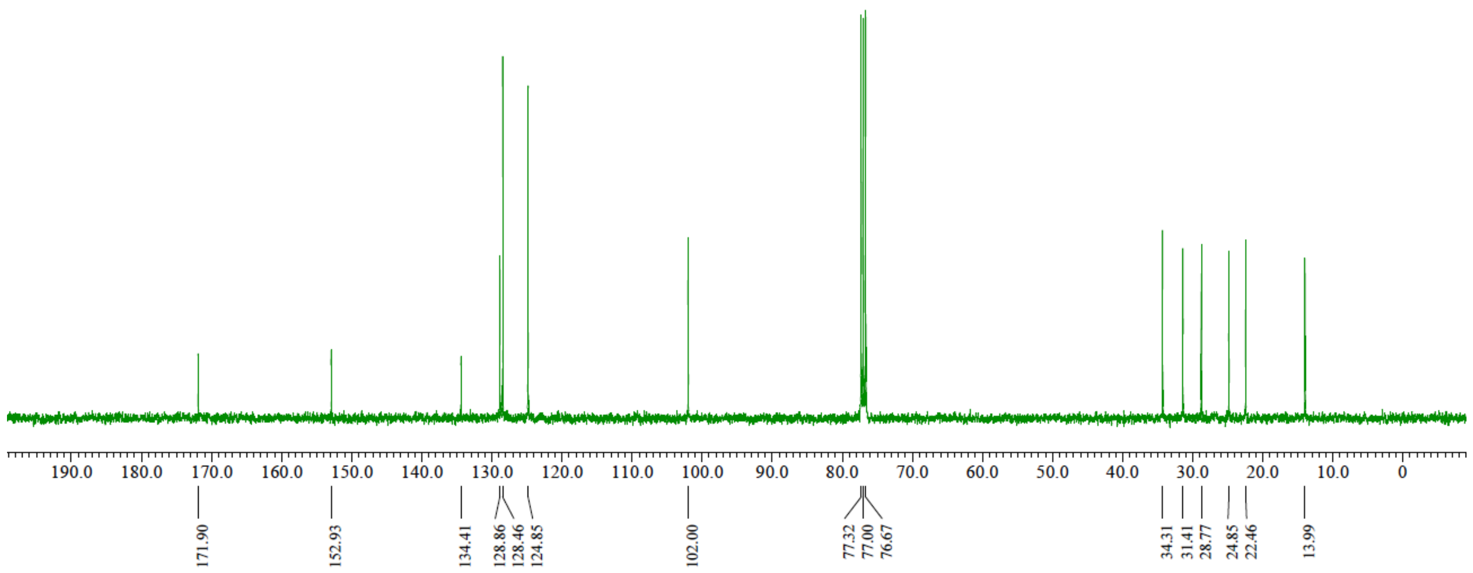

^{1}H NMR of **7**

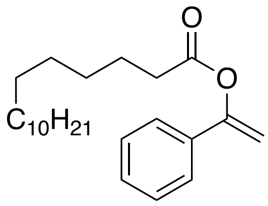

7

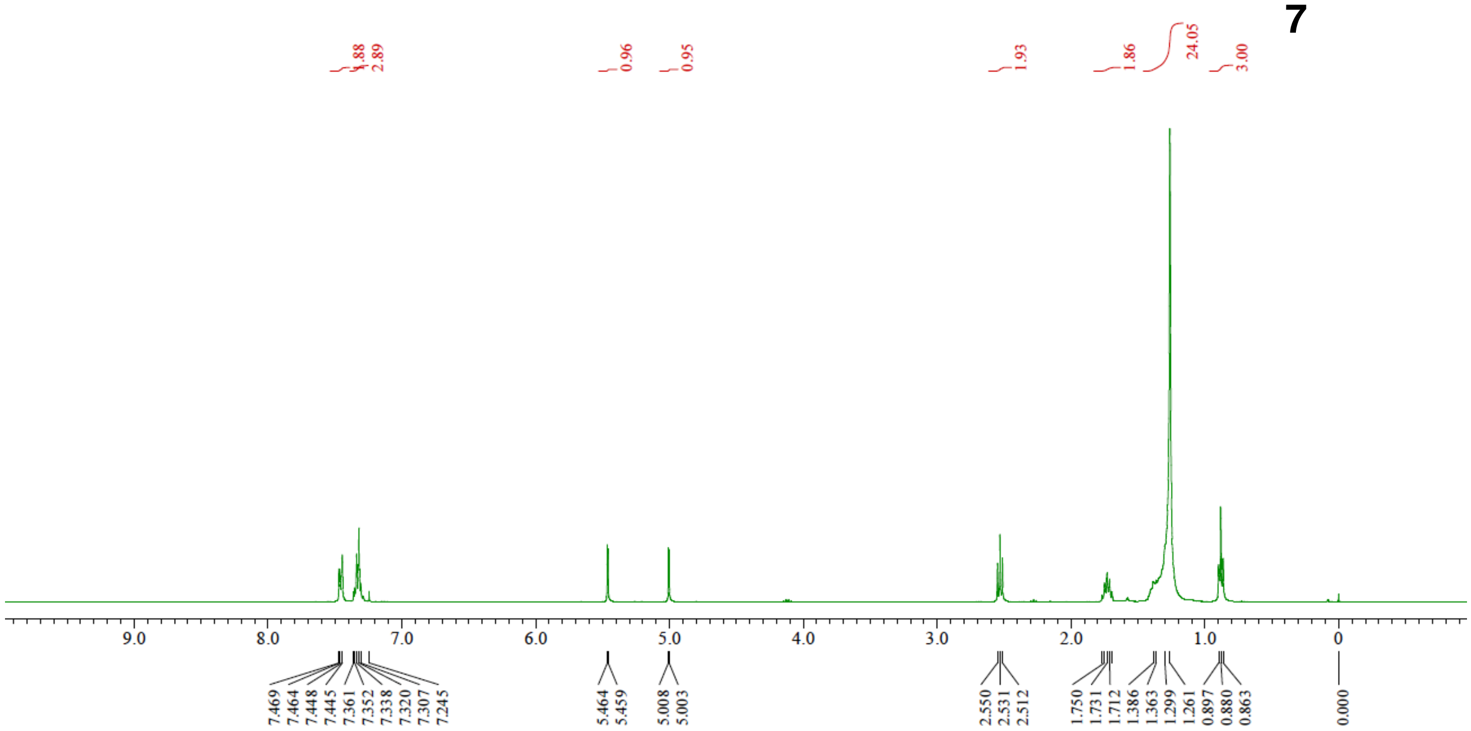

^{13}C NMR of **7**

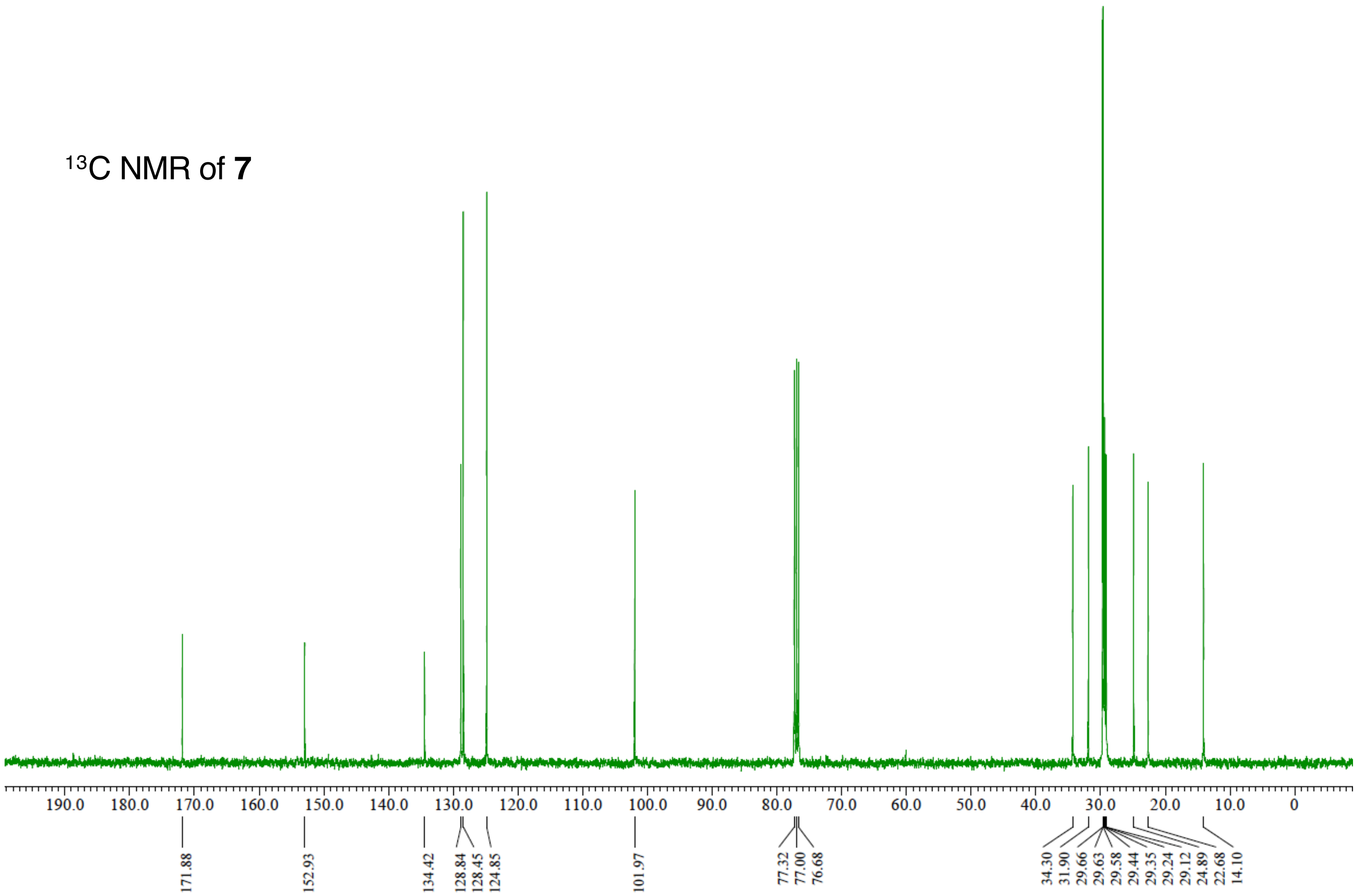

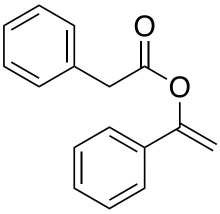

8

^{1}H NMR of **8**

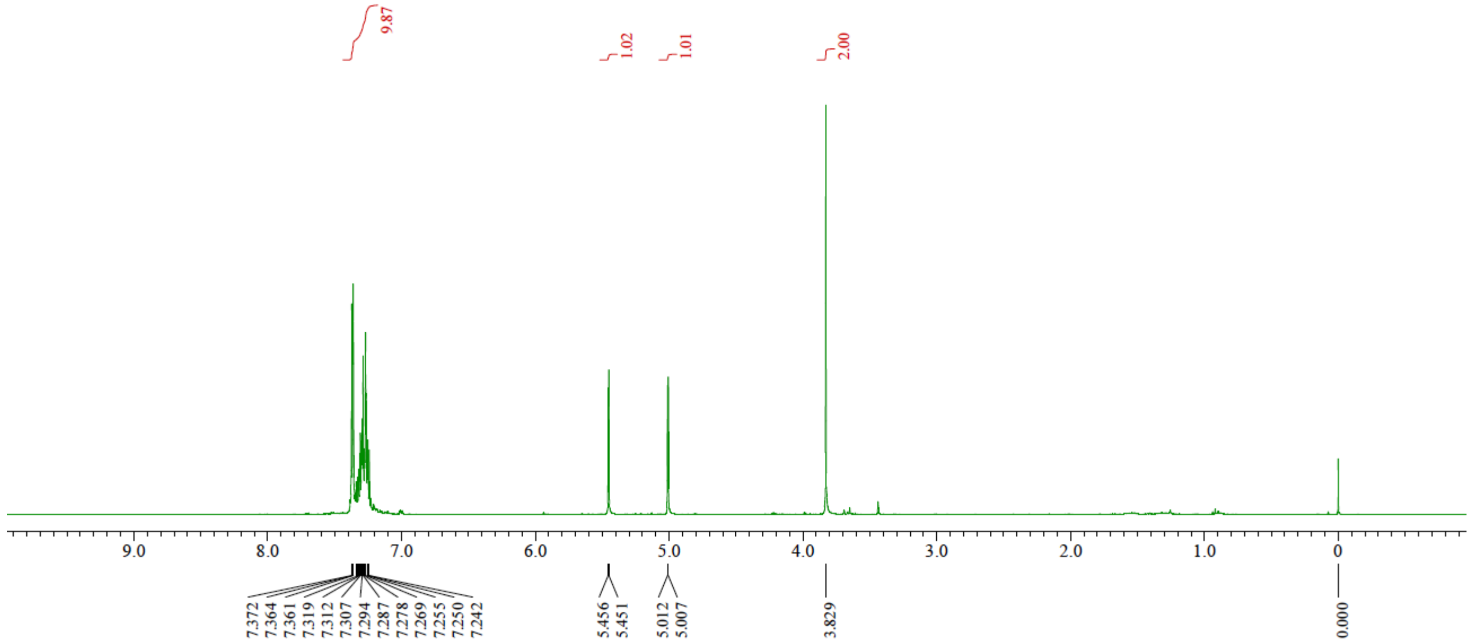

^{13}C NMR of **8**

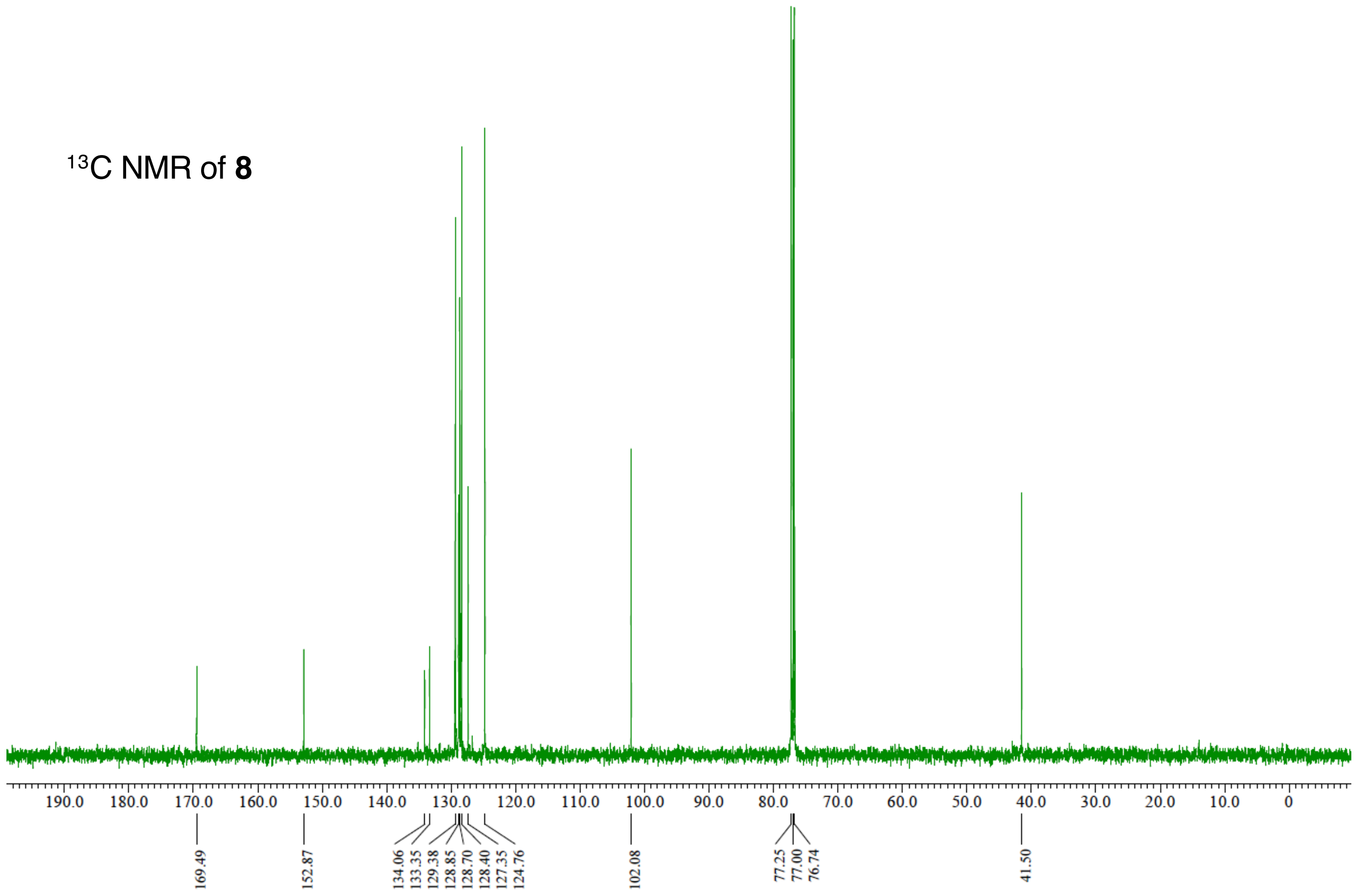

^{1}H NMR of **9**

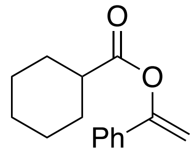

9

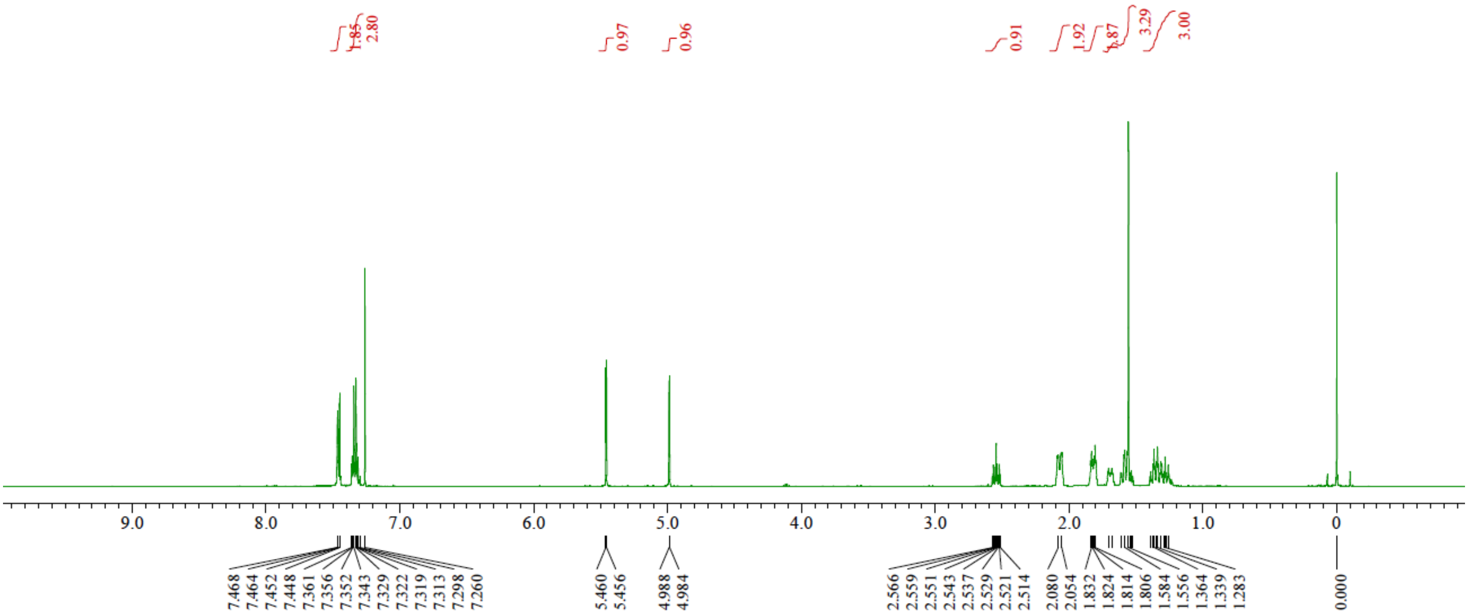

^{13}C NMR of **9**

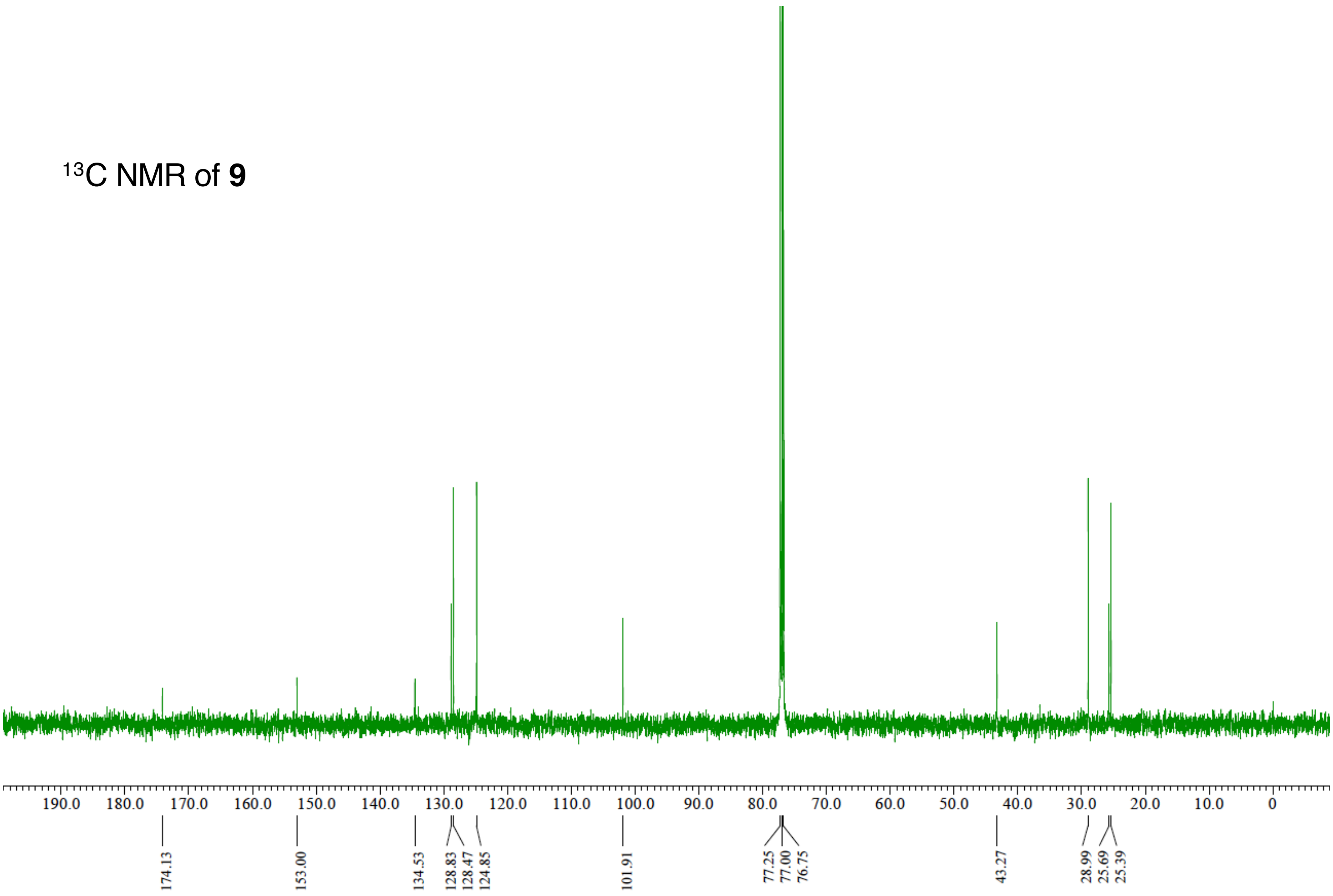

^{1}H NMR of **10**

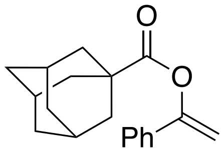

10

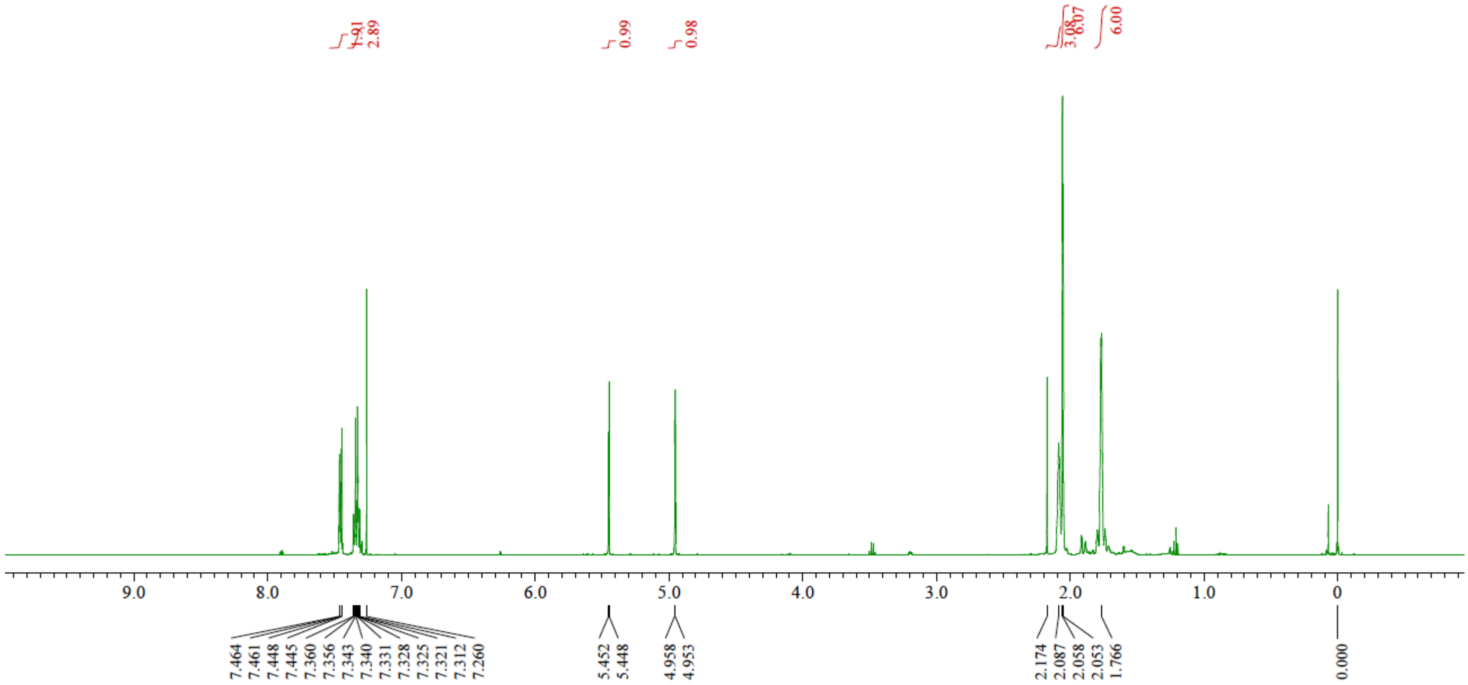

^{13}C NMR of **10**

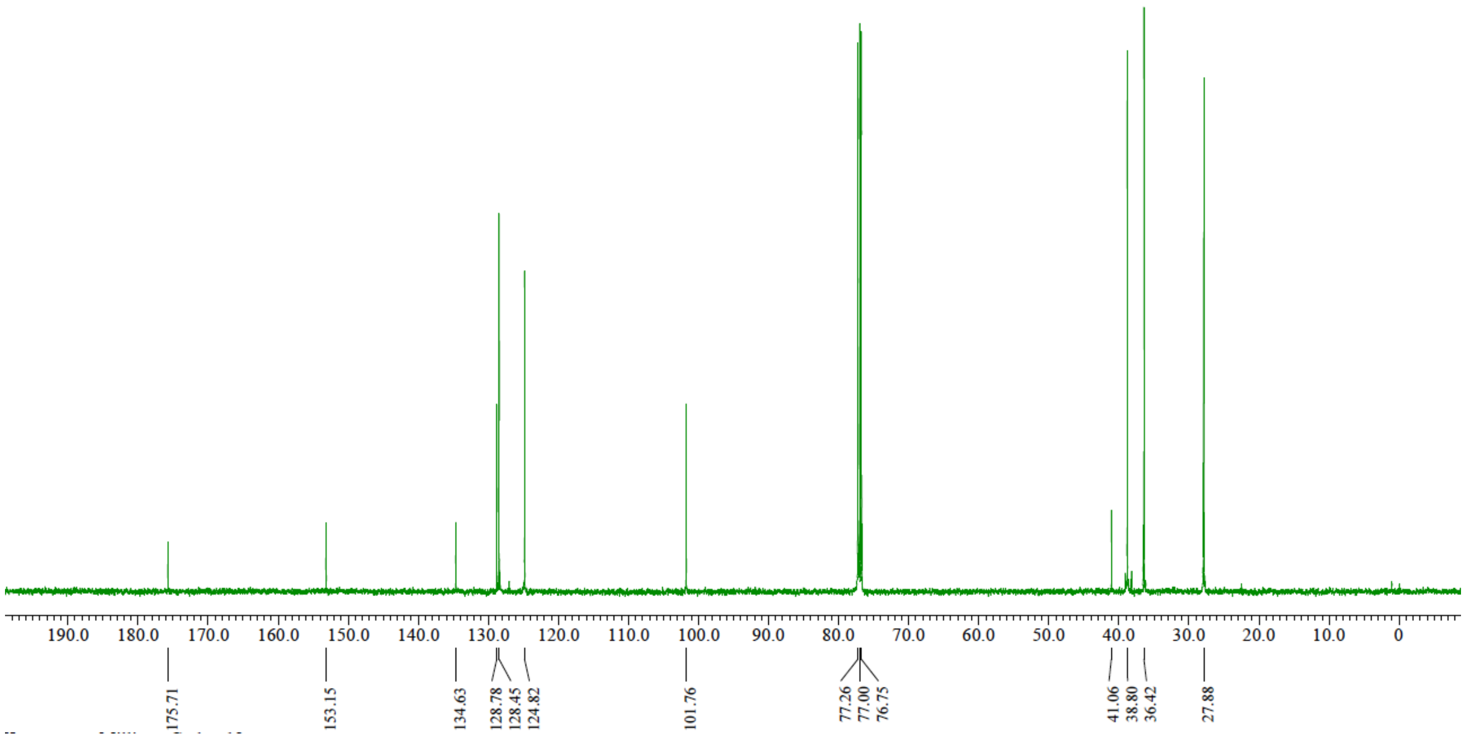

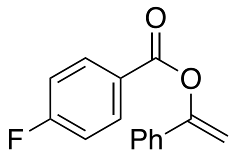

11

^{1}H NMR of **11**

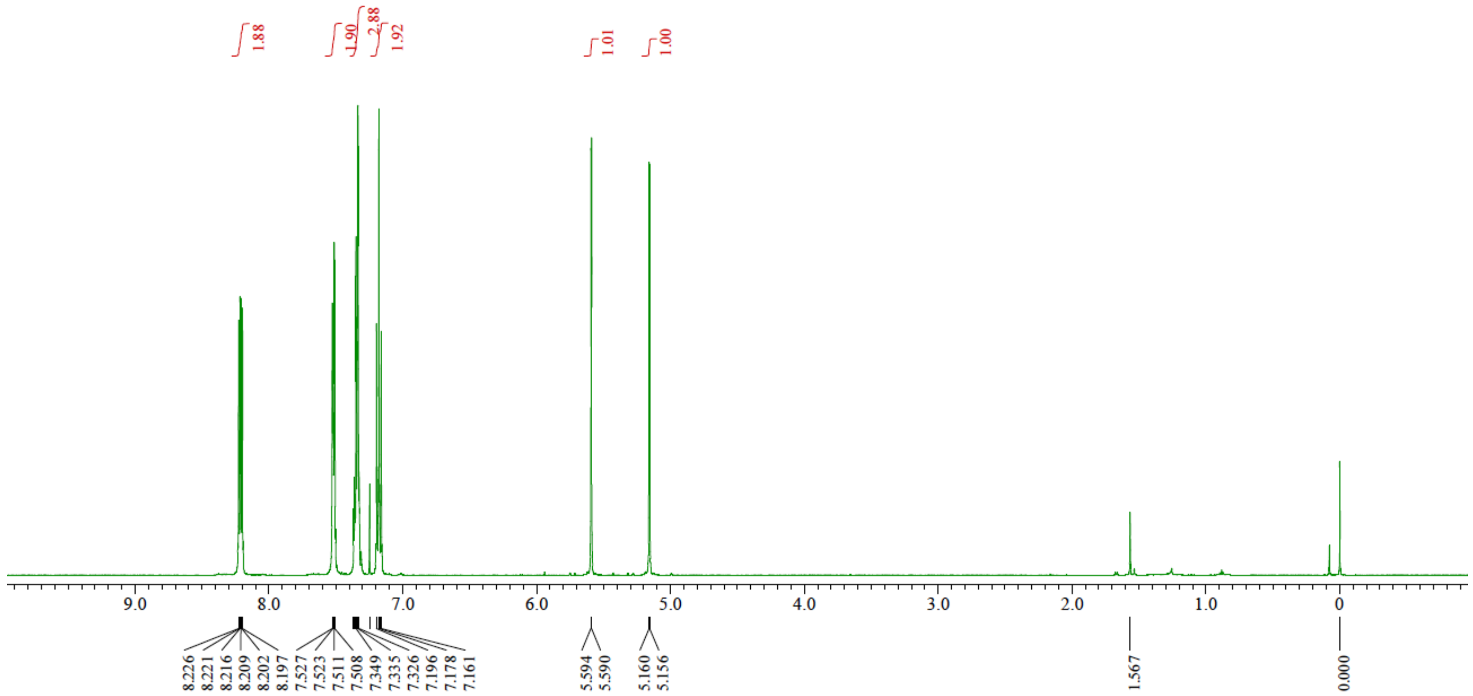

^{13}C NMR of **11**

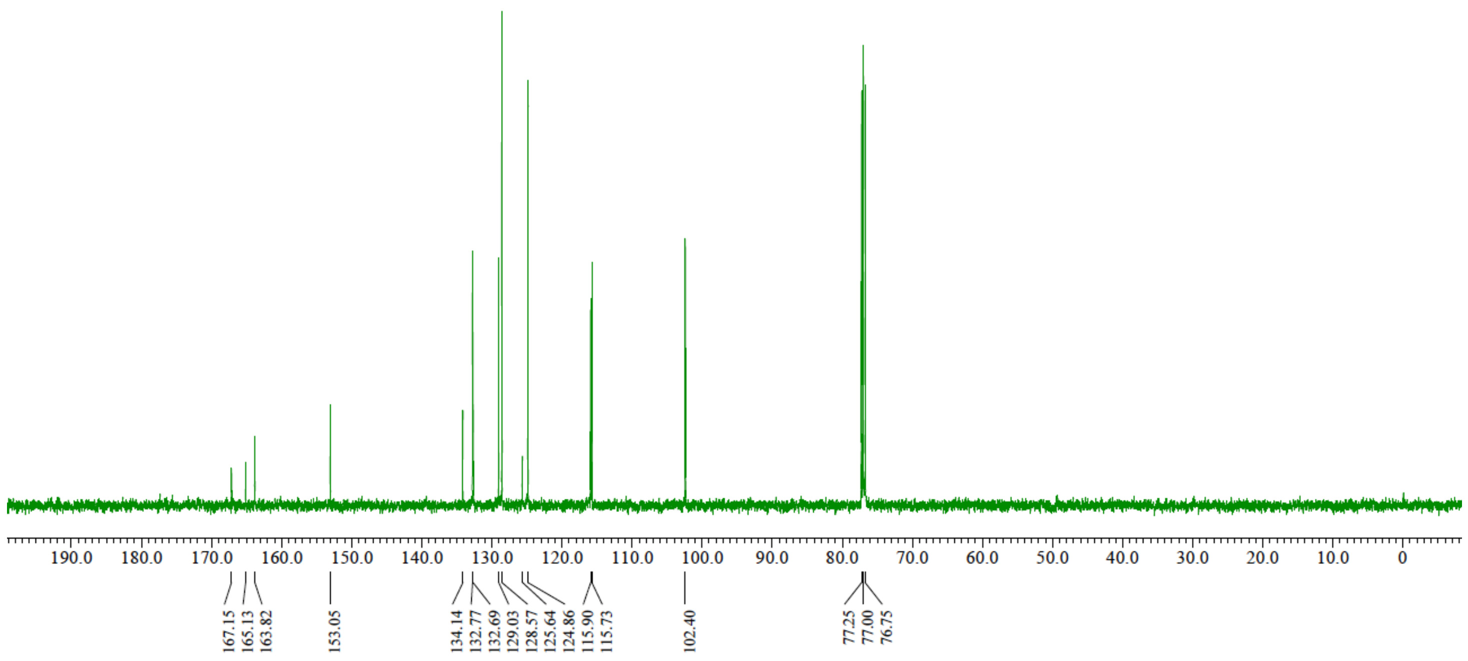

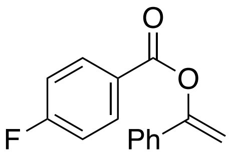

11

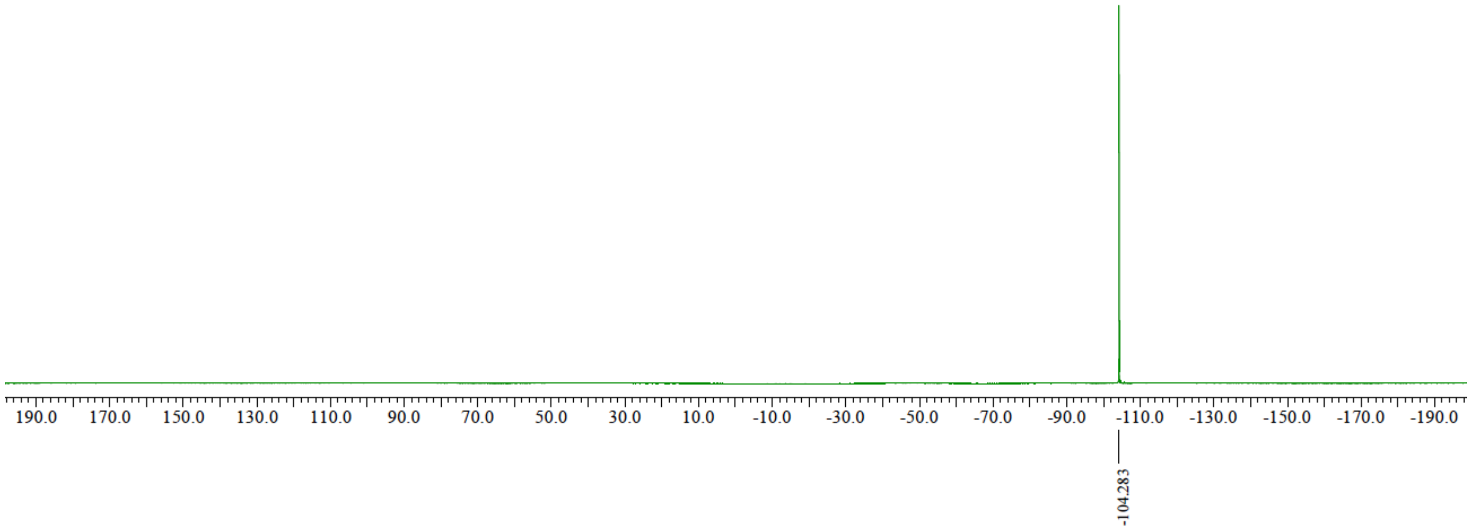

^{19}F NMR of **11**

¹H NMR of **12**

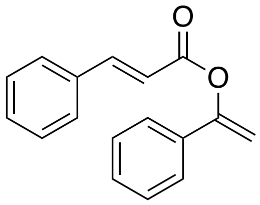

12

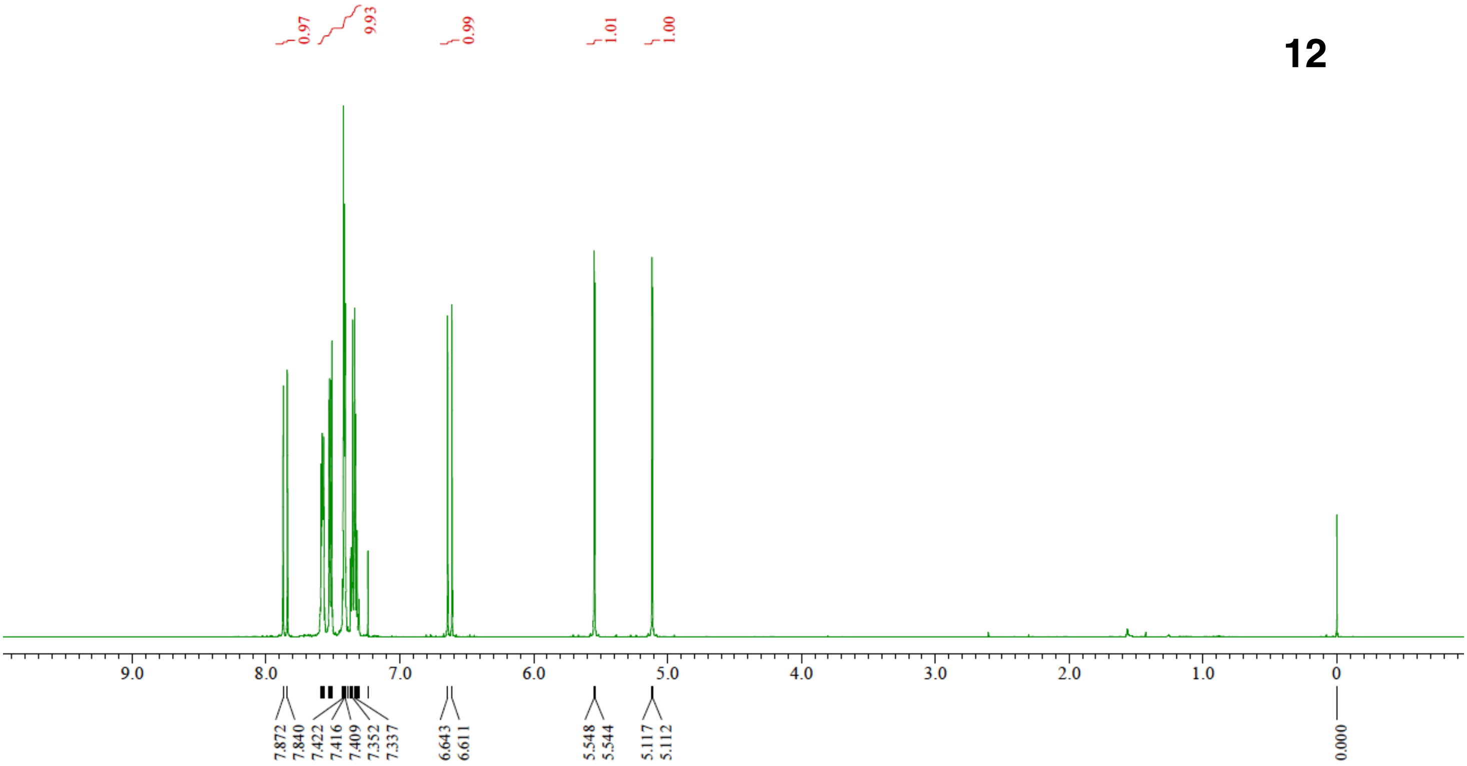

¹³C NMR of **12**

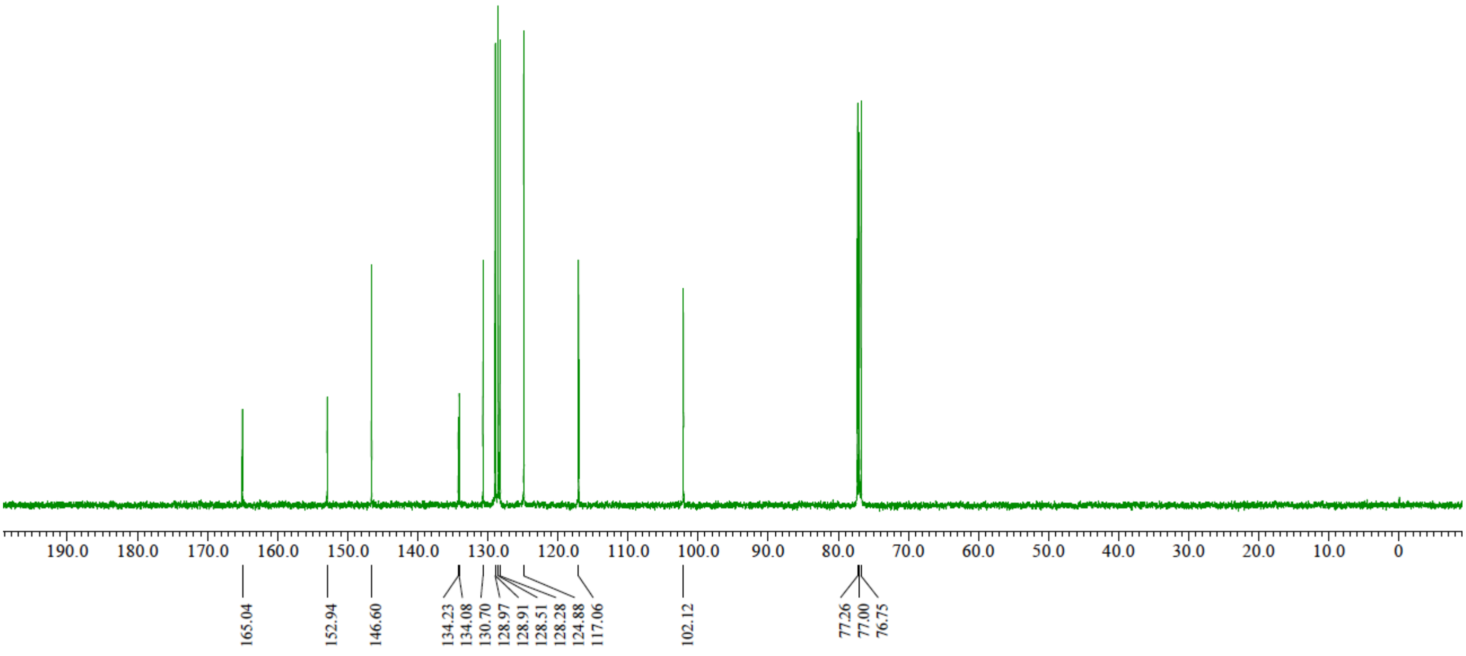

^{1}H NMR of **13**

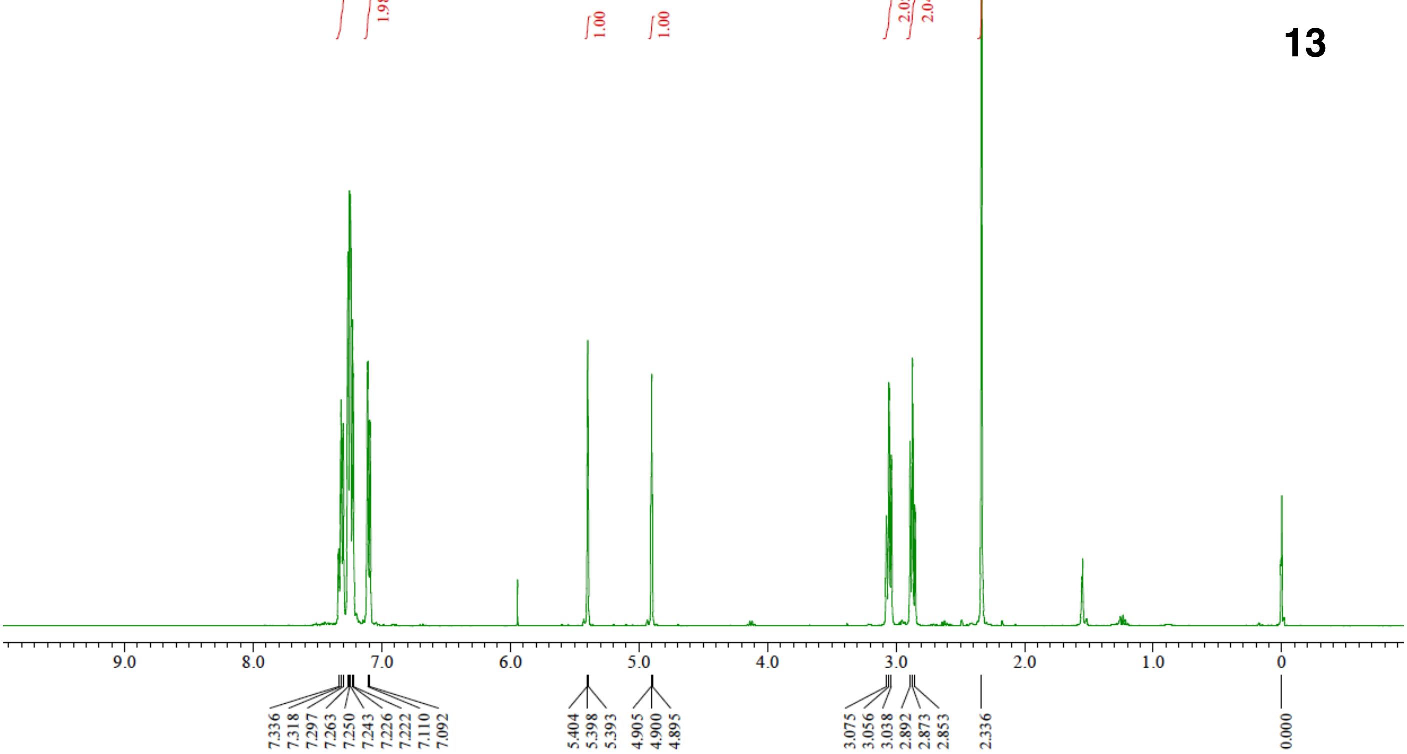

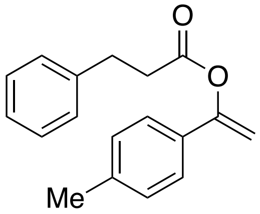

13

^{13}C NMR of **13**

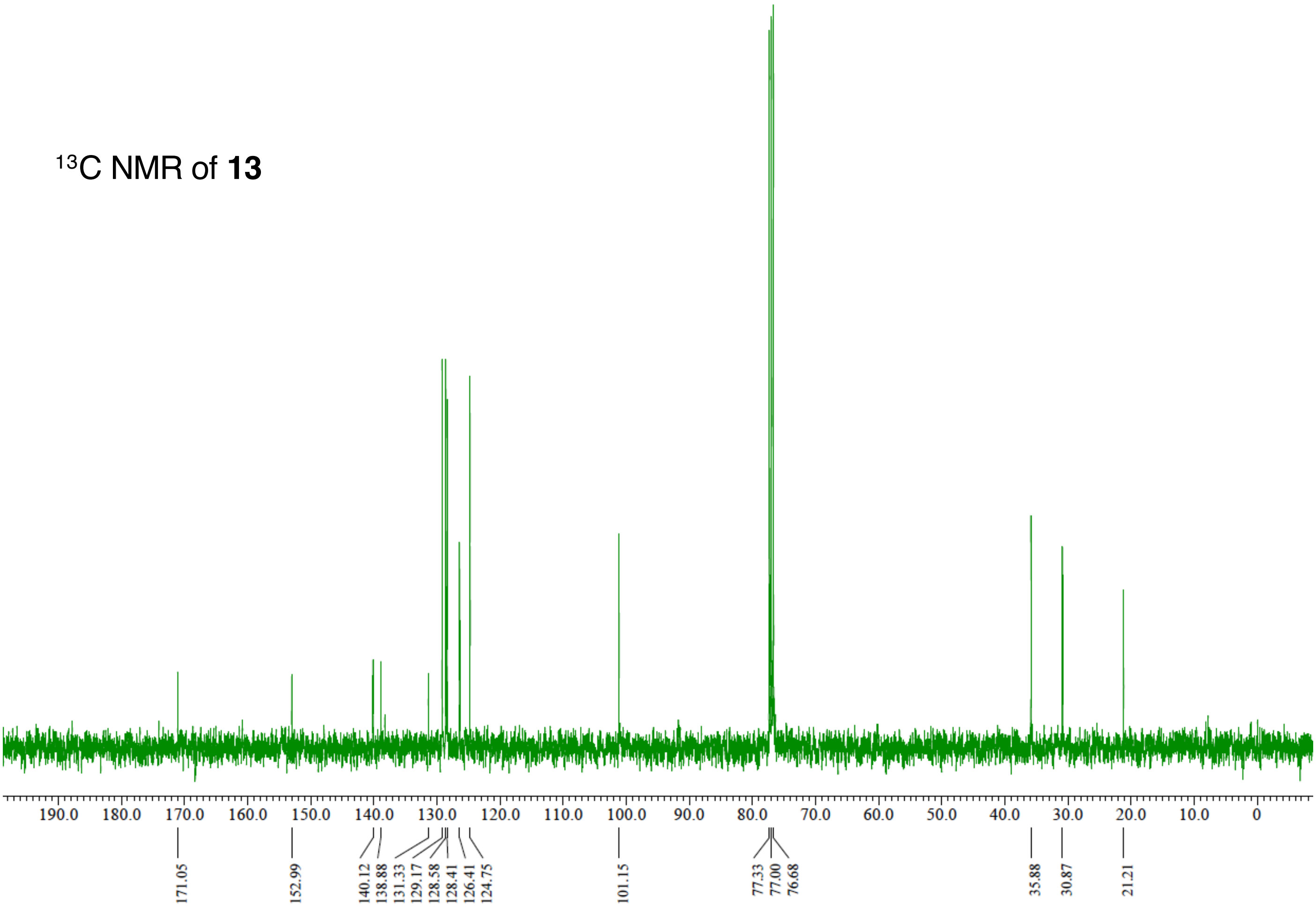

^{1}H NMR of **14**

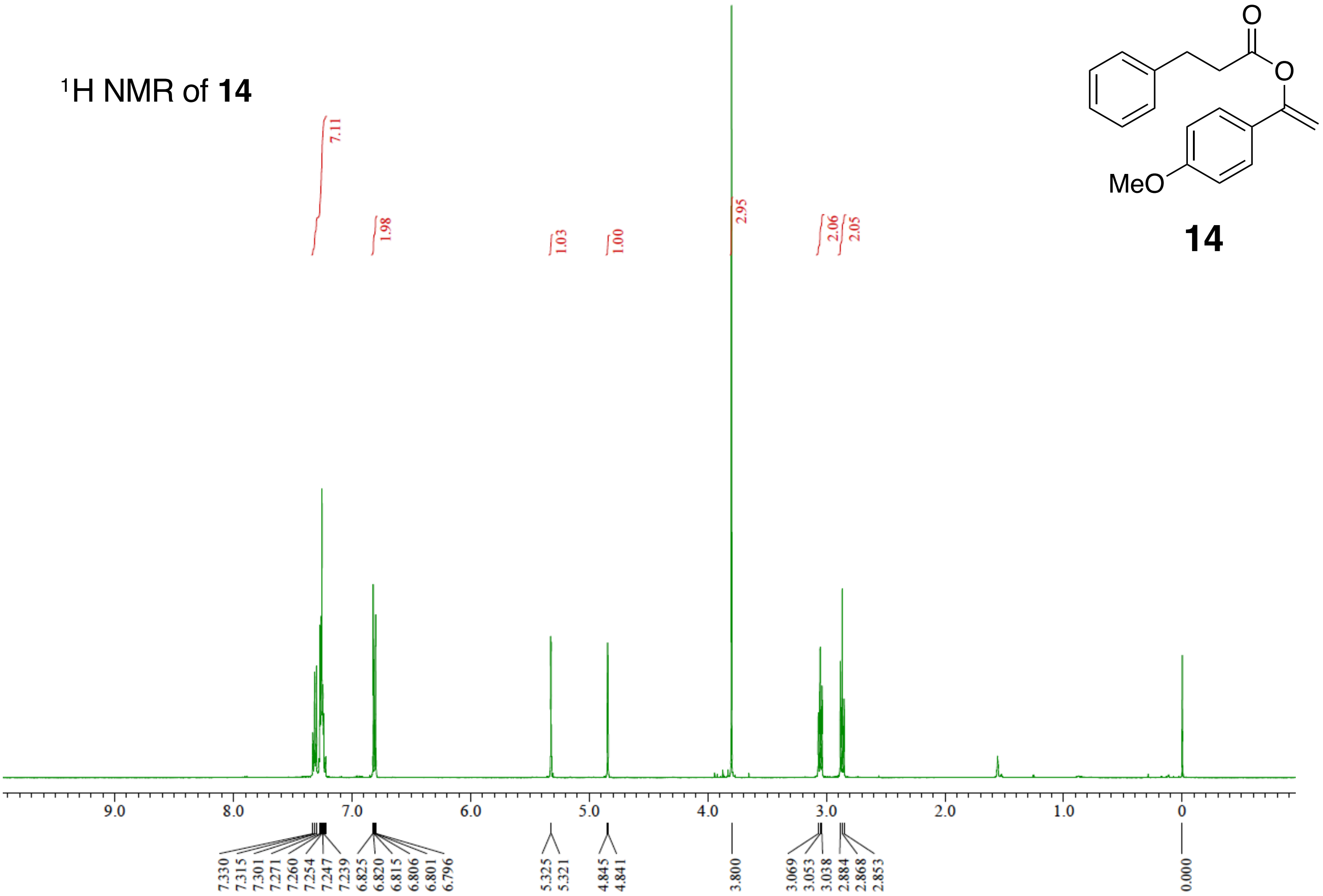

14

^{13}C NMR of **14**

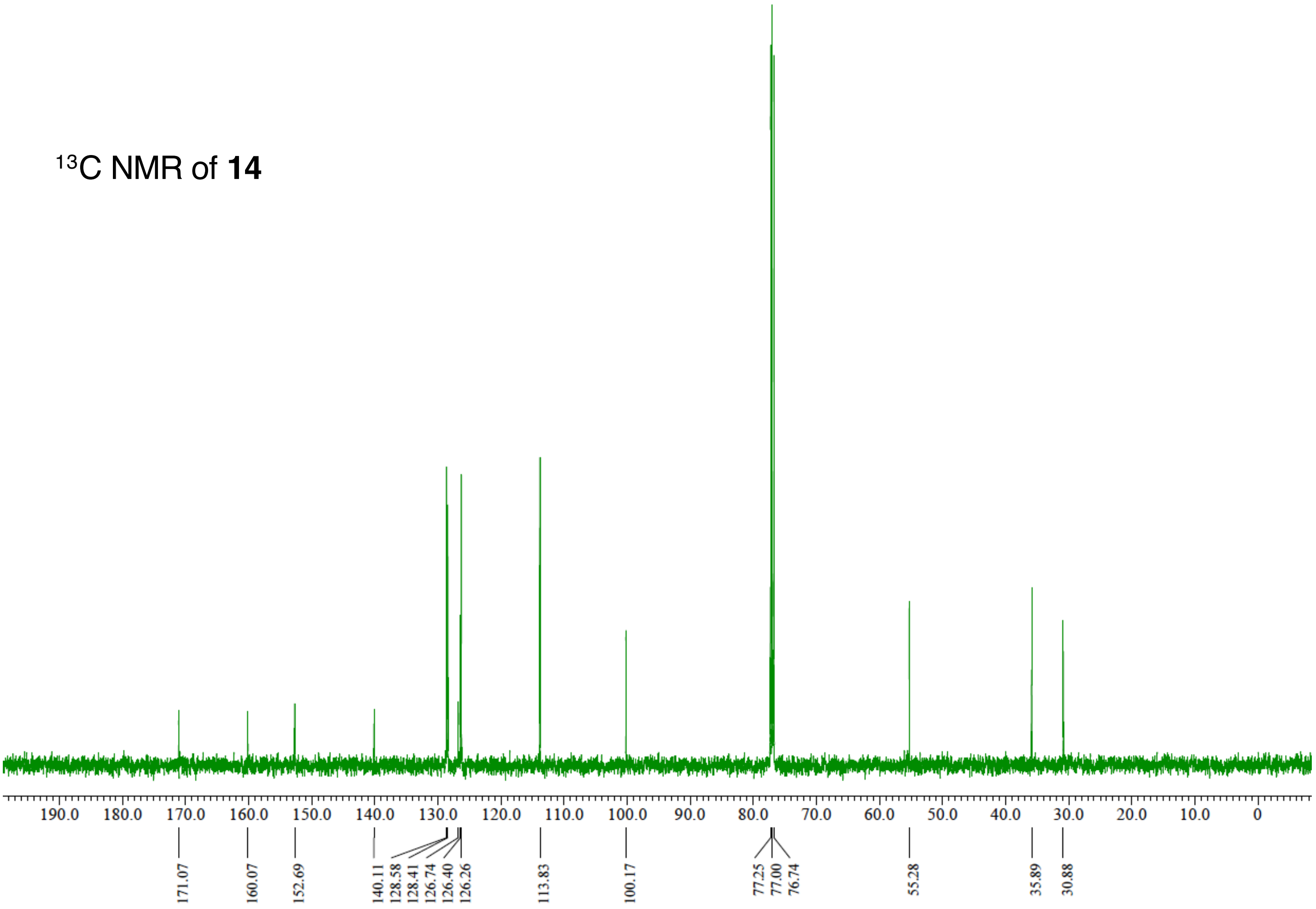

^{1}H NMR of **15**

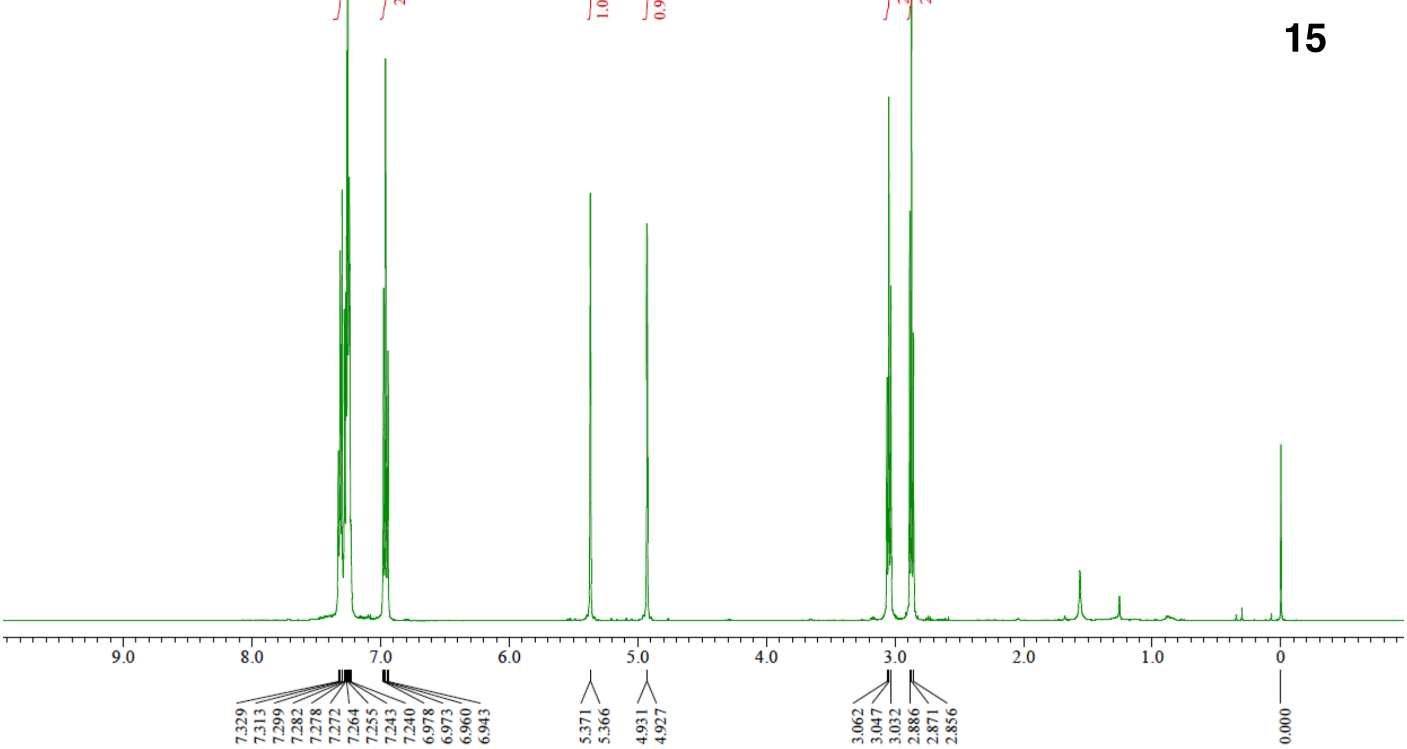

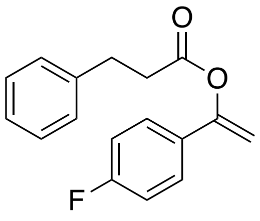

15

^{13}C NMR of **15**

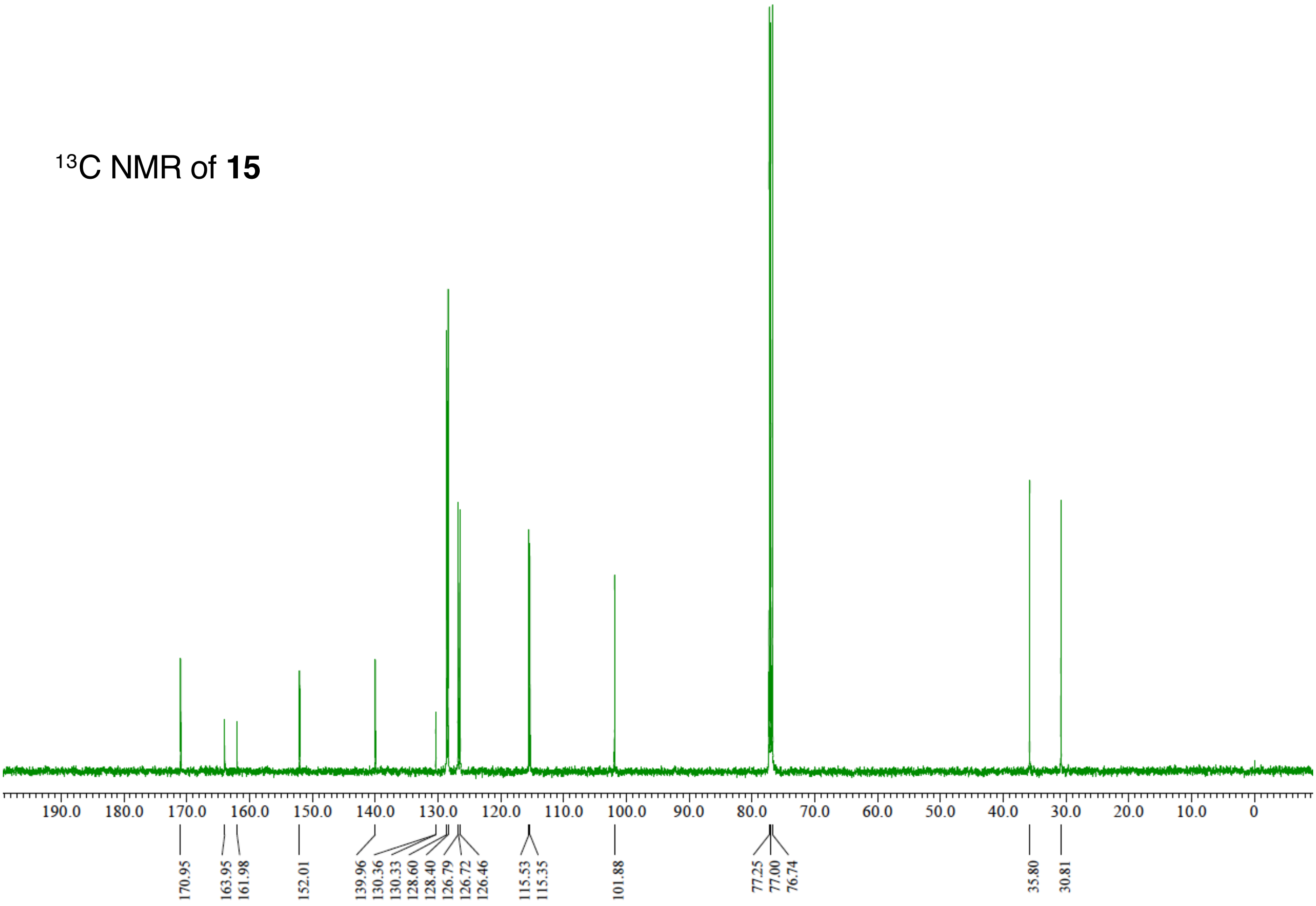

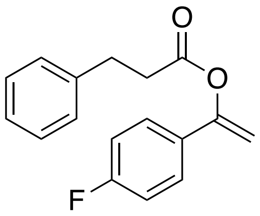

15

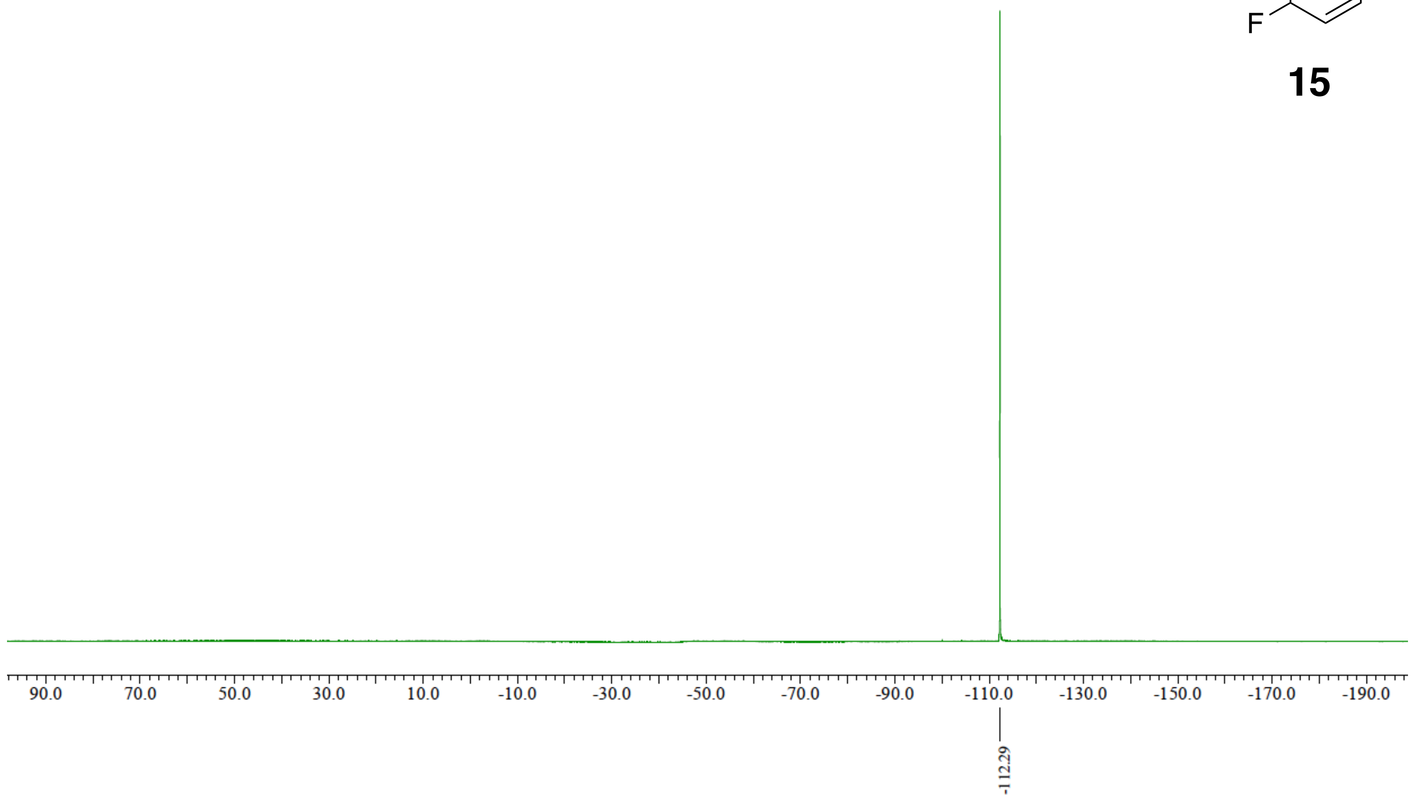

^{19}F NMR of **15**

^{1}H NMR of **16**

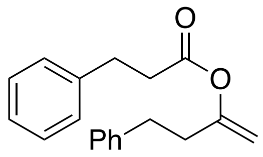

16

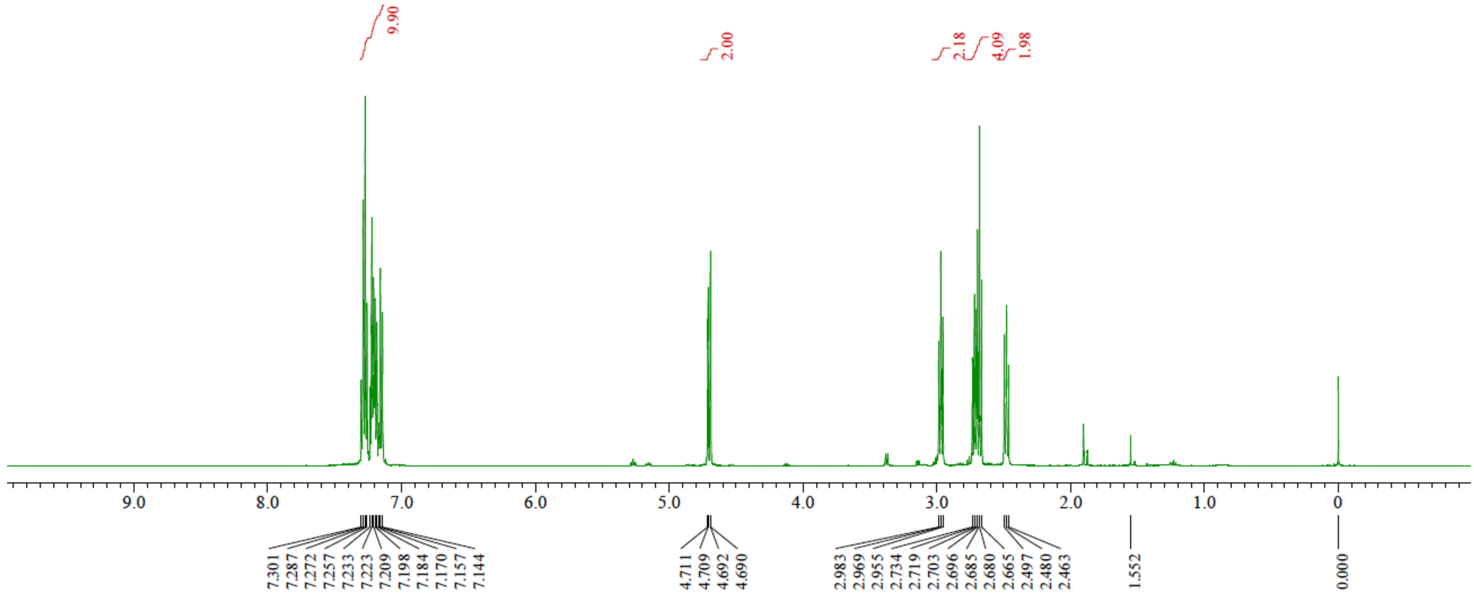

^{13}C NMR of **16**

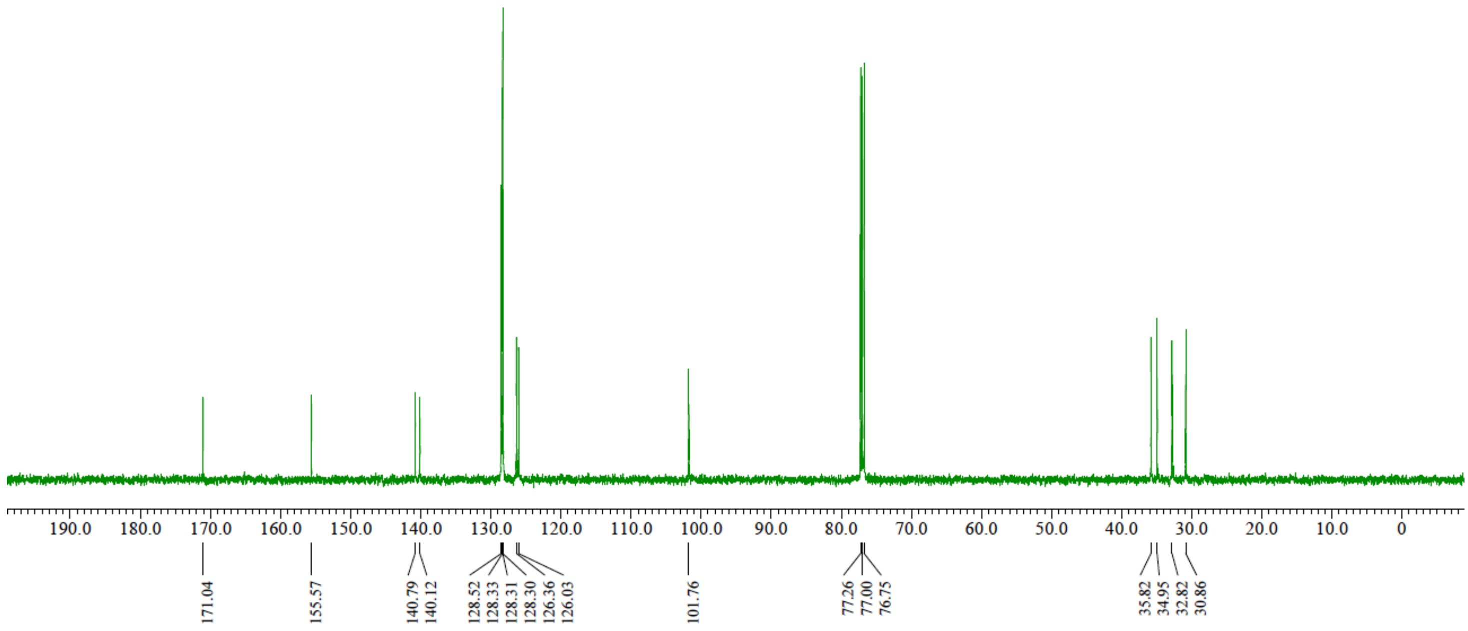

^{1}H NMR of **17**

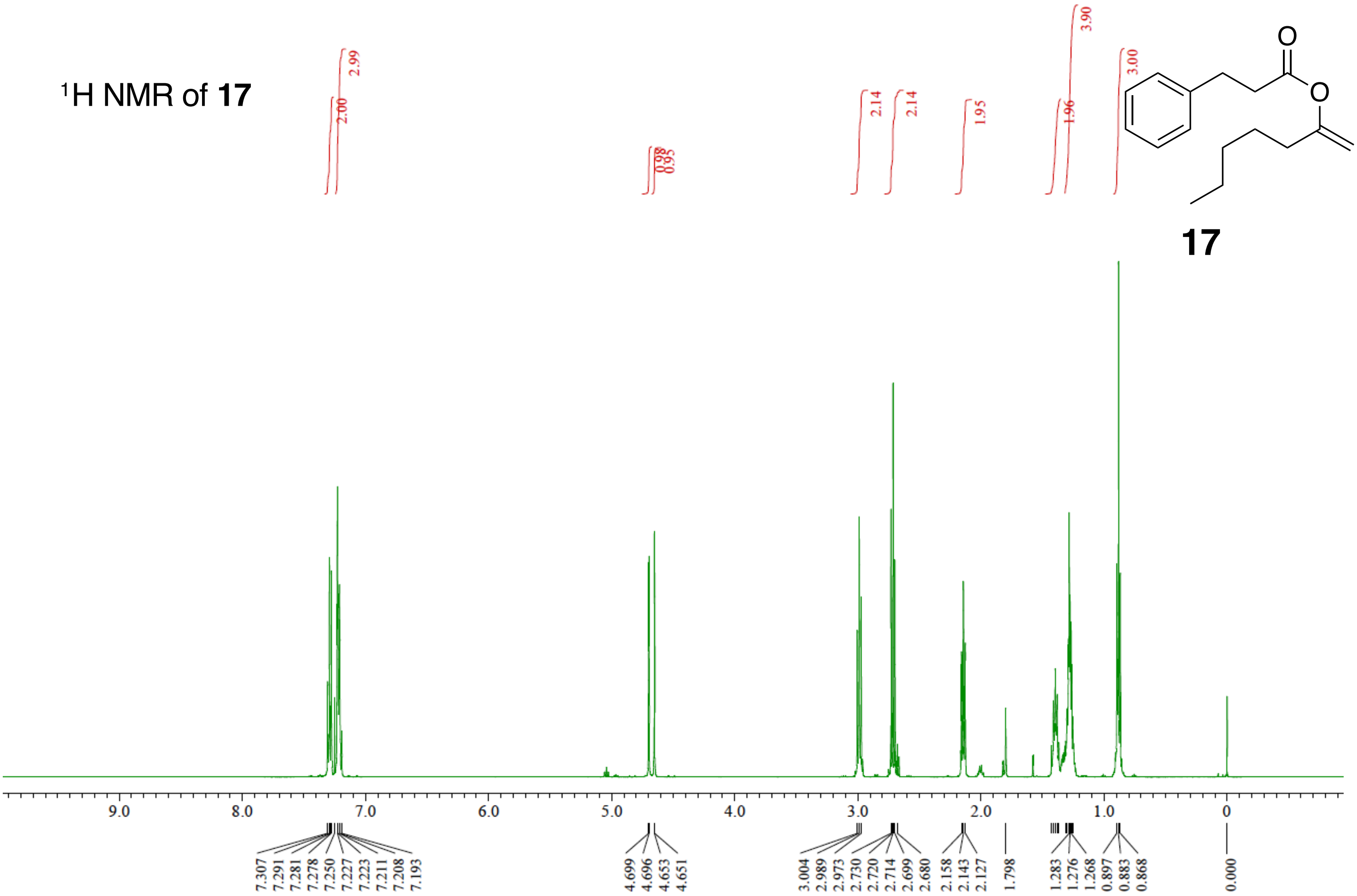

17

^{13}C NMR of **17**

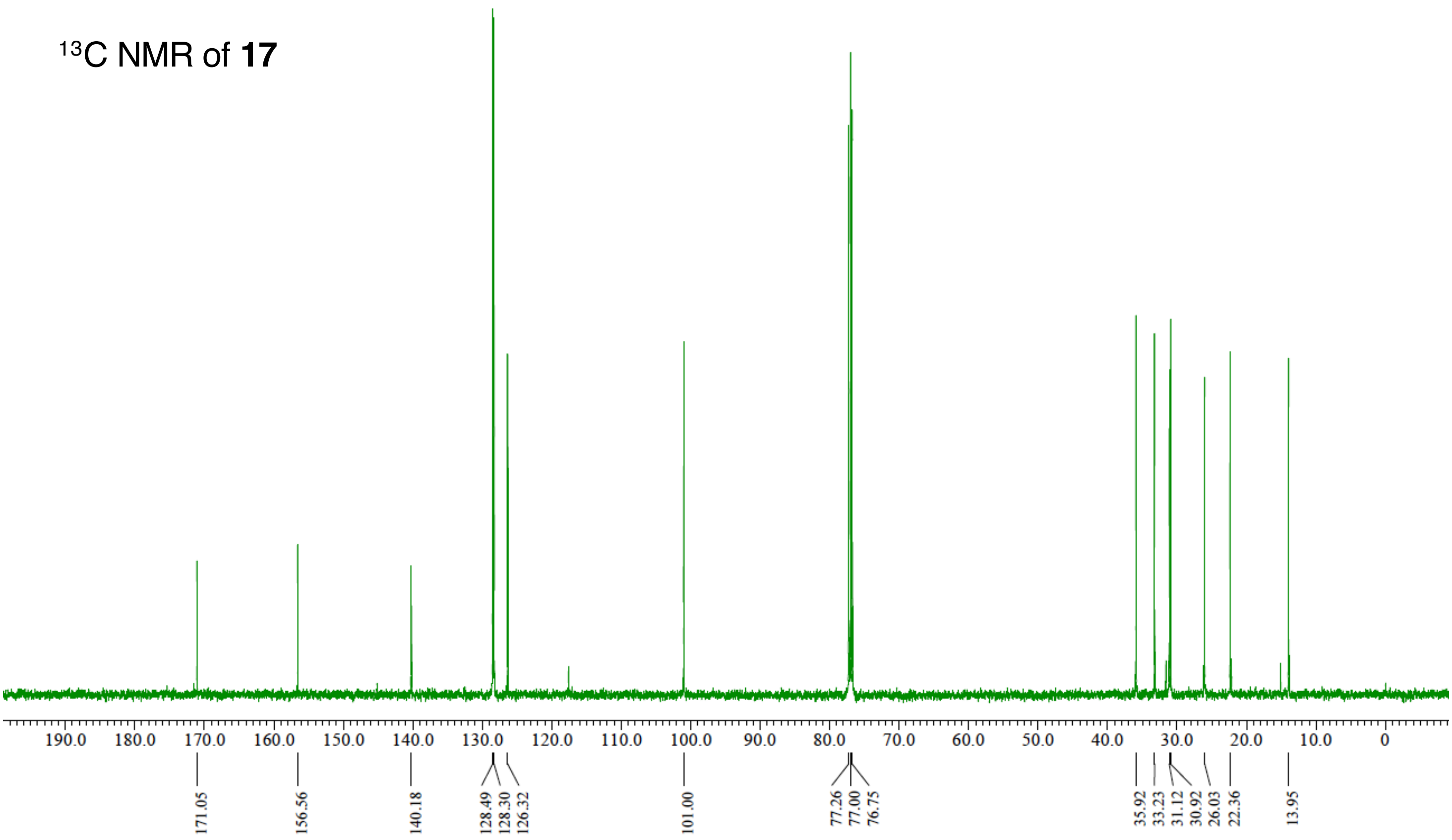

^{1}H NMR of **18**

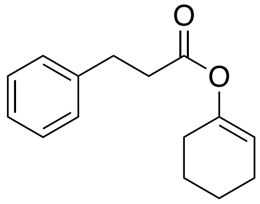

18

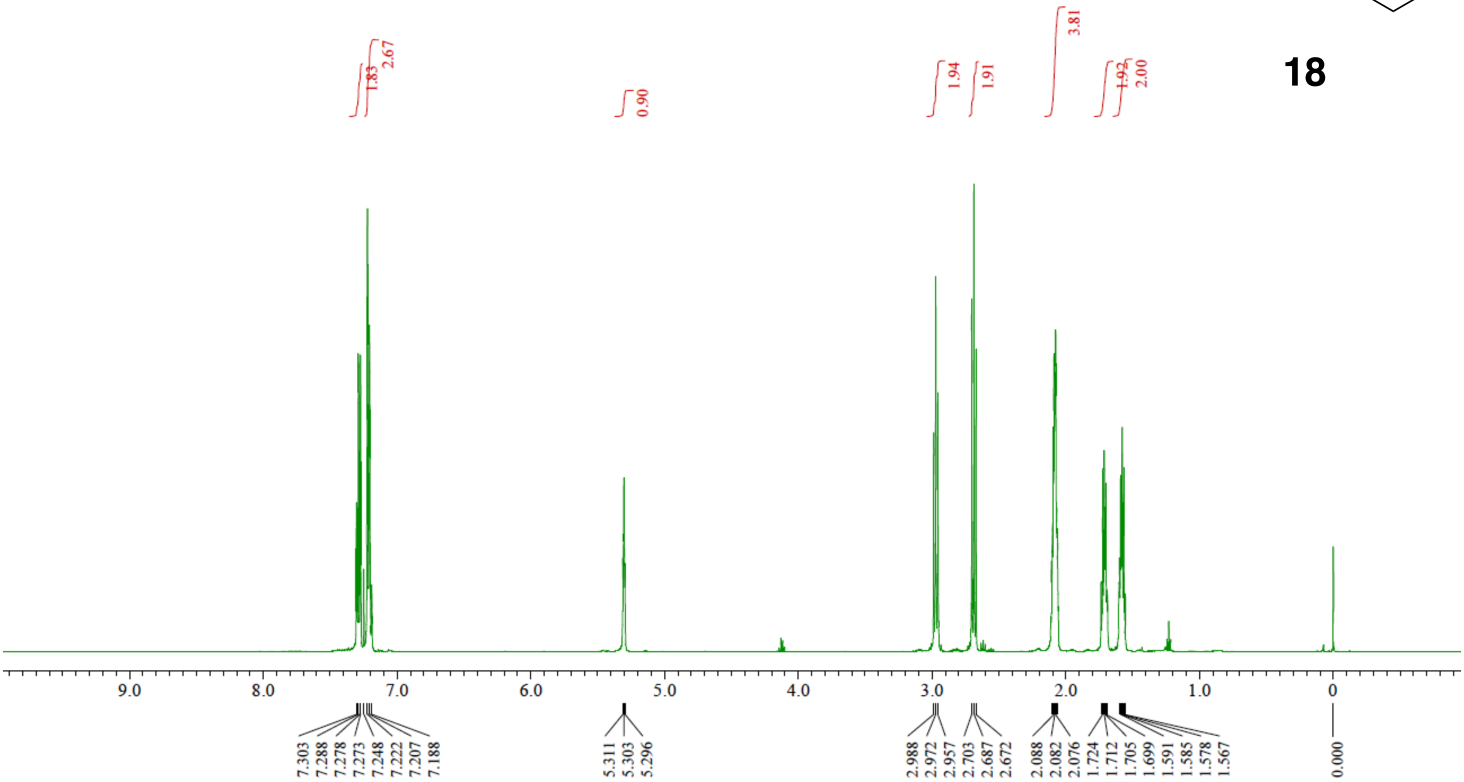

^{13}C NMR of **18**

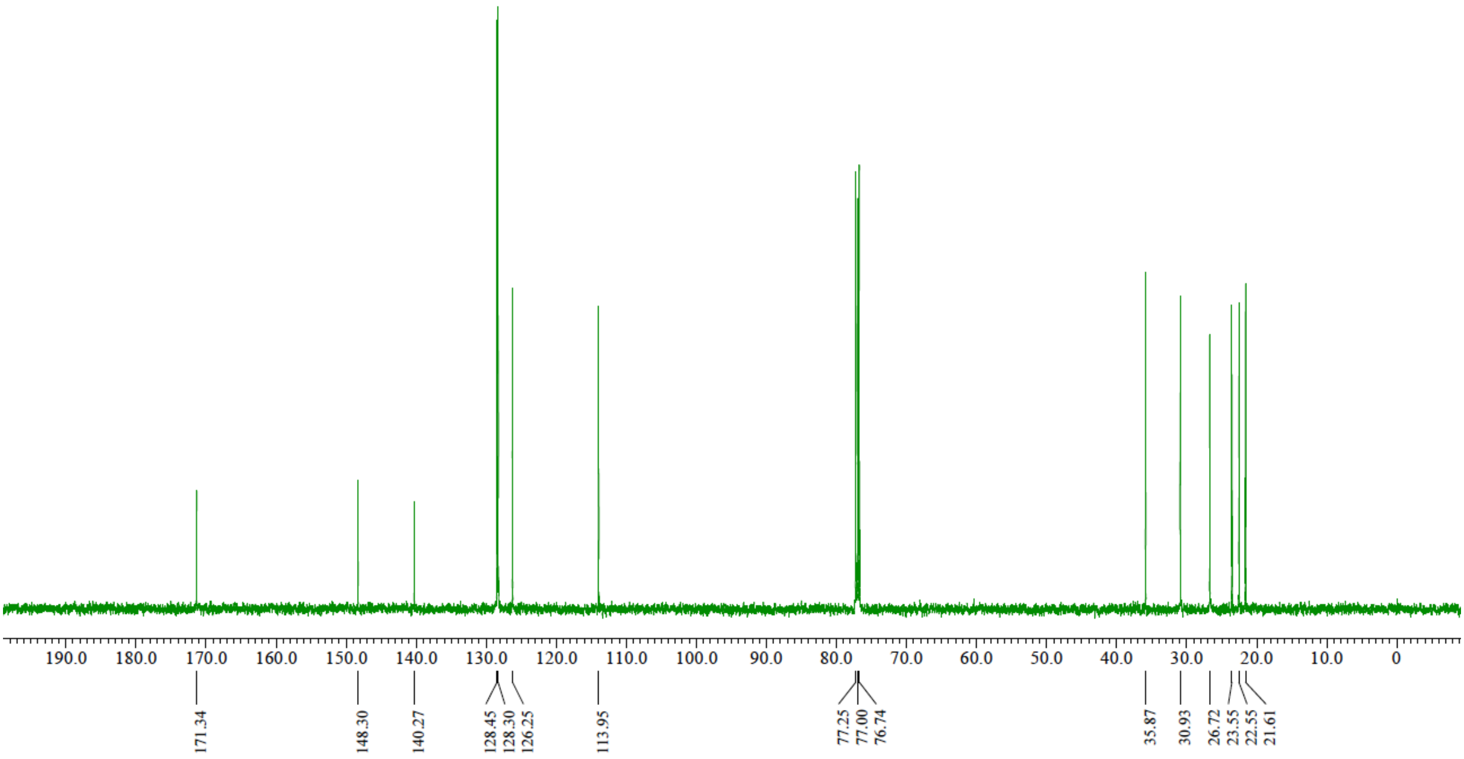

^{1}H NMR of **19**

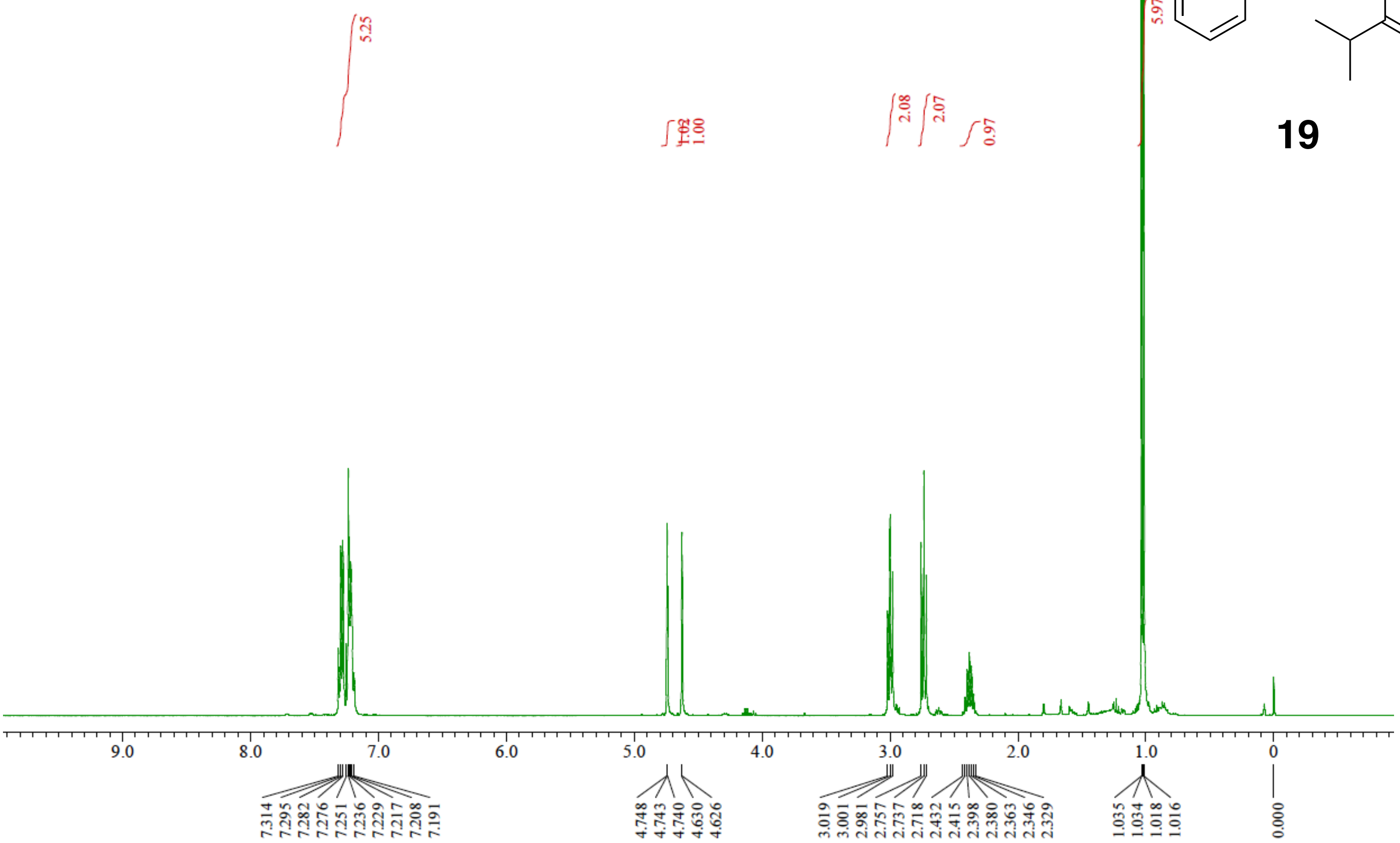

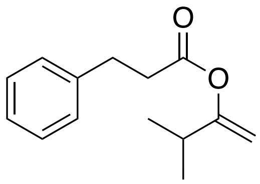

19

^{13}C NMR of **19**

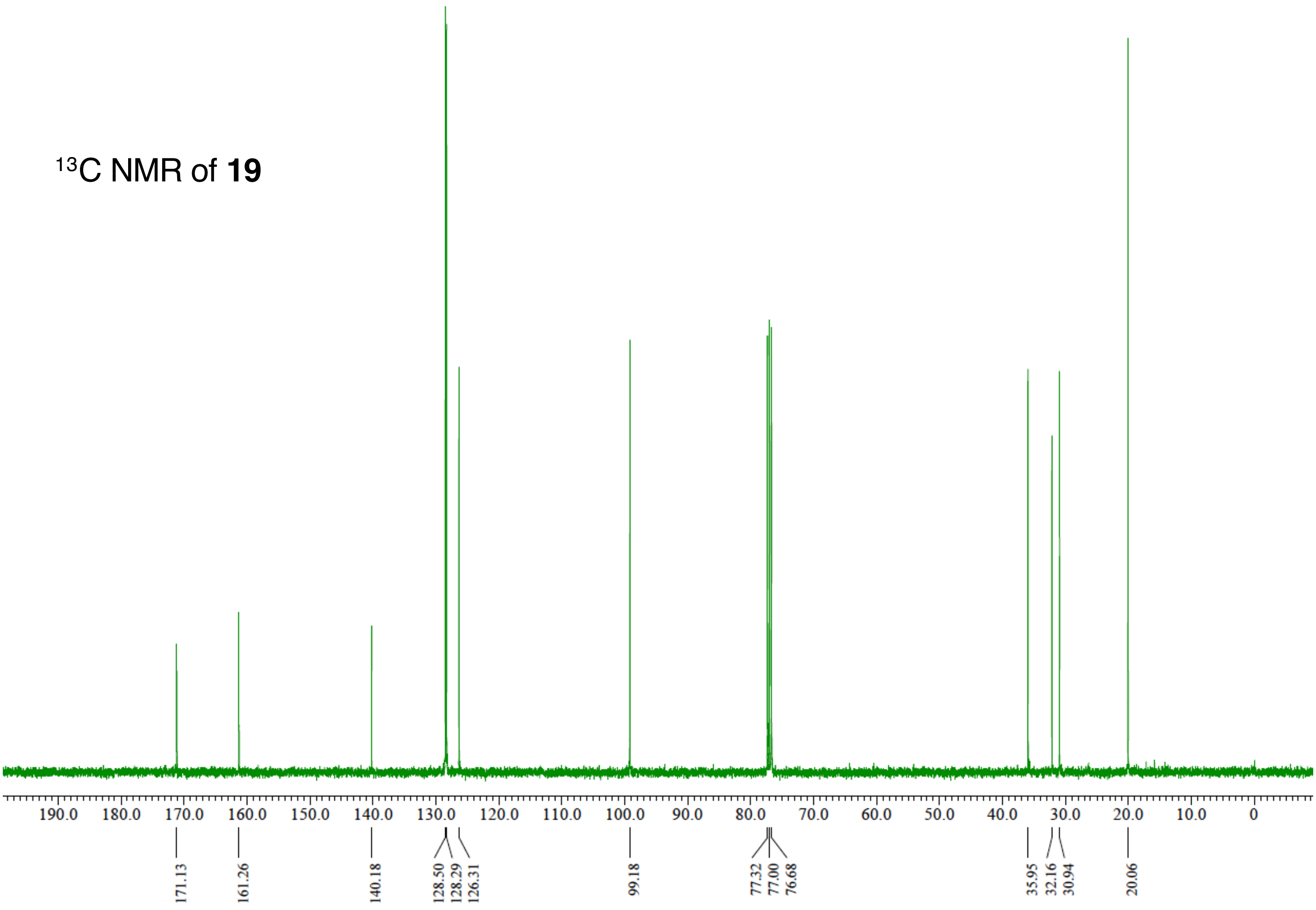

^{1}H NMR of **20**

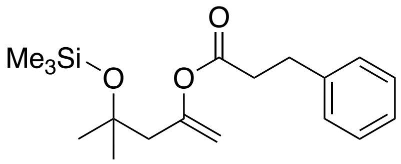

20

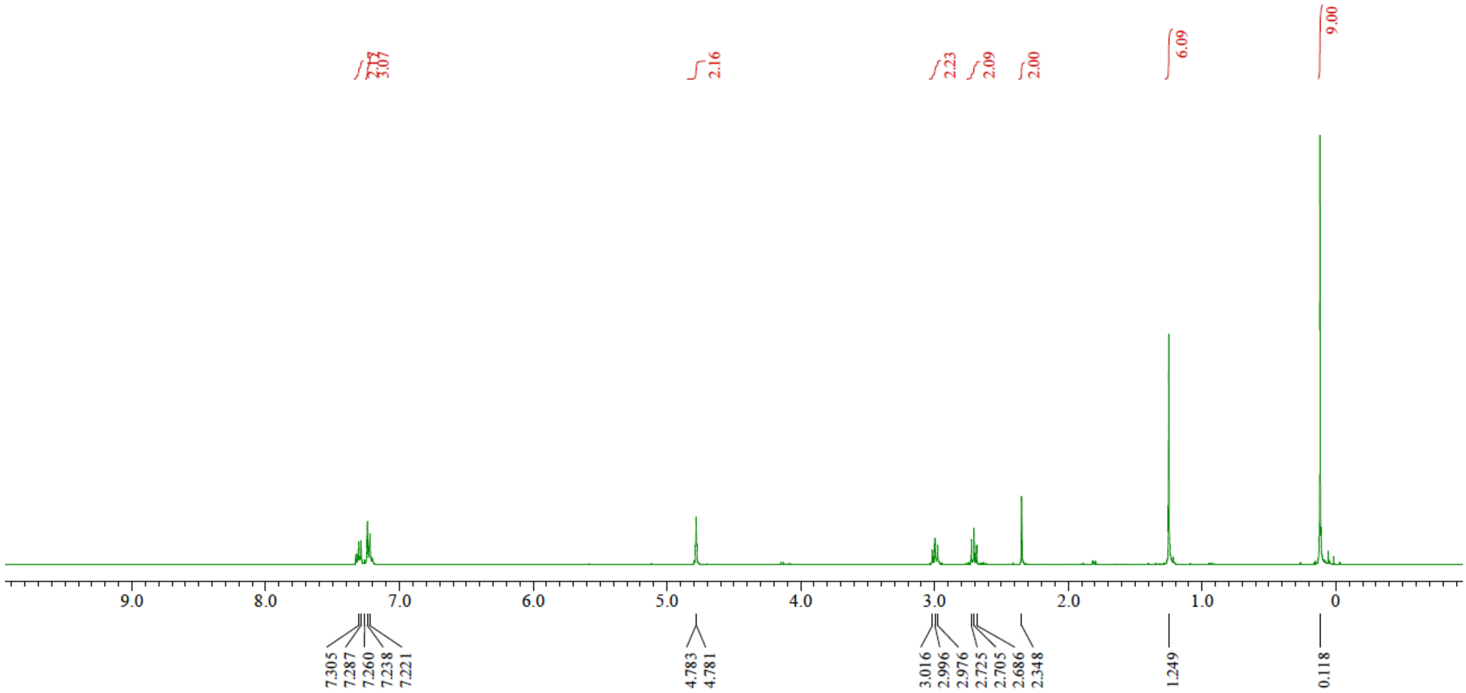

^{13}C NMR of **20**

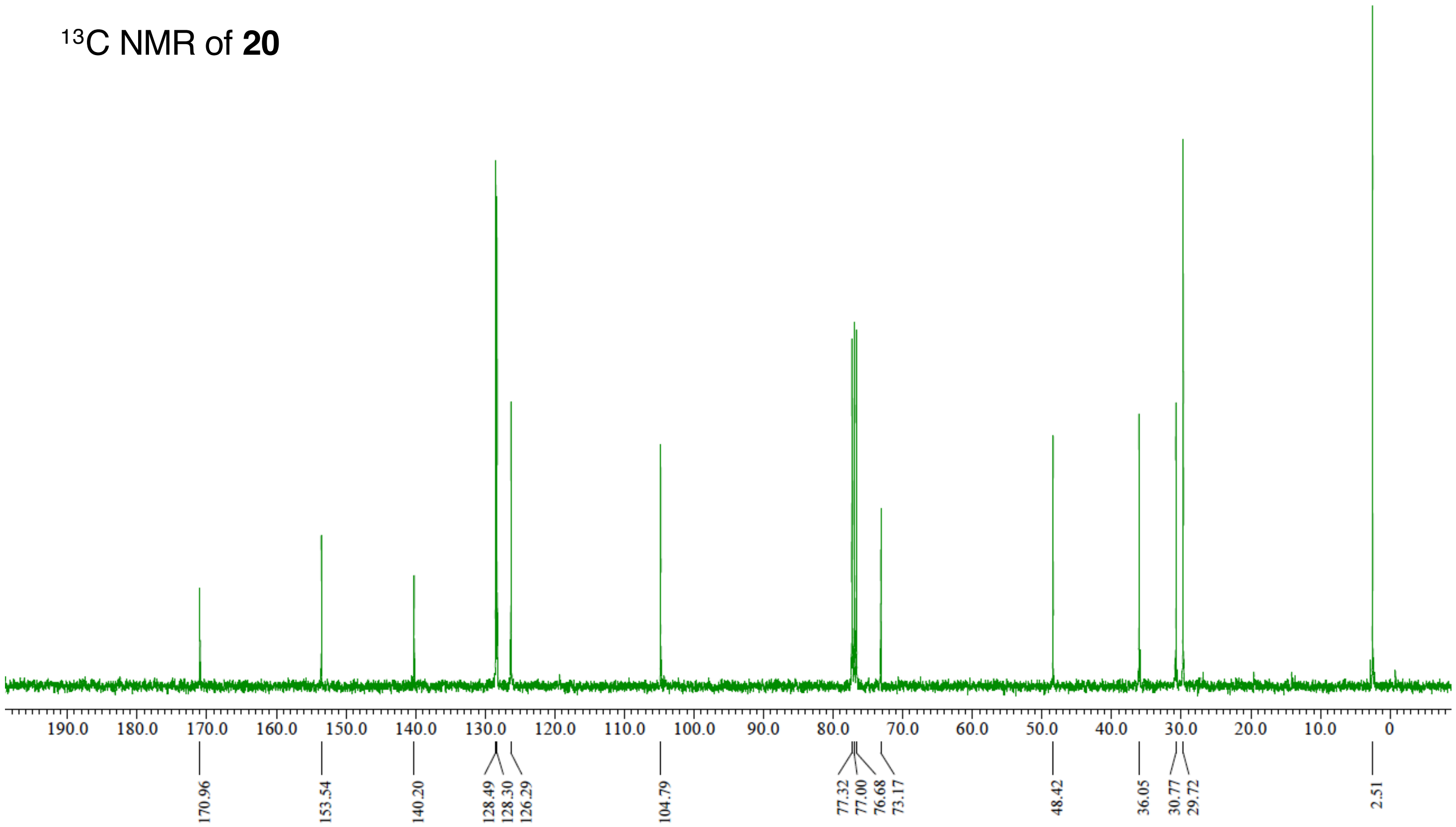

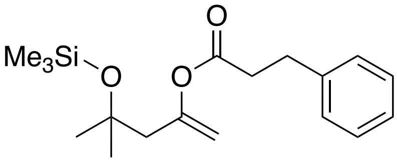

20

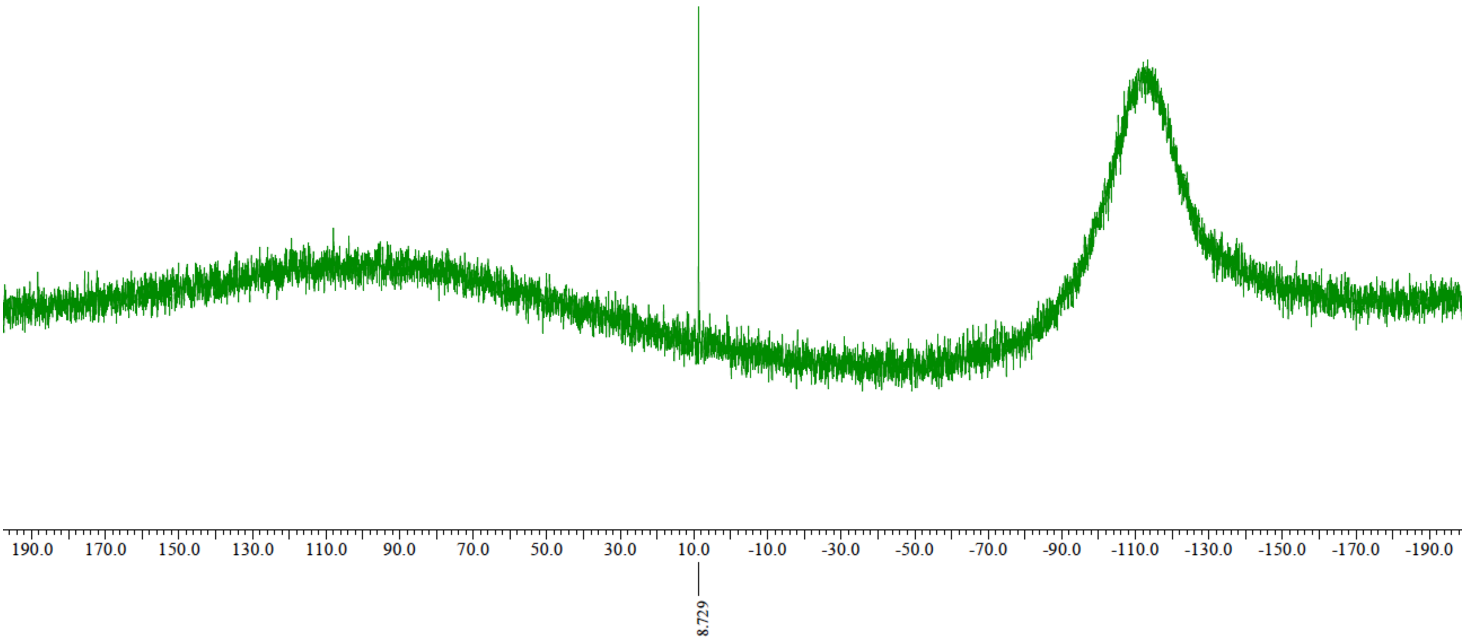

^{29}Si NMR of **20**

^{1}H NMR of **21**

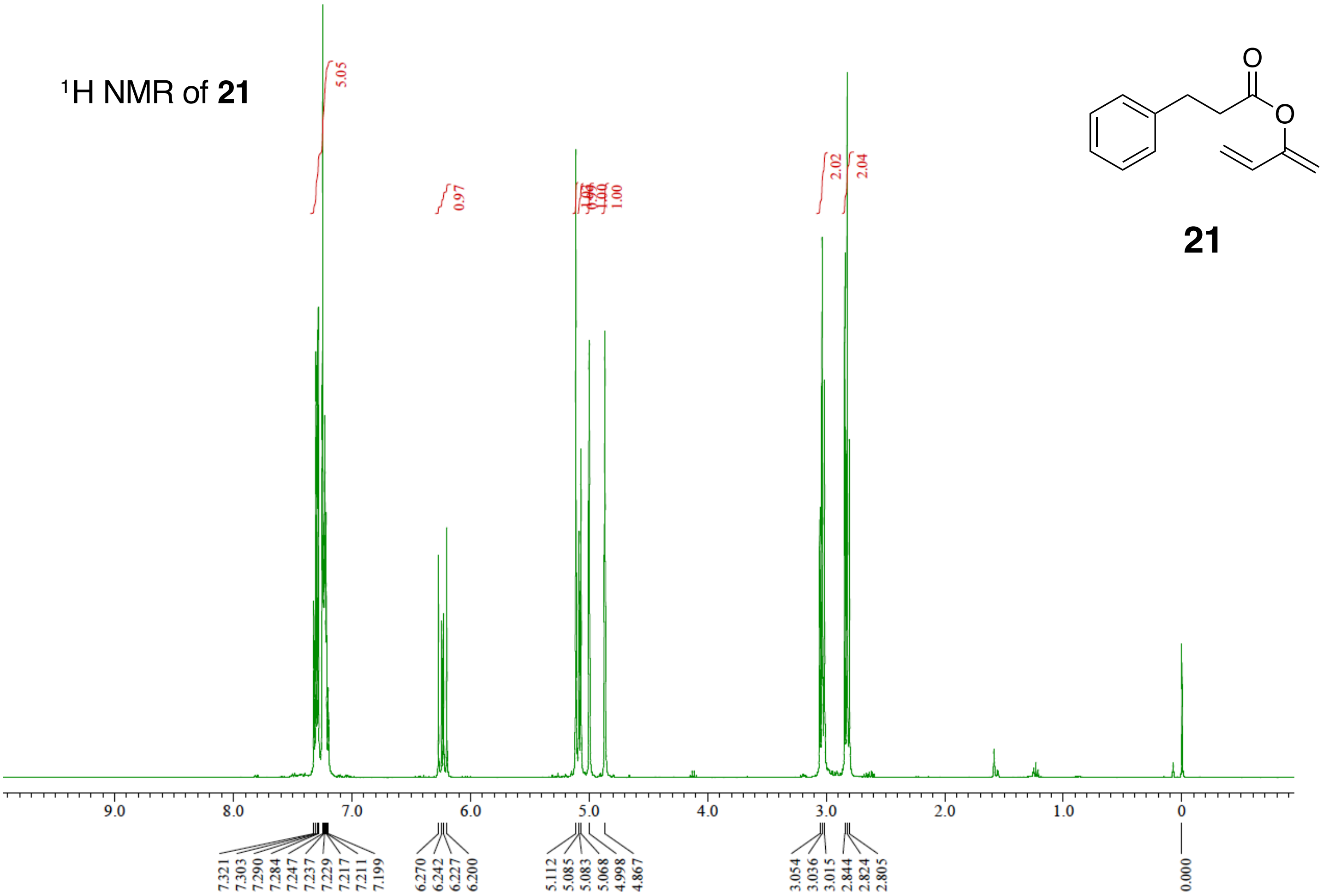

21

^{13}C NMR of **21**

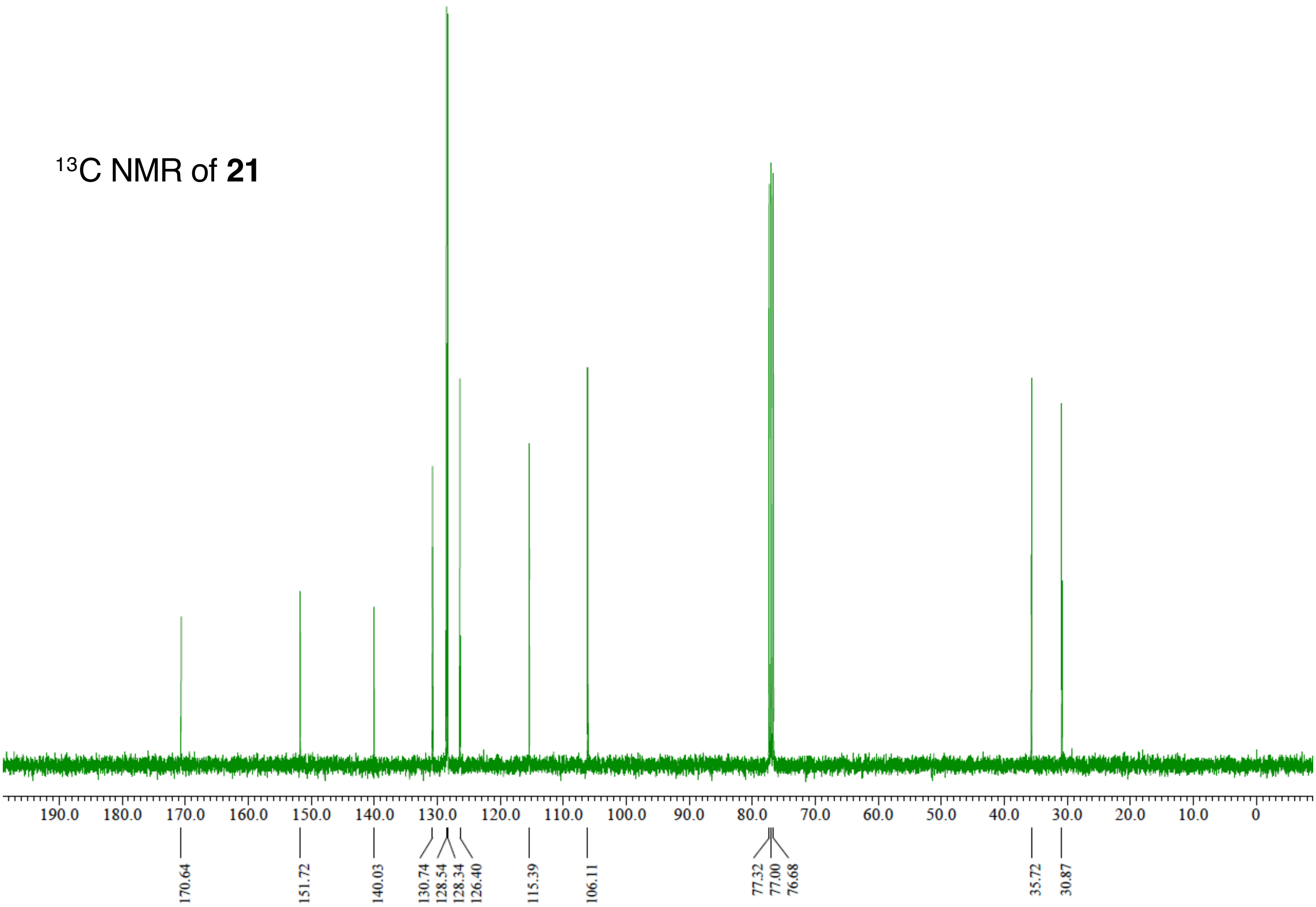

¹H NMR of **22**

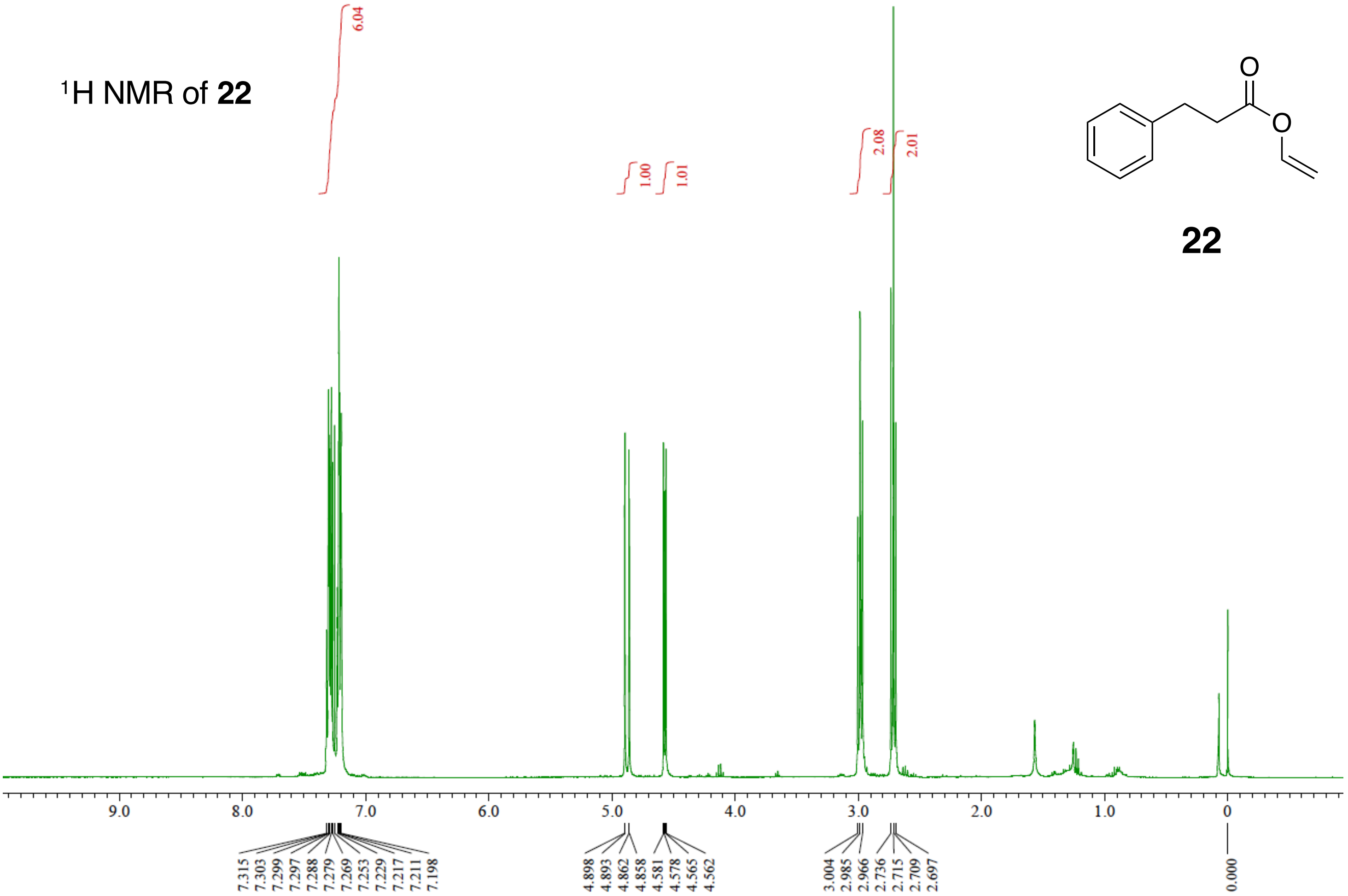

22

¹³C NMR of **22**

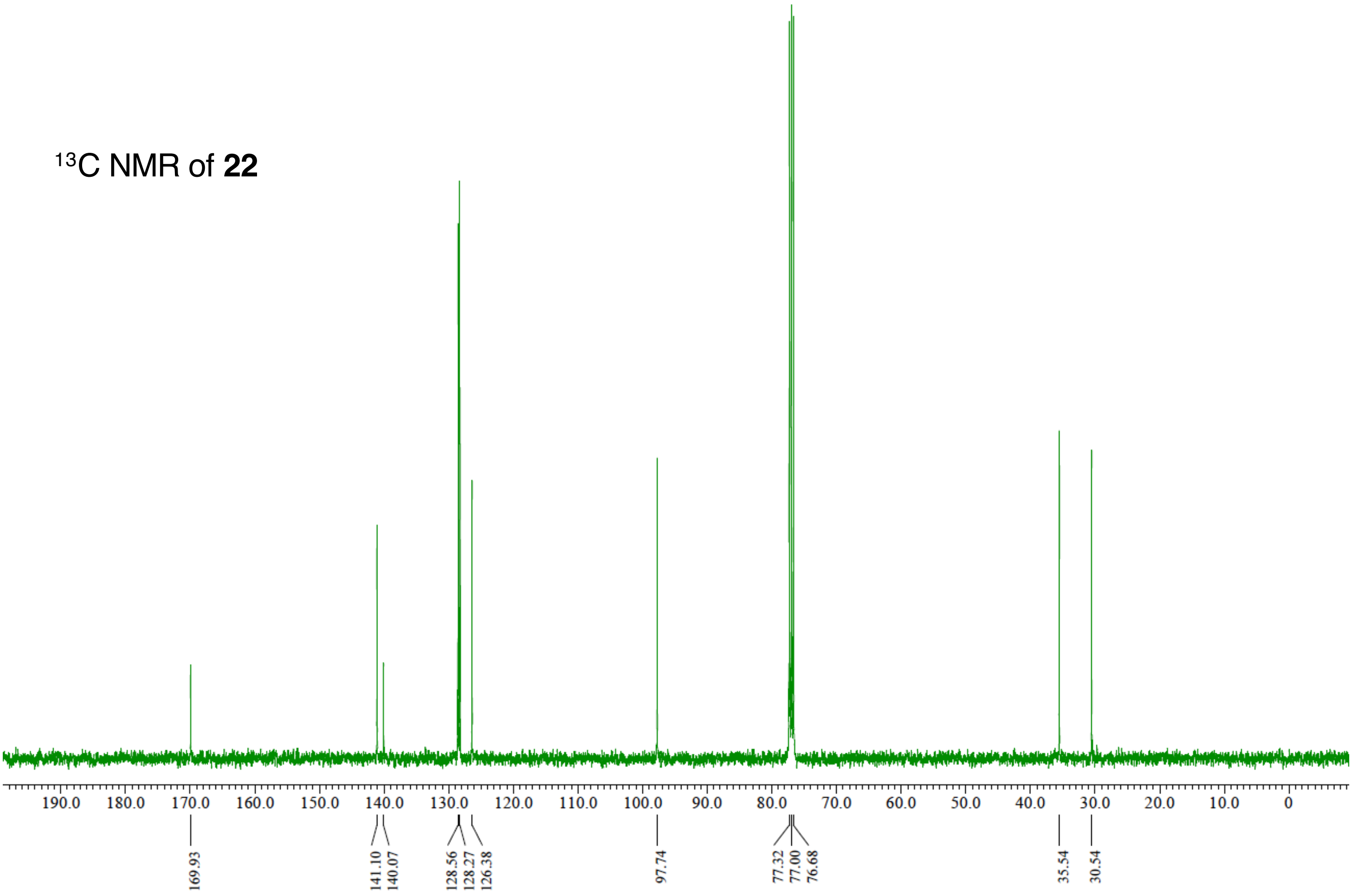

^{1}H NMR of **23**

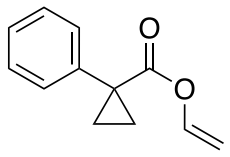

23

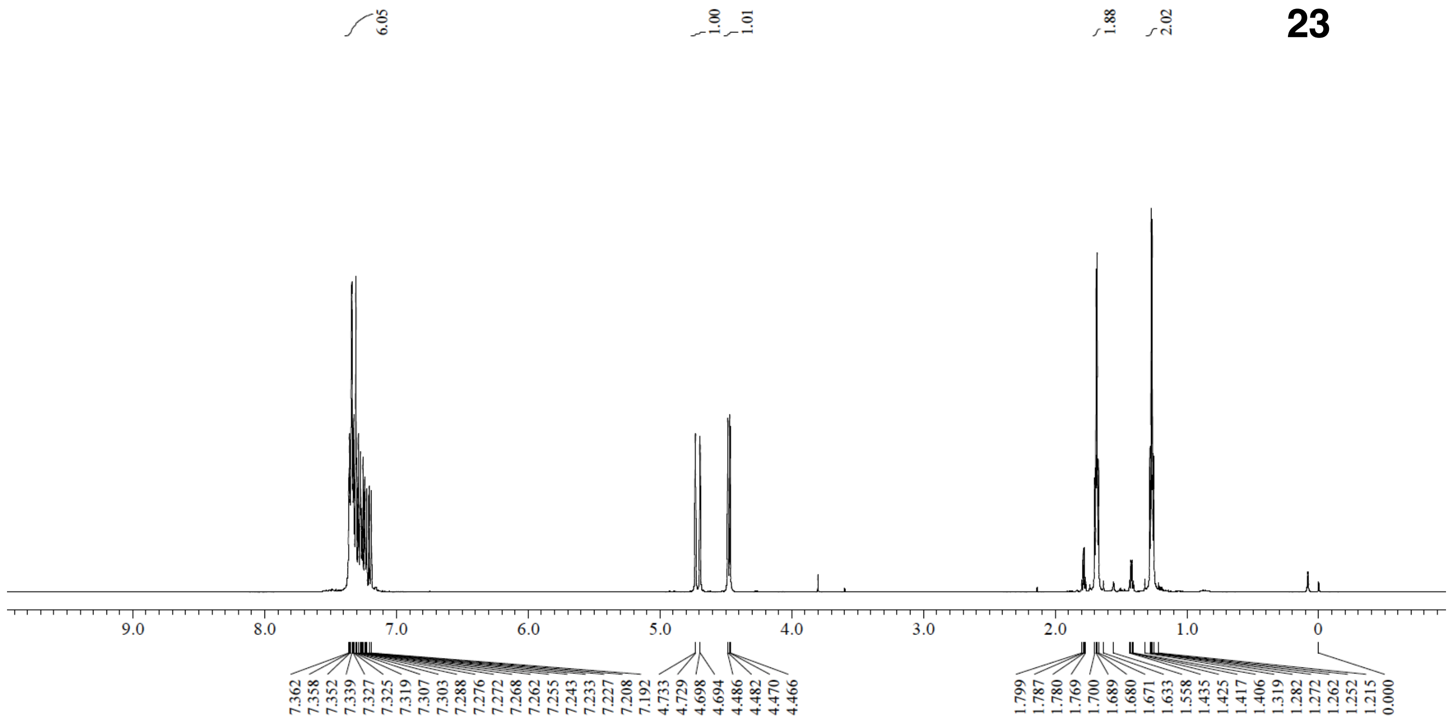

^{13}C NMR of **23**

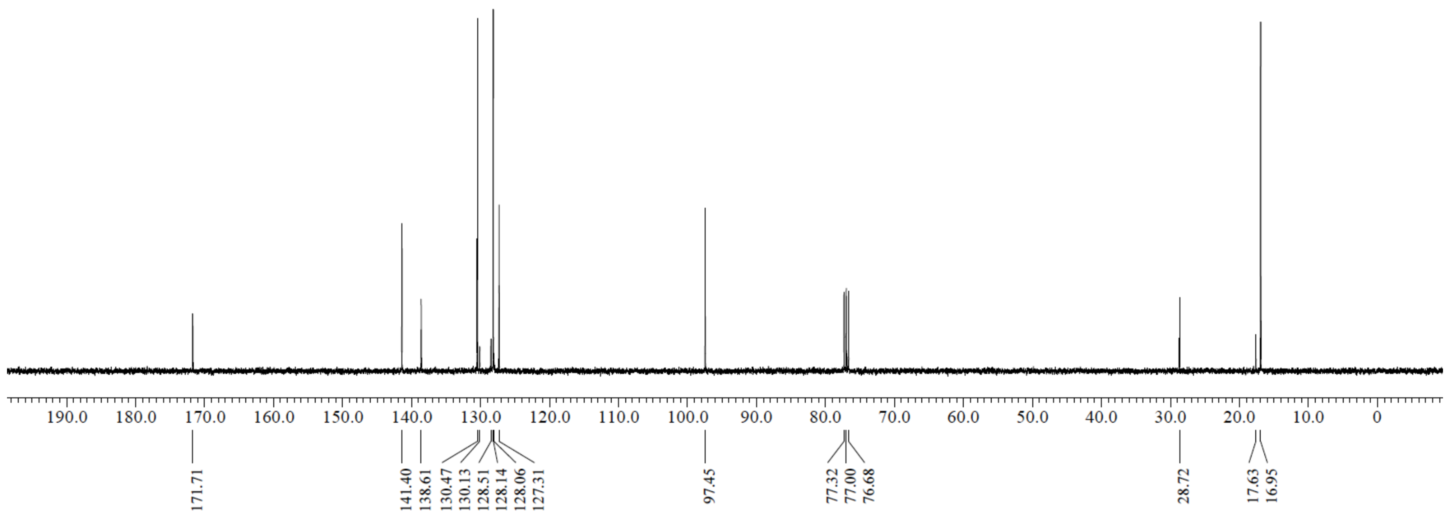

^{1}H NMR of **24**

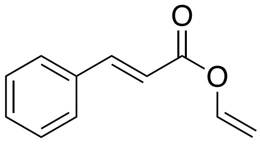

24

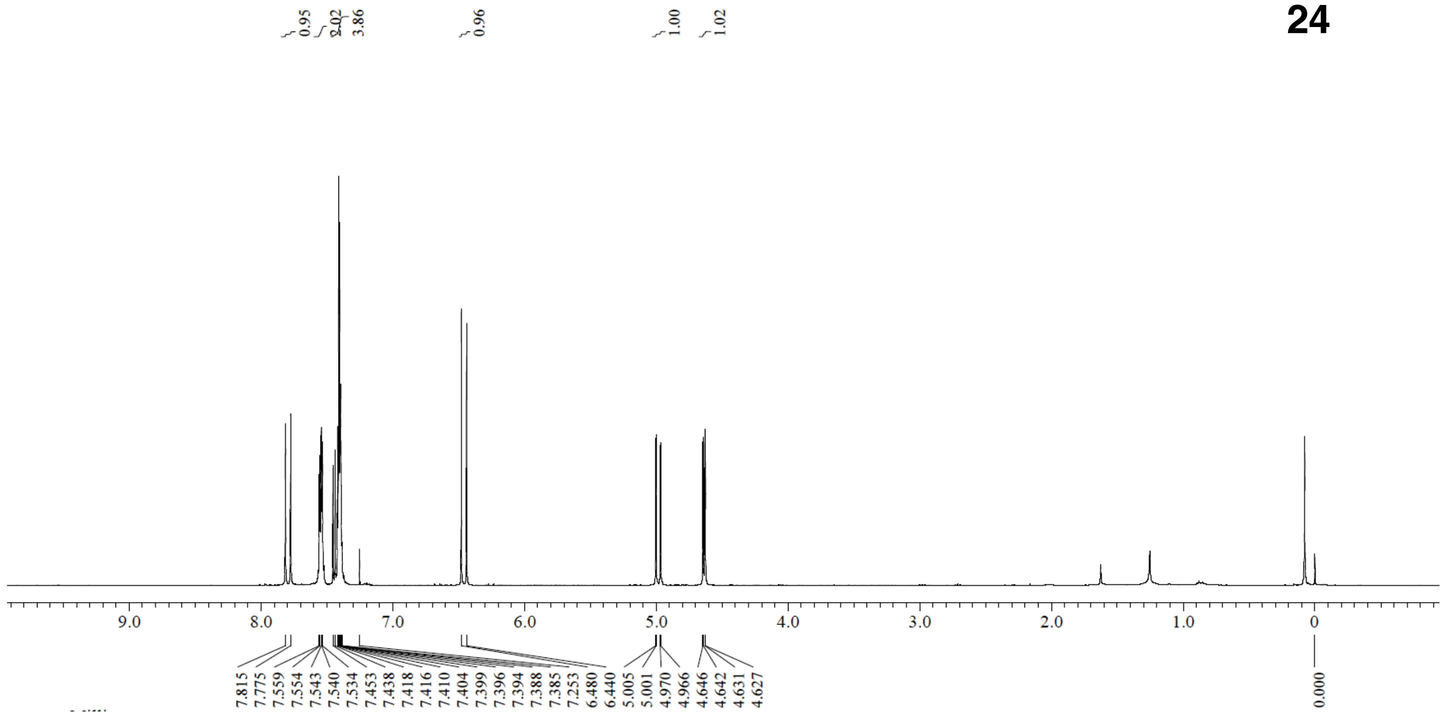

^{13}C NMR of **24**

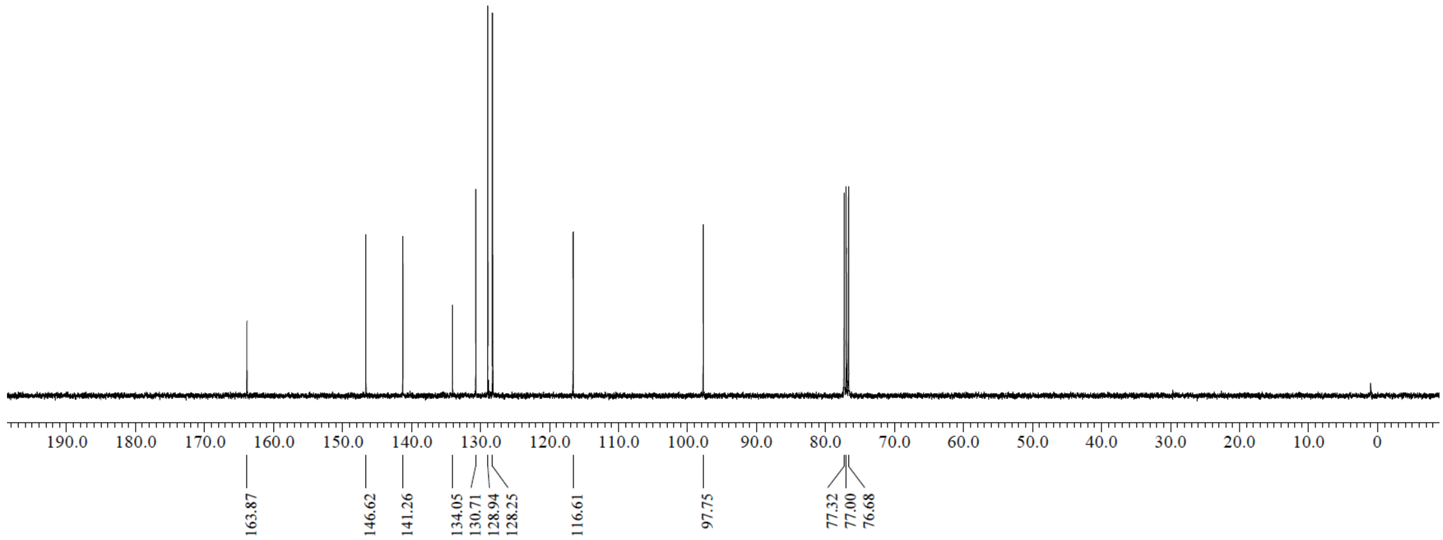

Supplement: Supplementary file 1 — Supporting Information [file OPEN-13-e202300300-s001.pdf]
